# A Sociotechnical Approach to Bring-Your-Own-Device Security in Hospitals: Development and Pilot Testing of a Maturity Model Using Mixed Methods Action Research

**DOI:** 10.2196/71912

**Published:** 2025-08-13

**Authors:** Tafheem Ahmad Wani, Antonette Mendoza, Kathleen Gray

**Affiliations:** 1 School of Psychology and Public Health Department of Public Health La Trobe University Melbourne Australia; 2 Centre for Digital Transformation of Health University of Melbourne Melbourne Australia

**Keywords:** bring your own device, BYOD, security, privacy, mobile health, mHealth, mobile phone, maturity model, sociotechnical security, policy and governance, artificial intelligence, AI

## Abstract

**Background:**

Bring your own device (BYOD) adoption in health care improves clinician productivity, but introduces cybersecurity risks due to weak security controls, human error, and policy circumvention. Existing security frameworks and models are technocentric, while overlooking sociotechnical factors such as clinician behavior, workflow integration, and organizational culture. This misalignment reduces their effectiveness in health care settings. In addition, hospitals vary in structure, resources, and BYOD use, necessitating a flexible yet structured approach to assess security maturity and prioritize improvements, which is lacking in existing models.

**Objective:**

This study aims to develop and pilot a hospital BYOD security maturity model that integrates technical, policy, and human factors for a structured assessment and improvement of BYOD security in health care.

**Methods:**

This study used mixed methods action research to design and pilot a hospital BYOD security maturity model. Surveys and interviews with IT managers and clinicians shaped the model, which was trialed at a public metropolitan hospital in Victoria, Australia. Participants completed a maturity assessment and joined a 90‑minute co‑design workshop that prioritized 6 key domains and proposed improvements. Descriptive statistics and thematic analysis guided refinements to improve clarity and usability.

**Results:**

The model was initially developed with 22 domains across 3 key dimensions: technology, policy, and people, each structured across 5 maturity levels to support systematic progression in hospital BYOD security. On the basis of participant feedback during the refinement process, 2 training-related domains were merged, resulting in a final model with 21 domains. The technology dimension includes domains such as identity, access, and authentication management; device security; and clinical communication, ensuring technical controls align with hospital policies and workflows. The policy dimension focuses on governance, covering areas such as BYOD strategy, regulatory compliance, and incident response, to establish clear security guidelines and enforcement mechanisms. The people dimension addresses human factors, including security awareness training, stakeholder involvement, and security culture, fostering staff engagement and adherence to security protocols. A maturity assessment survey conducted at a public metropolitan hospital in Victoria, Australia, revealed an overall maturity level of 2.04. Key areas for improvement included identity and access management, clinical communication security, and governance transparency. A 90-minute co-design workshop identified challenges and proposed solutions for the top 6 priority domains. Recommendations included implementing single sign-on, defining a formal BYOD strategy, enhancing secure communication tools, and improving stakeholder engagement.

**Conclusions:**

The model can serve as a valuable tool for hospitals and policy makers, offering actionable recommendations to strengthen BYOD security. The pilot implementation demonstrated its practical applicability, helping the hospital identify security gaps and develop a road map for structured enhancements. Further validation across diverse health care settings will enhance its adaptability and long-term impact.

## Introduction

### Background

Bring your own device (BYOD) is the practice of using personal devices, including laptops, smartphones, and tablets, for professional purposes. This practice has witnessed a significant increase in adoption across various sectors, especially within the health care domain [1]. A survey conducted in 2021 among hospital clinicians in Australia revealed that 87% of respondents engaged in BYOD for tasks related to their hospital responsibilities [2]. The ubiquitous and multifunctional nature of contemporary mobile devices attracts clinicians, who use BYOD for activities such as clinical documentation, accessing electronic medical records (EMRs), diagnostic decision support, and clinical communication [3-6]. For health care professionals, BYOD allows enhanced flexibility and convenience, thereby enhancing their mobility and productivity. Nevertheless, despite its benefits, BYOD is recognized as a contributor to increased cybersecurity vulnerabilities, which presents a significant concern for health care institutions [7,8]. A critical dimension of BYOD implementation is the insufficient security measures associated with clinicians’ personal devices, rendering them more susceptible to security breaches, particularly due to human error and misuse. These elements are acknowledged as key contributors to data breaches within the health care sector [7,9,10]. In certain situations, clinicians may sometimes prioritize immediate patient care over adhering to IT security protocols, further exacerbating risks [11,12]. The risk escalates within a BYOD environment, wherein users exert greater control over hospital data residing on their personal devices, thus increasing the likelihood of security oversights. For instance, the proliferation of EMR use on mobile devices intensifies the difficulty of integrating security measures into routine workflows [13,14]. Moreover, adherence to health care privacy regulations further complicates the adoption of BYOD, as hospitals strive to reconcile security requirements with clinical operational efficiency [9].

Multiple academic studies have explored security issues associated with BYOD use, addressing challenges such as device theft, network vulnerabilities, malware, unencrypted data, and improper employee behavior [15-17]. However, research in the health care sector is comparatively limited. Several researchers emphasize the need for targeted academic investigations to offer a comprehensive and objective understanding of this field. They have highlighted that a one-size-fits-all approach to BYOD security is insufficient, and existing best practices and recommendations lack the nuanced approaches needed for specialized sectors such as health care, where security solutions must balance operational efficiency with strict regulatory requirements [18,19]. The intricate nature and multifaceted dynamics of technology implementation within health care calls for focused research on BYOD security in hospital environments. On the contrary, previous research has highlighted the limitations of existing BYOD security frameworks in health care, noting a predominant focus on technical solutions without adequately addressing the sociotechnical dimensions critical in clinical environments [15,16]. Existing frameworks emphasize technical controls such as access management and encryption (the process of converting data into a coded format to prevent unauthorized access, ensuring data security both in transit and at rest) but often overlook the importance of user behavior, organizational culture, and workflow integration [17,20]. This technocentric approach has been associated with increased vulnerability to cyberattacks due to neglected social aspects of security [21,22].

Recognizing these gaps, previous work involved developing a preliminary hospital BYOD security framework that integrated sociotechnical considerations, encompassing technical controls, policies, and human factors [10]. This framework outlined a 7-stage process—plan, identify, protect, detect, respond, recover, and assess and monitor—with recommendations tailored to the unique challenges of BYOD security management in hospitals. Subsequent empirical studies involving hospital IT managers and clinicians provided practical insights, further informing and refining the framework [2,23-25].

However, translating this comprehensive framework into actionable and practical sociotechnical security controls presents challenges. Hospitals vary significantly in their organizational structures, workflows, and available resources, making it impractical to implement all recommendations uniformly. There is a need for a flexible tool that allows hospitals to assess their current BYOD security posture and prioritize improvements in a manner that aligns with their specific contexts.

Maturity models have been used in information systems as instruments for assessment and systematic enhancement of organizational practices [26,27]. In information security, maturity models aid organizations in identifying cybersecurity gaps and developing tailored countermeasures that are feasible and aligned with organizational goals [28]. Nonetheless, existing maturity models in cybersecurity are predominantly technocentric and compliance-oriented, lacking in sociotechnical considerations and specific applicability to the health care sector [26,28-32]. A 2025 systematic review of cybersecurity maturity models identified several persistent gaps, including the absence of sector-specific frameworks and the limited integration of behavioral and organizational dimensions [33]. Similarly, other contemporary studies tend to focus on national cybersecurity capabilities, broad information security themes, or narrowly defined applications, such as incident response, and hence do not address the full range of health care–specific challenges, nor do they provide comprehensive sociotechnical maturity frameworks [34-36]. The complexity of health care systems, characterized by intricate interactions between technology, clinical workflows, communication, and regulatory requirements, necessitates a maturity model that encompasses both technical and sociotechnical dimensions [37-39].

### Objectives

Given the absence of a tailored maturity model addressing hospital BYOD security and its unique challenges, this study aims to bridge this gap by developing a comprehensive and holistic hospital BYOD security maturity model. Therefore, the aim of our research is to propose and present a comprehensive hospital BYOD security maturity model. Furthermore, we describe a pilot implementation that demonstrates the model’s practical application to BYOD security in a specific hospital organizational context. In addition, the model presented aims to advance maturity model theory by offering a granular, domain-based structure that enables targeted progression mapping. This overcomes the abstraction of traditional models by operationalizing maturity in specific, interrelated hospital functions.

By bridging the gap between theoretical frameworks and practical implementation, this study aims to provide hospitals with a practical tool to enhance their BYOD security posture in a manner that is adaptable to their unique organizational contexts. The resulting maturity model is intended to facilitate a systematic approach to improving BYOD security, integrating both technical controls and sociotechnical factors, such as clinician behavior and organizational culture.

## Methods

The method section comprises 2 subsections. First, we describe the method used to develop and refine the proposed hospital BYOD security maturity model. Then, we outline the method used to pilot its implementation.

### Development and Application of the Proposed Hospital BYOD Security Maturity Model

#### Maturity Model Development

Before the maturity model, the authors developed a hospital BYOD security framework providing a comprehensive set of sociotechnical guidelines for BYOD security in hospitals across the BYOD life cycle. This framework was developed using a mixed methods action research (MMAR) approach, providing a robust foundation for the creation of the hospital BYOD (hBYOD) maturity model. The MMAR methodology enabled the integration of both qualitative and quantitative data (collected through empirical studies), ensuring that the framework addressed sociotechnical factors, including technical controls, policies, and human behaviors, to balance security with clinical productivity [40-42]. The quantitative studies involved surveys of IT management staff across hospitals to assess existing security practices and BYOD users, such as clinicians, to evaluate their security behaviors and compliance. The qualitative component included semistructured interviews with IT management staff to explore contextual factors influencing BYOD security decisions and with clinicians to understand how security measures impact their workflows and productivity. This comprehensive approach ensured that both the management and user perspectives were incorporated into the framework’s development.

For the purposes of the maturity model, recommendations were mapped from the hospital BYOD security framework to the 5 maturity levels across all domains of the model. These descriptions and levels were triangulated among the 3 researchers to ensure consistency, accuracy, and alignment with the framework. By aligning the framework’s recommendations with the maturity levels, elementary guidelines correspond to the lower levels, while more complex and advanced guidelines align with the higher levels, in accordance with the defined criteria for each level. This mapping ensures structured progression from basic to optimized practices within each domain.

**Figure 1:**
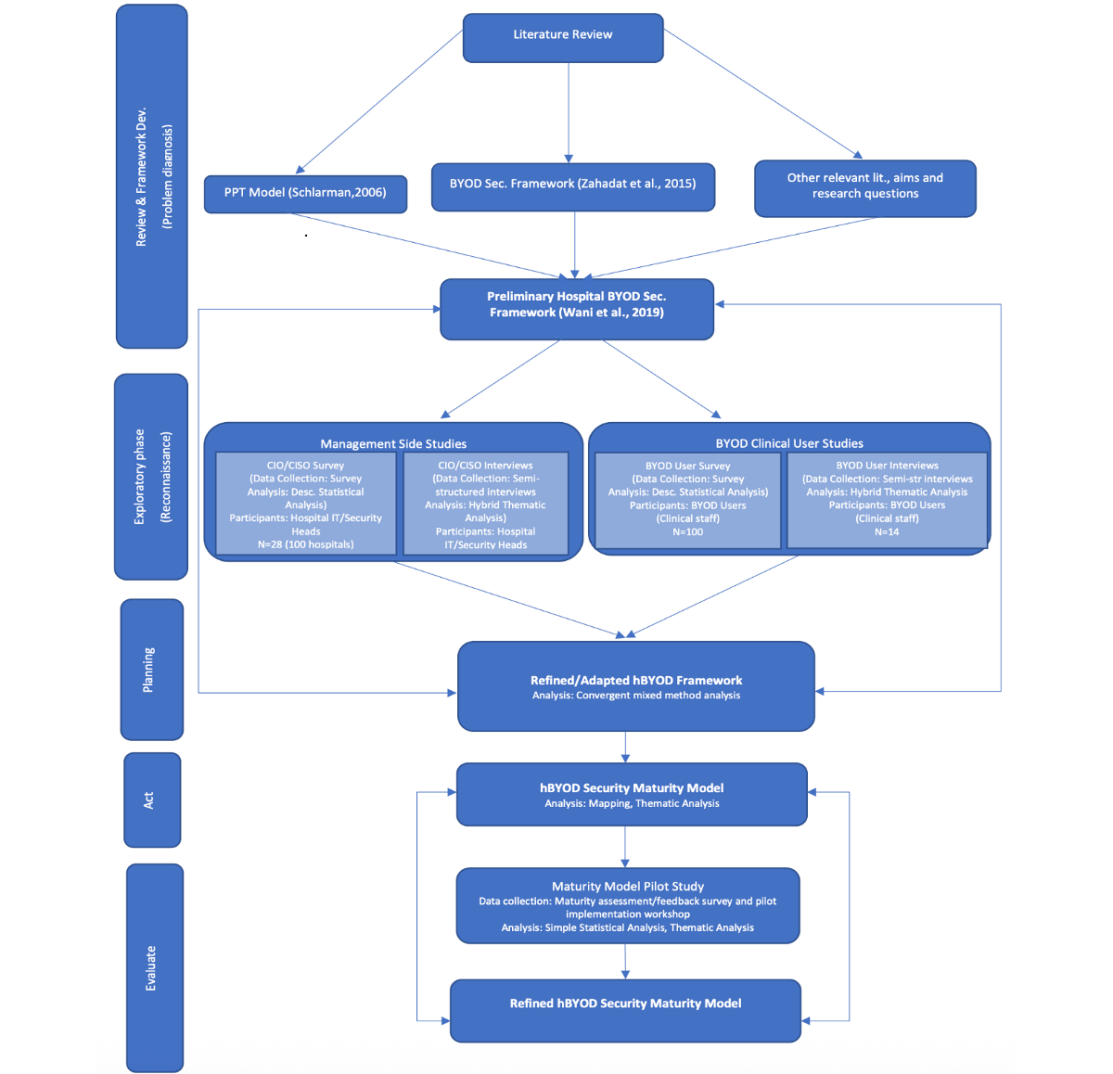
Hospital bring-your-own-device (hBYOD) security maturity model—Development process overview. BYOD: bring your own device; CIO: chief information officer; CISO: chief information security officer; PPT; people, policy, technology.

Figure 1 illustrates the comprehensive process involved in developing the hBYOD maturity model, from initial problem analysis to the finalization of the model [10,19,43].

The proposed maturity model was initially developed with 22 domains across the 3 dimensions of people, process, and technology (PPT; 7 domains for technology, 7 for policy, and 8 for people), selected for their relevance to hospital BYOD security, following extensive research that included literature reviews and a series of empirical studies [2,23-25]. On the basis of participant feedback during the refinement process, 2 training-related domains were merged, resulting in a final model with 21 domains. Each domain represents a specific aspect of hospital BYOD security.

Each domain represents a specific aspect of hospital BYOD security. Incorporating the PPT dimensions ensured a holistic sociotechnical approach when assessing BYOD security maturity [43]. Every domain is defined across 5 maturity levels, ranging from level 1 to level 5, with unique definitions for each level in all 21 domains. These levels are aligned with prominent academic and industry-based maturity models [31,44-46]. An analysis of these models reveals the following definitions corresponding to each maturity level:

1. Nonexistent or very limited and elementary implementation, unreliable, completely reliant on manual effort, and major changes required.
2. Ad hoc or informal implementation, reactive, significant room for improvement, and mostly reliant on manual effort.
3. Well-defined but with gaps in management and enforcement, minimal automation, a high degree of manual effort, compliant, and moderate room for improvement.
4. Well-managed and enforced, structured practices, moderate level of automation, proactive, moderately agile, best practice, and low room for improvement.
5. Continually improving and highly agile and real-time, high levels of automation, complete integration, enterprise-wide practices, innovative, and up to date with little or no room for improvement.

The 5 maturity levels across all domains were derived from the hospital BYOD security framework (Multimedia Appendix 1), ensuring each level has a distinct and well-defined definition and characterization. Detailed mappings of the hospital BYOD security framework’s recommendations to the maturity levels are provided in Multimedia Appendix 2.

#### Assessment Process Using the Maturity Model

The maturity model can be used to assess a hospital’s BYOD security maturity through a straightforward process. For each of the 22 domains, a brief description of cybersecurity controls or practices is provided for each of the 5 maturity levels. Assessors rate their hospital from level 1 to level 5 in each domain, based on how closely their practices align with the provided descriptions. Assessors are expected to possess substantial knowledge of the described activities and have tangible evidence of the specified security controls.

When multiple assessors evaluate the same hospital, an average rating can be calculated for each domain to determine the overall maturity level. In addition, average ratings can be computed for each of the 3 PPT dimensions, as well as an overall average, providing cumulative ratings. This approach allows the model to identify specific areas for improvement and to represent the general capability of the hospital, based on individual domain maturity ratings and the cumulative mean maturity rating, respectively.

#### Prioritization and Roadmap for Improvement

It is important to note that areas for improvement can be prioritized or ranked based on the hospital’s needs, future mission, capabilities, and feasibility considerations. Hospitals can plan a road map for enhancement by referring to the higher maturity levels in specific domains as guidance. For example, if a hospital’s maturity rating in a particular domain is level 2, the descriptions of the progressive levels (levels 3, 4, and 5) can provide actionable insights on how stepwise improvements can be achieved. Depending on the hospital’s capabilities and resources, it may aim for the level that is feasible to attain within a given time frame.

### Pilot Implementation Study

This section outlines the methodology used for the pilot implementation study.

#### Ethical Considerations

Ethics approval was obtained from the hospital’s ethics committee and the University of Melbourne, where the study was conducted (Ethics approval project no. 556/21). Informed consent was obtained from all participants through an information statement, and participation was voluntary, with an option to withdraw at any time. Survey responses were collected anonymously, and workshop data were pseudonymised and deidentified to ensure privacy and confidentiality. No patient data were collected. All data were securely stored on password-protected university systems. No compensation was offered to participants.

#### Study Site

The maturity assessment and improvement study were conducted at a major public tertiary metropolitan hospital in Victoria, Australia.

#### Study Participants and Recruitment

#### Overview

Two key groups of BYOD security stakeholders from the participating hospital were involved in this study. Participants were selected based on their involvement and understanding of BYOD security management, decision-making, or use within the hospital. The selection process was facilitated through nominations from the hospital’s chief privacy officer to ensure the inclusion of individuals with relevant expertise and diverse perspectives. The criteria for participant selection included the following.

#### Technology Managers

IT professionals, cybersecurity managers, and policy makers responsible for BYOD decision-making, such as developing or implementing BYOD security strategies. They were chosen for their expertise in technical security controls, policy formulation, and compliance measures.

#### Clinical Stakeholders (Device Users)

Clinicians who understood BYOD use and clinical needs, such as those with IT and informatics roles. They were selected to provide insights into the impact of BYOD security controls on aspects such as clinical efficiency, usability, and compliance with hospital policies.

Nominated individuals were provided with a plain language statement and were included in the study upon giving informed consent. A total of 10 participants from the hospital took part in the study.

#### Study Design

The study was conducted in 2 stages.

#### Maturity Assessment and Model Feedback Survey (Before the Workshop)

A BYOD security maturity assessment and feedback survey was developed using Qualtrics (Qualtrics International Inc; Multimedia Appendix 3). Participants received a survey link and had 1 week to complete it independently before the workshop. Both technology managers and clinical stakeholders assessed BYOD security maturity by selecting the most appropriate level for each domain based on their knowledge and available tangible evidence. They also provided feedback on the accuracy, applicability, and clarity of the model, with an option to suggest improvements or indicate if they lacked sufficient knowledge to assess a domain.

Following the maturity assessment, participants were asked for their reflective comments on the structure of the model. This included (1) identifying any missing domains that should be included, (2) suggesting the removal of any domains deemed unnecessary, and (3) proposing reclassification of domains from one dimension to another.

At the end of the survey, participants were asked to prioritize and rank the domains within each PPT dimension based on their perceived importance for improving the BYOD security maturity of the hospital.

#### Maturity Improvement Discussion Workshop

Following the maturity assessment, a 90-minute Zoom (Zoom Communications, Inc) workshop was held with the same survey participants, including IT and clinical representatives providing management and user perspectives on BYOD use.

A participatory co-design approach was used during the workshop, enabling these stakeholder groups to collaboratively discuss challenges and propose recommendations related to the prioritized domains of BYOD security identified from the initial survey [47,48]. To maximize the effectiveness of the workshop, design and evaluation guidelines by Thoring et al [49] were followed.

The web-based workshop tool MURAL was used to document participant responses. Each group had 2 facilitators who managed time, addressed queries, and recorded key discussions. The full workshop session was recorded, transcribed, and deidentified.

#### Analysis

The maturity assessment data underwent descriptive statistical analysis to determine mean maturity ratings across the hospital’s BYOD security domains. The top 6 priority domains (two from each of the technology, policy, and people dimensions) were identified based on participant responses.

For qualitative analysis, data from the maturity assessment, model improvement feedback, and workshop sessions were analyzed using thematic analysis [50]. A deductive approach was applied to assess how participant feedback aligned with predefined BYOD security maturity domains, followed by an inductive review to identify emerging challenges, contextual insights, and proposed solutions. This combined approach ensured that both structured evaluation and exploratory findings contributed to the refinement of the model and the formulation of consolidated, prioritized security recommendations for the hospital.

#### Participant Characteristics

A total of 10 participants from the model hospital, representing both participant groups—7 technology managers and 3 clinical stakeholder representatives—participated in the maturity assessment survey and the following discussion workshop.

**Figure 2:**
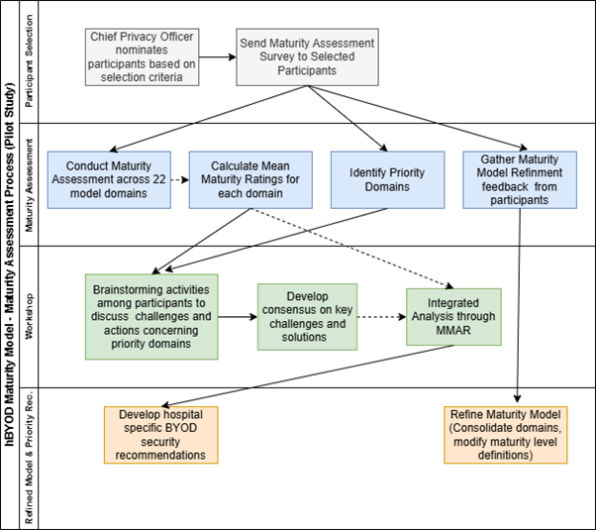
Maturity assessment and model refinement process overview. BYOD: bring your own device; hBYOD: hospital bring your own device; MMAR: mixed methods action research.

Figure 2 provides an overview of the maturity assessment and model refinement process.

## Results

This section is organized into three parts: (1) maturity model description, (2) feedback and refinements, and (3) hospital maturity assessment and workshop recommendations. The first part provides an overview of the maturity model domains, the second summarizes participant feedback and resulting revisions, and the third presents the findings from the maturity assessment survey and subsequent workshop conducted at the model hospital.

### Maturity Model Description

#### Overview

The proposed maturity model was initially developed with 22 domains across 3 dimensions—technology (7 domains), policy (7 domains), and people (8 domains). Following participant feedback, 2 training-related domains under the people dimension were merged, resulting in a final model comprising 21 domains. This revision is detailed in the Feedback and Refinements subsection.

While this section provides only a brief overview of the 3 dimensions and their respective domains, the complete model, including detailed definitions for each of the 5 maturity levels per domain, along with clearly defined, measurable criteria, is presented in Multimedia Appendix 2. To aid interpretation, the operational indicators for each level have been bolded in the revised version of the appendix. These indicators are designed to support objective evaluation and guide structured improvement efforts.

#### Technology

#### Overview

The technology dimension demonstrates a clear progression from basic security practices to an advanced, integrated sociotechnical approach, where technology aligns with organizational policies and user needs. Each maturity level builds on the previous, addressing specific security and usability challenges relevant to BYOD use in hospitals. Table 1 presents a concise summary of the technology domains, offering a high-level overview of their structure and key focus areas.

**Table 1:** Technology dimension domain definition and descriptions (7 domains).

Technology dimension	Definition and description	Levels summary
Identity, access, and authentication management	Technologies and processes for verifying user identity and controlling access to hospital systems and data in a BYOD^a^ environment	Level 1: basic password-only authentication without additional protections for BYOD access. Level 2: complex passwords with enforced policies, providing minimal improvement for personal device security. Level 3: dual-factor authentication (eg, OTP or token) to enhance security for BYOD users. Level 4: RBAC^b^ tailored to BYOD device use, restricting access based on job roles. Level 5: federated SSO^c^ for seamless and secure access across multiple hospital systems from BYOD devices.
Storage and backup	Tools and controls for securely storing and backing up hospital data accessed or stored on BYOD devices, ensuring separation from personal data	Level 1: no controls for securing or backing up data accessed via BYOD devices. Level 2: minimal separation between personal and hospital data, with limited backup options for BYOD users. Level 3: virtualizationd ensures BYOD users access data in secure, isolated environments without local storage. Level 4: containerizatione fully separates hospital data on BYOD devices, with secure backup options. Level 5: private cloud infrastructure with automated, encrypted backups for data accessed through BYOD devices.
Device security	Security measures to protect BYOD devices, including encryption^f^, antivirus, and compliance monitoring	Level 1: no restrictions or security requirements for BYOD devices. Level 2: manual security controls, such as user-installed antivirus and basic settings. Level 3: blacklist and white list capabilities to restrict unauthorized apps and ensure BYOD compliance. Level 4: end point protectiong with real-time threat detection and device monitoring for BYOD devices. Level 5: UEMh for centralized, automated security management of all BYOD devices.
Network security	Security protocols to protect hospital networks from threats introduced by BYOD use, including firewalls and segmentation	Level 1: basic firewalls providing limited protection against BYOD-related threats. Level 2: network segmentation separates hospital systems from BYOD devices to minimize risk. Level 3: firewalls integrated with IDSi and IPSj) to identify and mitigate BYOD-originated threats. Level 4: secure web gatewaysk filter internet traffic from BYOD devices, ensuring safe access. Level 5: real-time threat detection using AIl and analytics to proactively secure hospital networks from BYOD threats.
Application security	Safeguards to secure applications in a BYOD environment, preventing unauthorized access and data breaches	Level 1: no restrictions, allowing any applications on BYOD devices without security oversight. Level 2: limited to basic applications with minimal security checks for BYOD use. Level 3: vulnerable silos where hospital apps on BYOD gadgets lack integration or adequate oversight. Level 4: approved white-listed applications with robust security policies for BYOD use. Level 5: virtualized applications providing isolated and secure environments for BYOD users.
BYOD management and automation	Systems and tools to manage, monitor, and automate security measures for BYOD devices within the hospital	Level 1: manual BYOD management with no centralized tools. Level 2: ad hoc controls implemented reactively for BYOD issues. Level 3: MDMm solutions enforce basic policies for BYOD compliance. Level 4: MAMn tools control apps and permissions on BYOD gadgets. Level 5: UEM provides comprehensive, automated security and operational management for BYOD gadgets.
Clinical communication, photography, and file sharing	Secure platforms and protocols for communication, photography, file sharing, and data transfer using BYOD devices in clinical settings	Level 1: undefined systems, with personal apps frequently used on BYOD devices for communication. Level 2: generic communication platforms with limited security features for BYOD users. Level 3: limited secure platforms partially integrated into hospital workflows for BYOD communication. Level 4: fully integrated platforms with strong encryption and compliance features tailored for BYOD devices. Level 5: dedicated systems specifically designed for secure clinical communication and file sharing on BYOD devices.

^a^BYOD: bring your own device.

^b^RBAC: role-based access control. A method of restricting system access based on an individual’s job role to enforce security and limit exposure to sensitive data.

^c^SSO: single sign-on. An advanced form of SSO that allows users to access multiple apps and systems across different organizations or domains using a single authentication mechanism.

^d^A technology that allows hospital apps and data to be accessed in a controlled, virtual environment rather than being stored directly on BYOD devices, reducing security risks and enhancing centralized management.

^e^A security method that isolates hospital data and apps within a secure, controlled environment on a BYOD device, preventing unauthorized access to sensitive information while maintaining separation from personal apps.

^f^The process of converting data into a coded format to prevent unauthorized access, ensuring data security both in transit and at rest.

^g^End point protection: protection measures applied to end-user devices (eg, mobile phones, laptops) to prevent privacy or security breaches.

^h^UEM: unified end point management. A comprehensive security solution to managing and securing all devices (eg, desktops, mobiles, and tablets) from a single platform.

^i^IDS: intrusion detection system. Security tools are designed to detect, alert, and prevent potential cyberattacks by monitoring network traffic and system activities.

^j^IPS: intrusion prevention system.

^k^A security solution that monitors and filters internet traffic from devices to enforce hospital security policies, block malicious websites, prevent data leaks, and protect against web-based threats.

^l^AI: artificial intelligence.

^m^MDM: mobile device management. A security solution that allows organizations to control, monitor, and enforce policies on personal and organization-owned mobile devices.

^n^MAM: mobile application management. A security solution that focuses on controlling and securing hospital-approved apps on BYOD devices without managing the entire device, enabling secure access to clinical apps while maintaining user privacy.

The following sections provide a brief description of the 5 maturity levels across the 7 technology domains, illustrating the progression from minimal to advanced security capabilities and their application in BYOD contexts within hospitals.

#### Level 1: Minimal Security Controls

This foundational level exhibits basic security with limited controls. Authentication is password-based, without advanced access restrictions or separation between hospital and personal data on devices. Device security is minimal, allowing unrestricted personal device use, and network protection relies on basic firewall configurations. Application security is nonexistent, with employees using personal, unsecured applications. There are no centralized controls over BYOD use, and clinical communication occurs through personal apps, such as WhatsApp (Meta Platforms, Inc), posing significant data privacy and security risks.

#### Level 2: High Complexity and Minimal Automation

Security capabilities improve slightly, introducing single-factor authentication with complex passwords and limited device-level restrictions, mainly for high-risk devices. Network segmentation and guest network access are applied to separate personal and hospital data minimally, but data security largely depends on manual user management. BYOD management remains largely manual, and clinical communication continues through generic applications, offering little dedicated security.

#### Level 3: Basic Access Controls

At this level, more structured controls emerge, including basic identity and access management (IAM; a framework of policies and technologies ensuring that only authorized individuals can access specific hospital resources and systems) solutions with application-level virtualization (a technology that allows hospital applications and data to be accessed in a controlled, virtual environment rather than being stored directly on BYOD, reducing security risks and enhancing centralized management) for limited access control. Device security uses blacklist and white list capabilities to enforce some restrictions, while network security is strengthened with advanced firewalls and intrusion detection systems (security tools designed to detect, alert, and prevent potential cyberattacks by monitoring network traffic and system activities) and intrusion prevention systems. Although personal cloud options are permitted for backup, there is some degree of logical separation between personal and hospital data. Basic mobile device management (MDM; a security solution that allows organizations to control, monitor, and enforce policies on personal and organization-owned mobile devices) tools are used for BYOD control and secure clinical communication platforms are available, although uptake remains minimal due to limited usability and integration.

#### Level 4: Role Based and Partially Integrated

This level introduces advanced IAM solutions with role-based access control, supporting more sophisticated, role-specific permissions. Data storage is managed through sanctioned cloud platforms with access restrictions based on user roles, enhancing data protection. End point protection (protection measures applied to end-user devices [eg, mobile phones and laptops] to prevent privacy or security breaches) incorporates network visualization and secure web gateways (a security solution that monitors and filters internet traffic from devices to enforce hospital security policies, block malicious websites, prevent data leaks, and protect against web-based threats), providing greater oversight of device security. Hospital-approved applications are made accessible through a secure app store, and mobile application management (MAM; a security solution that focuses on controlling and securing hospital-approved applications on BYOD devices without managing the entire device, enabling secure access to clinical apps while maintaining user privacy) tools enable more consistent BYOD management. Dedicated platforms for clinical communication offer basic integration with hospital systems, supporting more secure and streamlined workflows.

#### Level 5: Advanced, Integrated Security

The highest maturity level reflects an enterprise-level approach with fully integrated, automated security practices. IAM solutions use artificial intelligence for enhanced authentication management, including federated single sign-on (SSO; an advanced form of SSO that allows users to access multiple apps and systems across different organizations or domains using a single authentication mechanism) across all hospital applications, significantly improving user experience and security. Storage and backup solutions are handled via a private cloud with complete data isolation, ensuring data security. Comprehensive end point protection and real-time network monitoring provide robust defense against cybersecurity threats. Application security includes sophisticated isolation and restriction features, protecting sensitive data. Unified end point management (a comprehensive security solution to managing and securing all devices [eg, desktops, mobiles, and tablets] from a single platform) enables centralized, automated control over all BYOD devices, and dedicated clinical communication platforms integrate seamlessly with hospital systems, supporting efficient and secure communication workflows.

#### Policy

#### Overview

The policy dimension outlines a structured progression in BYOD policy development and governance within health care organizations. This dimension emphasizes the progression from a lack of structure in BYOD policy and security management to a comprehensive, proactive governance framework that aligns legal, technical, and operational elements of BYOD security within health care environments. Table 2 provides a brief overview of the policy domains.

**Table 2:** Policy dimension: domain definition and descriptions (7 domains).

Policy dimension	Definition and description	Levels summary
BYOD^a^ strategy	The overarching plan for integrating BYOD into hospital workflows while ensuring security and compliance	Level 1: no defined strategy for BYOD use. Level 2: BYOD addressed informally as part of general IT strategy. Level 3: a dedicated BYOD strategy focusing on security and compliance. Level 4: comprehensive strategy covering clinical and nonclinical workflows related to BYOD use. Level 5: proactively updated strategy aligned with evolving threats and hospital needs.
Policy components	Key elements of policies that define acceptable use, privacy, and security requirements for BYOD use	Level 1: no specific BYOD policy components defined. Level 2: basic acceptable use guidelines included in broader IT policies. Level 3: dedicated BYOD policy components addressing security and privacy. Level 4: comprehensive policies tailored for BYOD workflows and compliance requirements. Level 5: regularly reviewed and updated policies integrating feedback and regulatory changes.
Regulatory compliance	Ensuring BYOD use adheres to health care regulatory standards and data security requirements	Level 1: no understanding or monitoring of regulatory requirements for BYOD. Level 2: minimal awareness of applicable regulations for BYOD use, with inconsistent adherence. Level 3: regulatory requirements are clearly defined and communicated. Level 4: compliance is routinely monitored and documented. Level 5: automated systems ensure real-time adherence to regulatory standards for BYOD devices.
Policy enforcement	Mechanisms to monitor and ensure compliance with BYOD policies, including audits and corrective actions	Level 1: no enforcement mechanisms for BYOD policies. Level 2: limited BYOD monitoring with occasional audits. Level 3: formal processes for BYOD monitoring and periodic reviews. Level 4: balanced enforcement system integrating penalties and incentives. Level 5: automated, real-time monitoring and enforcement of BYOD policies.
Incident response	Procedures for managing and resolving security incidents involving BYOD devices	Level 1: no formal incident response processes for BYOD Level 2: general IT incident management applies inconsistently to BYOD Level 3: defined BYOD-specific incident response plans Level 4: formalized processes with clear roles and rapid response capabilities Level 5: automated, proactive incident response systems for BYOD threats
Lost device policy	Guidelines for handling lost or stolen BYOD devices, including data recovery and remote wiping	Level 1: no procedures for lost or stolen BYOD devices Level 2: staff follow ad hoc guidance for reporting and recovery Level 3: defined policy for data wiping and device recovery Level 4: selective data wiping to protect sensitive information Level 5: real-time device tracking and automated data wiping capabilities
Accountability and governance	Defined structures and roles for overseeing and ensuring accountability for BYOD security	Level 1: no defined roles or governance for BYOD security Level 2: informal BYOD governance with limited oversight Level 3: BYOD governance exists but is included in general IT management Level 4: formal structures with clear roles and responsibilities for BYOD oversight Level 5: agile BYOD governance processes integrated into organizational leadership and IT strategy

^a^BYOD: bring your own device.

The following subsections outline the progression of maturity levels within the policy dimension.

#### Level 1: Undefined BYOD Policy

At this foundational level, there is no formal BYOD strategy or dedicated policy for IT security concerning BYOD. Legal risks are not identified, and there are no established processes for enforcement, measurement, or compliance verification. Incident management is ad hoc and slow, with no defined roles or responsibilities for BYOD governance, making the organization highly vulnerable to security incidents.

#### Level 2: Basic, Informal Policy

BYOD policy is incorporated into the general IT strategy, with only loosely defined BYOD security aspects and references to legislation. Legal risks are vaguely defined, and compliance verification is minimal, with a general incident management process lacking specific standard operating procedures for BYOD incidents. Lost or stolen devices are not addressed, and governance is informal, lacking a clear structure and authority.

#### Level 3: Defined Policy With Basic Governance

A dedicated BYOD policy is established, offering high-level guidance. Legal risks are identified, but they remain complex and difficult for the staff to understand. There is basic enforcement, with a high-level compliance verification process. A BYOD incident management process is defined, but response times are slow, and devices are only wiped out when it is lost or stolen. Governance is somewhat integrated, with BYOD security part of the general IT and security governance framework.

#### Level 4: Comprehensive Policy With Formal Governance

At this level, a comprehensive BYOD strategy covers both clinical and nonclinical aspects, with well-defined legal risks that are regularly monitored. Compliance is measured through a balanced system, and a defined BYOD incident management process is in place, supported by a dedicated security team. The policy allows for selective data wiping on lost devices, and a formal BYOD governance structure is established to ensure accountability and clarity in roles.

#### Level 5: Advanced, Proactive Policy

The highest maturity level features a fully developed and continually updated BYOD strategy that is aligned with the latest threat, business, and legal landscapes. Compliance verification is automated with real-time measurement. The incident management process is highly automated and proactive, with swift responses and advanced capabilities. Governance is robust, ensuring that all aspects of BYOD policy are tightly managed within a well-defined structure, allowing for adaptability and scalability in response to evolving security needs.

#### People

#### Overview

The people dimension emphasizes the evolution from minimal awareness and isolated efforts to a fully integrated, proactive security culture. As maturity increases, the organization’s workforce becomes more knowledgeable, engaged, and adept at managing BYOD security, aligned with the organization’s overarching goals for patient safety and data protection in health care environments. Table 3 provides a brief overview of the people domains:

The following subsections outline the progression of maturity levels within the people dimension.

**Table 3:** People dimension: domain definitions and descriptions (8 domains).

People dimension	Definition and description	Levels summary
BYOD^a^ security awareness and training	Programs to educate staff on the risks and security best practices specific to BYOD device use	Level 1: no awareness programs or training for staff Level 2: generic training offered occasionally, with minimal focus on BYOD security Level 3: dedicated BYOD training programs addressing basic security risks Level 4: comprehensive, role-specific training with practical scenarios for BYOD security Level 5: regularly updated, workflow-integrated training emphasizing BYOD best practices
Training dissemination	Methods and channels for delivering BYOD-specific security training to staff effectively	Level 1: no structured BYOD training dissemination Level 2: periodic reminders or basic presentations Level 3: online modules accessible to staff, covering BYOD security Level 4: multichannel training, including workshops and hands-on sessions tailored to staff roles Level 5: integrated dissemination through various media, including just-in-time training tools
Training importance	Emphasizing the role of training in maintaining security when using BYOD devices in the hospital environment	Level 1: BYOD training not prioritized or mandatory Level 2: training is optional and provided only to interested staff Level 3: training viewed as important and made available to all staff Level 4: training made mandatory with management oversight Level 5: training incentivized and fully embedded in hospital practices, with performance linked to adherence
Management support	Leadership commitment and staff support to prioritizing and funding for BYOD security	Level 1: no visible support from leadership for BYOD security initiatives Level 2: limited acknowledgment of BYOD security risks Level 3: moderate support, with funding allocated for basic BYOD security measures Level 4: high-level support with strategic investments in BYOD security Level 5: unrestricted commitment, with BYOD security as a priority in organizational strategy
Stakeholder involvement	Engagement of key stakeholders, including clinical staff and IT teams, in shaping BYOD security strategy, practices, and procedures	Level 1: no involvement of stakeholders in BYOD decisions Level 2: minimal IT department involvement without clinical input Level 3: limited leadership and stakeholder input in shaping BYOD policies Level 4: regular feedback and participation from clinical and IT teams Level 5: continuous collaborative engagement of stakeholders in all BYOD-related strategies and practices
Security culture	Promoting a proactive organizational approach to securing hospital and personal data in a BYOD environment, fostering awareness and collaboration	Level 1: no emphasis on security culture; reactive approach to incidents Level 2: awareness limited to certain departments Level 3: general staff awareness of security risks but minimal collaboration Level 4: proactive initiatives to promote a security-conscious culture across the organization Level 5: continuous improvement and organization-wide security commitment embedded into the culture
Usability and productivity	Balancing security measures for BYOD devices with ease of use to enhance clinical workflows and staff efficiency	Level 1: BYOD security measures such as extensive restrictions and complex passwords, impede usability, leading to workarounds Level 2: basic usability improvements, such as reduced sign-on, implemented without significant productivity gains Level 3: improved usability through simplified processes such as basic sign-on Level 4: workflow-optimized BYOD security measures, such as single sign-on and enterprise productivity BYOD apps Level 5: fully integrated, seamless user experience enhancing both security and productivity through an optimized, secured, and unrestrictive BYOD environment
Expertise and skills improvement	Building and maintaining technical skills to manage and secure BYOD devices effectively	Level 1: no focus on BYOD-related technical skills Level 2: limited skill-building through ad hoc initiatives Level 3: dedicated IT staff receive basic BYOD security training Level 4: regular training and certifications provided for key staff Level 5: advanced, ongoing skill development programs to maintain high levels of BYOD expertise

^a^BYOD: bring your own device.

#### Level 1: Limited Awareness and Reactive Approach

At this initial stage, there is no formal IT security training for staff regarding BYOD. Employees rely solely on personal knowledge and awareness, without any structured support or guidance from management on cybersecurity issues. There is a lack of stakeholder involvement in the BYOD strategy, leading to a restrictive environment with low expertise and inadequate capacity to handle BYOD-related security issues.

#### Level 2: Basic Guidance With Minimal Organizational Support

This level introduces optional, generic IT security training, with basic guidelines and reminders sent to staff. Management provides limited support for cybersecurity initiatives, with only IT or executive groups involved in BYOD strategy development. The organization operates within a reactive security culture, where employees create workarounds due to limited resources. Individual staff members handle BYOD security issues independently, rather than through coordinated departmental efforts.

#### Level 3: Structured Training With Moderate Stakeholder Engagement

A dedicated BYOD security training program is in place, prioritizing cybersecurity with moderate management support. Clinical leaders play a minimal role in BYOD security decision-making, and there is general awareness among staff about the importance of patient data protection. However, resistance to change exists within some groups. Basic features, such as SSO, are introduced to simplify workflows, and dedicated IT personnel manage BYOD security.

#### Level 4: Integrated Security Culture With High Stakeholder Involvement

BYOD security training becomes a mandatory, strategic, and practical component of the organization’s cybersecurity approach, with a high level of commitment and active management support. Clinical stakeholders, including BYOD users, are consulted in strategy development and changes, fostering a proactive security culture. The organization demonstrates strong capabilities to manage BYOD-related sociotechnical issues and leverages enterprise productivity features to enhance security and efficiency.

#### Level 5: Tailored Support and Proactive, Adaptive Security Culture

At the highest maturity level, BYOD security training is regularly updated and tailored to align with specific workflows, making cybersecurity a top priority. There is ongoing, unrestricted support from management, along with a collaborative process involving all stakeholders in BYOD strategy development. The organization promotes a proactive and continually improving security culture, where security concerns are autonomously managed through skilled improvement programs. This level represents a mature, integrated approach where the workforce is fully engaged and prepared to handle BYOD security challenges effectively.

#### Feedback and Refinements

**Figure 3:**
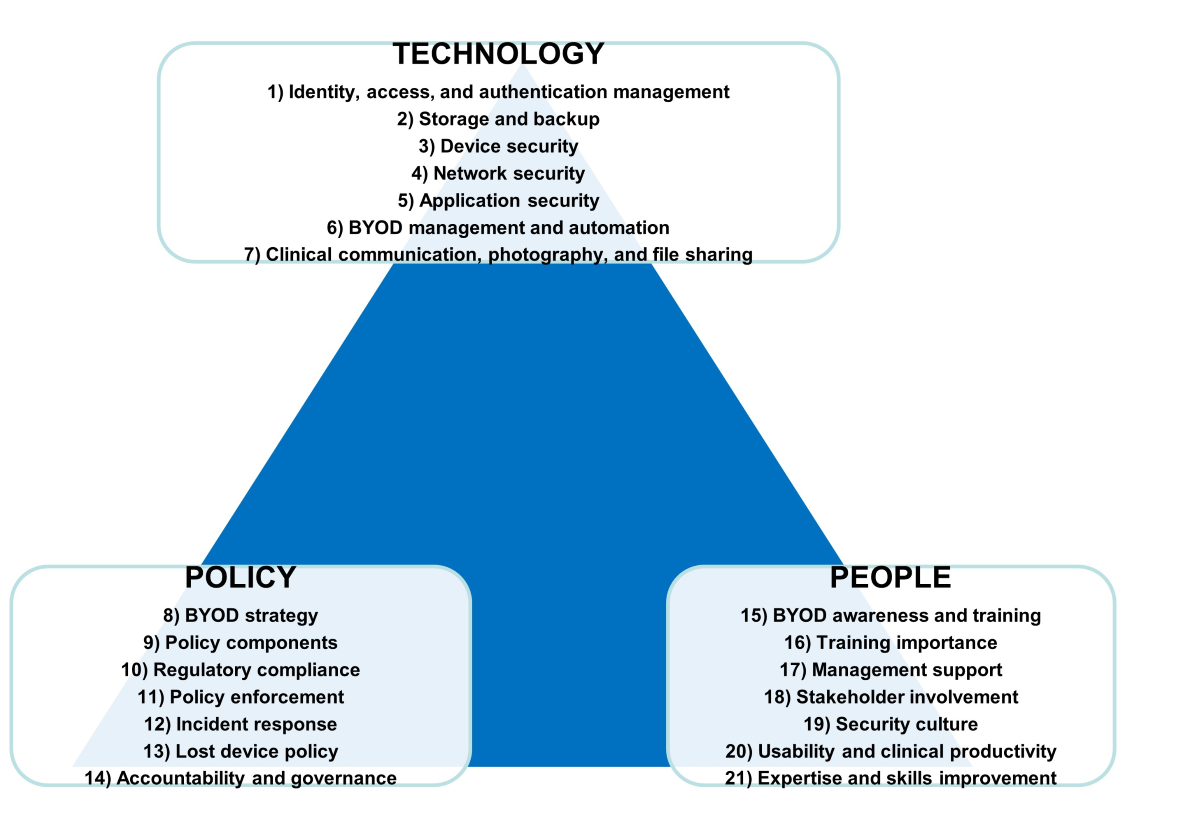
Hospital bring-your-own-device (hBYOD) security maturity model—dimensions and domains. BYOD: bring your own device.

Participant feedback led to both content and structural refinements of the maturity model. Content changes included clarifying technical terms, enhancing policy definitions, and tailoring guidance to BYOD use scenarios. Structurally, overlapping domains were consolidated for clarity and coherence. Detailed revisions, including before-and-after descriptions, are provided in Multimedia Appendix 4. Figure 3 illustrates the revised 21 domains across the 3 dimensions of the hBYOD security maturity model.

### Pilot Study: BYOD Security Maturity Assessment and Recommendations for Model Hospital

#### Maturity Assessment Survey Results

**Figure 4:**
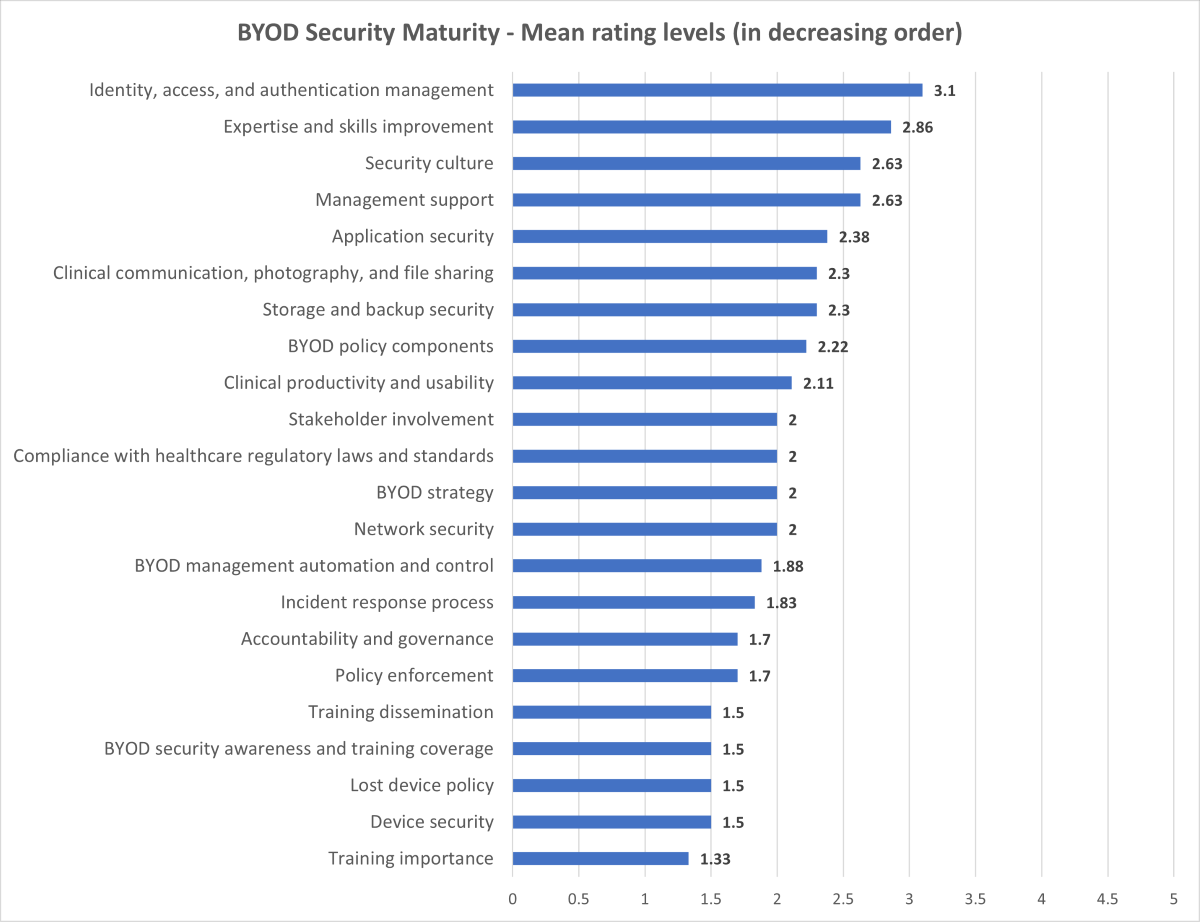
Bring-your-own-device (BYOD) security maturity assessment results for the model hospital.

The hospital BYOD maturity assessment evaluated security practices for the model hospital across the initially envisaged 22 domains, categorized into technology, policy, and people dimensions. The overall mean maturity level was 2.04, indicating foundational security practices. Technology scored an average of 2.21, policy scored an average of 1.85, and people scored an average of 2.07. Figure 4 provides a summary of the assessment results.

#### Workshop Findings and Recommendations

#### Overview

In the maturity assessment survey, participants ranked domains to identify priority areas. Given time constraints, only the top 2 ranked domains from each PPT dimension were discussed in the workshop. The following summarizes key challenges and solutions mapped to the maturity model recommendations:

#### Priority Domain 1 (Technology): Identity, Access, and Authentication Management

### Challenge 1: Cumbersome Authentication Requirements

Clinicians faced productivity challenges due to complex authentication processes, such as logging into multiple hospital applications, setting up 2-factor authentication, and managing regular BYOD updates. The varying levels of technology expertise among clinicians further exacerbated the issue, increasing dependency on IT support staff.

### Recommendations

The first recommendation was BYOD workflow analysis. Tailor authentication processes to align with clinical workflows, minimizing disruption (usability and clinical productivity: level 4). Define access levels based on roles and analyze data flows to ensure security without impacting productivity (IAM: level 4). The second recommendation was advanced IAM solutions. Invest in role-based access control, multifactor authentication, and SSO services to streamline IAM across device types and locations, enhancing efficiency and reducing IT overhead (IAM: level 4).

### Challenge 2: Managing BYOD Device Identities Across Multiple Health Services

Clinicians working across multiple health services encounter conflicts in BYOD requirements, making it impractical to implement device management solutions such as MDM.

### Recommendations

The first recommendation was *hospital-owned devices or device segregation.* Encourage using separate BYOD devices for different health services to simplify management. Alternatively, provide hospital-owned devices to staff working at multiple health services after following an approval process. Allow full administrative control over devices for IT administrators (device security: level 3). The second recommendation was centralized identity management**.** Implement state-level centralized identity systems to provide a unified authentication experience across multiple health services, ensuring compatibility and reducing complexity (IAM: level 4; BYOD management automation and control: level 4).

#### Priority Domain 2 (Technology): Clinical Communication, Photography, and File Sharing

### Challenge 1: Lack of a Sanctioned Platform for Clinical Communication

The absence of a formal strategy led to the use of multiple platforms for clinical communication, photography, and file sharing, with no consistency. Hospitals relied on tools such as Microsoft Teams, SharePoint, and OneDrive, while clinicians often used personal apps such as WhatsApp and Messenger for communication.

### Recommendations

The first recommendation was strategy and policy development. Define and formalize a comprehensive strategy outlining approved platforms, processes for obtaining patient consent, and guidelines on information sharing and separation of personal and hospital data on BYOD devices (clinical communication, photography, and file sharing: level 3). The second recommendation was stakeholder engagement. Ensure buy-in from clinicians and other stakeholders to promote adherence to the strategy, minimizing policy circumvention (stakeholder engagement: level 4).

### Challenge 2: Use of Unsecure Platforms

Unsecure platforms for communication and file-sharing posed risks, such as data leakage and unauthorized access, especially when patient data or photographs were stored on personal devices or cloud storage.

### Recommendations

The first recommendation was to formalize generic platforms. Restrict the use of unsecure personal apps and formalize hospital-approved platforms such as Teams, SharePoint, and OneDrive, leveraging their encryption and secure storage capabilities to reduce risks (clinical communication, photography, and file sharing: level 2). The second recommendation was to invest in dedicated platforms. Opt for secure clinical communication and file-sharing platforms tailored for hospital use. These platforms should integrate with clinical systems, support electronic patient consent for photography and data sharing, and improve usability, thereby enhancing clinical workflows (clinical communication, photography, and file sharing: level 4).

#### Priority Domain 3: BYOD Strategy

### Challenge 1: Lack of Strategic Direction

Workshop participants highlighted the absence of a clear strategic framework for BYOD security. Sociotechnical controls were implemented on an ad hoc basis, with critical aspects such as approved applications, device definitions, information exchange, and policy enforcement measures left undefined. Inconsistencies in related policies further compounded the issue, creating confusion among staff.

### Recommendations

The first recommendation was to develop a comprehensive strategy. Establish a formal BYOD strategy addressing both clinical and nonclinical aspects, with a strong focus on patient and staff data privacy and security (BYOD strategy: level 3). The second recommendation was to consolidate existing controls. Streamline and formalize existing sociotechnical controls to eliminate inconsistencies and conflicts in related policies (BYOD strategy: level 3). The third recommendation was effective communication. Disseminate the strategy through various channels to ensure staff understand and comply with BYOD security guidelines, fostering clarity, direction, and user buy-in (stakeholder involvement: level 3).

#### Priority Domain 4: Accountability and Governance

### Challenge 1: Undefined Roles and Responsibilities

Workshop participants highlighted a lack of clarity regarding BYOD-related roles and responsibilities, leading to confusion among staff. Clinicians were unsure about boundaries for BYOD use, such as when and where devices could be used (eg, within the hospital or after work hours) and for what purposes. In addition, there was insufficient guidance on actions required to ensure patient data privacy, responsible BYOD use, and compliance with legal and hospital policies.

### Recommendations

The first recommendation was to define and communicate roles: Clearly outline BYOD-related roles and responsibilities through user agreements or employment contracts (accountability and governance: level 3). The second recommendation was a BYOD governance framework. Implement a governance structure that specifies reporting lines and promotes accountability (accountability and governance: level 4). The third recommendation was policy alignment. Ensure these expectations are integrated into the hospital’s BYOD strategy to enhance compliance and improve user behavior (BYOD strategy: level 3).

### Challenge 2: Lack of Transparency

Staff expressed concerns about the rationale behind certain security measures, such as MDM, which they perceived as intrusive to personal privacy. This lack of transparency created mistrust, with staff uncertain about the implications of security requirements such as dual-factor authentication and device restrictions.

### Recommendations

The first recommendation was to enhance transparency. Clearly explain the rationale behind BYOD security measures, addressing staff concerns about personal privacy and productivity (security culture: level 3). The second recommendation was effective communication. Use the BYOD policy, user agreements, and training sessions to inform staff about privacy protections and hospital efforts to maintain productivity (BYOD awareness and training: level 3; stakeholder involvement: level 3). The third recommendation was to build trust. Foster confidence by demonstrating how personal data are safeguarded and ensuring staff understand the benefits of implemented security measures (accountability and governance: level 4; security culture: level 4).

#### Priority Domain 5: Stakeholder Involvement

### Challenge 1: Minimal Input From Clinical Stakeholders

Both clinical and IT management groups identified the lack of clinician input in BYOD policy and strategy development as a key issue. This gap often led to policies misaligned with clinicians’ workflow and productivity needs, resulting in limited buy-in. The compliance-driven focus of IT management, aimed at meeting state regulatory requirements, was seen as a contributing factor. Clinicians also raised concerns about meeting device requirements and the additional costs of using personal mobile data. On the IT side, engaging busy clinicians in strategy discussions was challenging, as cybersecurity often took a lower priority compared to their clinical responsibilities.

### Recommendations

The first recommendation was to involve senior clinical stakeholders. Engage senior informatics officers, such as chief medical informatics officers or chief nursing informatics officers, in BYOD policy development and decision-making. These leaders can act as bridges between clinicians and IT management (stakeholder involvement: level 3). The second recommendation was to facilitate regular engagement. Establish regular forums, such as monthly meetings, to discuss BYOD security issues, raise awareness, and gather clinician feedback (stakeholder involvement: level 4). The third recommendation was to promote cybersecurity awareness. Use informatics officers to educate clinicians on the importance of cybersecurity, integrating it into their roles to encourage active participation in related activities (stakeholder involvement: level 5).

#### Priority Domain 6: Security Culture

### Challenge 1: Convenience-First Environment

Participants noted that in a hospital setting, patient care often takes precedence, leading to a preference for convenience over data security. Clinicians frequently circumvent policies by using unsanctioned platforms, such as WhatsApp, for tasks such as sharing patient photos or clinical information.

### Recommendations

The first recommendation was user-friendly policies and tools. Minimize workflow disruption by ensuring BYOD tools and processes are intuitive and easy to use (usability and clinical productivity: level 4; security culture: level 3). The second recommendation was comprehensive training programs. Enhance staff awareness of BYOD security risks through interactive and tailored training methods, such as phishing simulations and role-specific modules (BYOD security awareness and training: level 4). The third recommendation was strategic awareness campaigns. Educate clinicians on the impact of policy circumvention to foster compliance and reduce reliance on insecure platforms (security culture: level 4).

### Challenge 2: Resistance to Change

Clinicians may resist BYOD security measures, citing fears of personal privacy intrusion or productivity losses. For example, concerns about device monitoring or objections to multifactor authentication and mandatory updates are common.

### Recommendations

The first recommendation was to change management programs. Develop systematic approaches to address resistance, incorporating strategies to reduce disruption and alleviate concerns (security culture: level 4). The second recommendation was the hospital’s commitment to cybersecurity. Ensure visible management support to emphasize the importance of cybersecurity (management support: level 4). The third recommendation was to engage change champions. Use influential clinicians to advocate for security measures, explain their rationale, and address concerns about privacy and workflow impacts within their departments (security culture: level 5).

The workshop structure and screenshots of brainstorming notes from the MURAL worksheets used by the clinical and IT management groups are provided in Multimedia Appendix 5.

## Discussion

### Principal Findings

This study introduces a sociotechnical maturity model that offers a holistic approach to addressing hospital BYOD security risks. By combining technical controls with organizational policies and human factors, the model fills existing gaps in the literature by integrating technical controls with organizational policies and human factors. It uniquely defines maturity levels across 21 specific domains, making it modular and providing detailed guidance throughout all areas of BYOD security. In addition, encompassing the key dimensions of PPT, it offers a comprehensive framework that spans the entire BYOD life cycle within hospitals. This aligns with the recommendations of several researchers who highlighted the importance of integrating sociotechnical elements into information security management [22,51-53]. Furthermore, a prominent feature of the maturity model is its accommodation of unique health care sociotechnical factors. Domains, such as clinical communication and collaboration, clinical productivity and usability, and compliance with health care regulatory laws and standards, better represent the complexity of cybersecurity in health care settings, especially concerning BYOD environments. This focus is supported by McLeod and Dolezel [54], who emphasized the critical role of regulatory compliance and user behavior in health care cybersecurity. The model also contributes theoretically by operationalizing abstract socio-organizational factors, such as security culture, into discrete, assessable maturity levels. This allows hospitals to systematically measure and improve human-centered dimensions of security previously overlooked in dominant frameworks.

This model also directly responds to several limitations identified in cybersecurity maturity literature. For instance, Büyüközkan and Güler [33] noted the lack of industry-specific models and insufficient emphasis on human and organizational factors. While their model is broad and literature based, it does not include detailed, structured maturity levels or any health care–specific focus. Bitzer et al [35] present a sociotechnical maturity model with tangible evaluation metrics, but it is limited to incident response and does not extend across the broader health care cybersecurity landscape. Similarly, Smyrlis et al [36] offer a health care cybersecurity risk assessment model that is entirely technical and lacks both maturity level structuring and attention to human or organizational dimensions. AlDaajeh and Alrabaee [34] center their work on national-level cybersecurity capabilities rather than institutional or clinical implementation. In contrast, our model provides a modular, multidimensional, and health care–specific approach that can be practically applied within hospitals, offering clearly defined maturity levels across technology, policy, and people dimensions to guide structured, real-world improvement in BYOD security. In addition, the structured and modular design of the model facilitates alignment with internationally recognized cybersecurity frameworks, such as ISO/IEC 27001 and the National Institute of Standards and Technology Cybersecurity Framework [55]. By covering key areas, including governance, access control, incident response, and compliance monitoring, the model enables translation of these high-level standards into context-specific, actionable practices. It supports hospitals in operationalizing global best practices through enforceable policies, measurable progress indicators, and a commitment to continuous improvement, all while addressing the distinctive sociotechnical challenges posed by BYOD in health care environments.

The model was developed through MMAR, which synergistically integrated perspectives from both IT management and clinical users. MMAR facilitated the consolidation and critical analysis of findings from multiple empirical studies, enabling the refinement of the preliminary framework and the mapping of recommendations to formulate a comprehensive maturity model that ultimately provides a structured road map for enhancing BYOD security capabilities within hospitals. Being incremental and iterative in nature, MMAR can allow successive refinements to the maturity model based on evolving insights by incorporating emerging cybersecurity threats, advancements in technology, and evolving hospital security needs. As demonstrated in this study, stakeholder engagement through IT and clinical representatives played a crucial role in refining the maturity model. Therefore, methodologically, this study contributes to cybersecurity maturity model development by applying MMAR, enabling co-creation with end users. This iterative, participatory process grounds the model in real-world hospital contexts and contributes to theory by demonstrating the value of embedded stakeholder engagement in model formulation.

In terms of model updating, a cyclical evaluation process can be applied for future updates to the model through a structured step-by-step approach. First, periodic reassessments should be conducted through a regular review of industry best practices, emerging security frameworks, and hospital-wide BYOD security evaluations, including surveys and compliance audits, to identify gaps and risks. Second, real-world case studies can be integrated by analyzing security incidents, policy effectiveness, and user compliance trends, ensuring the model reflects actual BYOD security challenges in hospitals. Third, expert consultations with cybersecurity professionals, health care IT leaders, and policy makers can help incorporate regulatory updates, advancements in security technologies, and evolving best practices. Fourth, refining domain definitions and maturity levels based on the best practices review, stakeholder feedback, and empirical findings will ensure that the model accurately represents new threats, technologies, and governance strategies. Finally, validation and implementation should occur through structured feedback loops, pilot testing across diverse health care settings, and continuous monitoring, enabling iterative refinements that enhance the model’s adaptability and long-term applicability in hospital environments. This stepwise approach, enabled by the incremental nature of MMAR, ensures that the BYOD security maturity model remains dynamic, evidence based, and responsive to the evolving cybersecurity landscape in health care.

The technology dimension covers various BYOD security technical controls and practices across domains such as IAM, storage and backup security, device security, network security, application security, and clinical communication, photography, and file sharing. The lower levels reflect basic practices, such as single-factor authentication systems, mixing of hospital and personal data on BYOD devices, use of basic firewalls, no restriction or control over BYOD use, and use of personal and unsecure apps for clinical communication and collaboration. Such practices may lead to a higher risk of BYOD-related data breaches, as they suggest a complete dependence of BYOD security on user action due to a lack of control over BYOD-related user activities. As we move to higher levels, advanced technical controls, such as enterprise IAM solutions, federated and unified SSO, private cloud for hospital data storage and backup, unified end point protection to manage BYOD devices, network segmentation and visualization, software-defined networking, and a dedicated secure platform for clinical communication integrated with hospital clinical systems, are stipulated. These features minimize the risk of hospital data leakage, restrict unsecure behavior, improve clinical workflow, and also enable better management and control over hospital BYOD use. Such practices align with the topmost or “optimized” level of prominent information security maturity models [28].

The policy dimension focuses on hospital BYOD security strategy, governance, and compliance factors. Lower levels specify policy practices such as a lack of a formal BYOD governance structure, ad hoc and inconsistent policies on BYOD security, a slow incident management process, no or unchecked policy enforcement, and poor compliance to relevant regulations or standards. These practices indicate a lack of direction and guidance on BYOD security, which can result in poor management of BYOD security incidents and greater chances of breach of relevant legislation or regulations. Higher or more advanced levels are characterized by a well-defined, formalized, and comprehensive BYOD strategy and related policies, which are regularly updated in view of changes to the threat, business, and legal landscape. Real-time, automated, and advanced compliance measurement and incident management processes are also key features of higher BYOD policy maturity levels. Therefore, hospitals at higher BYOD security maturity levels are better guided and equipped to prevent, detect, and respond to BYOD security incidents. Such a proactive approach, incorporating technology-enabled solutions for real-time compliance monitoring, aligns with prominent adaptive security management frameworks emphasizing the importance of continuously enhancing security practices by leveraging real-time data to promptly detect security incidents, monitor compliance, and adapt policies as needed [56,57].

Finally, the people dimension addresses the “human” element of hospital BYOD security and therefore consists of domains such as BYOD security awareness and training, stakeholder involvement, security culture, and clinical productivity and usability. The lower maturity levels of the “people” dimension indicate a reactive security culture where there is no or low commitment from hospital IT management and clinicians alike to improve BYOD security practices. It is characterized by minimal stakeholder input on BYOD security management, a restrictive BYOD environment, a lack of BYOD security training, and minimal skills or low expertise to manage BYOD security issues. Such measures may lead to unsafe security behavior among hospital staff, circumvention of policies, and a tension between clinical and IT departments, ultimately increasing chances of BYOD security breaches. As far as higher maturity levels are concerned, they indicate a proactive security culture, where cybersecurity is seen as a top priority by management and staff alike. Therefore, hospitals at an advanced state of BYOD security handle cybersecurity affairs autonomously and may implement measures such as dedicated BYOD security training tailor-made to clinical specialty, ongoing management support, and a regular stakeholder consultative process for BYOD security strategy or policy development. Eventually, these practices can improve staff commitment and knowledge of BYOD security while also ensuring that BYOD use is made productive, in addition to being secure. A balanced approach that effectively balances productivity with security can lead to a positive security culture, thereby improving compliance and reducing risks [58-60].

The pilot implementation demonstrated how the hospital BYOD security framework can be applied to assess current practices and codevelop tailored recommendations. The maturity survey identified key gaps, while the workshop enabled stakeholder-driven discussions to guide progress from the “as-is” to the “to-be” state. This participatory approach proved valuable in addressing BYOD security challenges that are particularly important in complex and dynamic environments such as hospitals [61].

### Change Management Considerations

While the maturity model provides a structured approach to hospital BYOD security, its implementation presents several challenges that hospitals must consider. Resource requirements vary depending on the hospital’s current BYOD security posture, as advancing to higher maturity levels may require investment in MDM solutions, end point security tools, secure authentication systems, staff training programs, and dedicated cybersecurity personnel. Change management strategies are essential to ensure smooth adoption, particularly when introducing stricter BYOD policies, access controls, or security monitoring tools that may impact clinical workflows. Engaging stakeholders, including IT teams, clinicians, and hospital administrators, through structured communication, tailored training, and phased rollouts can minimize disruption and improve adherence. Potential resistance from stakeholders, especially clinical staff, must be addressed by demonstrating that security measures do not hinder usability or productivity. Establishing leadership buy-in and appointing clinical and IT champions can further support organizational alignment and reduce friction during implementation. Furthermore, hospitals should ensure that BYOD security policies are clinically feasible, providing secure yet user-friendly alternatives (eg, approved hospital communication apps instead of WhatsApp) to prevent the circumvention of policies through shadow IT. Integration with existing systems is another key consideration, as BYOD security measures must be compatible with EMRs, secure messaging platforms, hospital networks, and cloud-based collaboration tools without disrupting access to critical clinical information.

### Significance

The maturity model serves as a valuable tool for assessing the current state of BYOD security management within a hospital, leading to a better understanding of strengths, weaknesses, and gaps in the existing security posture. It facilitates structured improvement toward a higher maturity level. Both internal and external technology or security auditors can use the model. Internal auditors, such as the hospital’s IT or cybersecurity practitioners, can periodically assess BYOD security to set new improvement goals. Hospitals can also engage specialized auditors to carry out the assessment process.

Hospitals can follow a systematic approach to improve their BYOD security posture using the maturity model (Textbox 1).

In addition, government agencies, such as health departments, can use the maturity model as an audit tool to assess whether a hospital’s BYOD security aligns with established standards.

**Improving bring your own device (BYOD) security posture using the hospital BYOD security maturity model.**

1. Understanding the current maturity state: conduct a BYOD security assessment across the 21 domains. This step establishes the baseline or “as-is” state, crucial for understanding the current BYOD security position.
2. Identifying priority domains: determine critical areas or domains of BYOD security that require attention. Prioritization is essential because hospitals have finite resources and may not be able to improve all domains simultaneously. Improvement should be carried out step by step, addressing priority areas first. Involving all relevant stakeholders, especially technical and clinical personnel, ensures that user requirements are considered.
3. Setting improvement goals: develop achievable and measurable improvement goals based on the progressive maturity levels specified by the model. Identify which maturity level to aim for in each priority domain, considering hospitals’ unique needs, constraints, and regulatory requirements. Improvement goals should be clearly defined using quantifiable benchmarks or key performance indicators, such as increasing SSO adoption rates, training completion percentages, or MDM enrollment levels, to track meaningful progress. Goals should be feasible, with a well-thought-out plan that gains consensus among all relevant stakeholders within the hospital.
4. Measuring progress: continuously monitor the progress of set goals against measurable, time-bound metrics set in stage 3 to systematically measure progress and ensure structured, data-driven maturity advancement. Verify that all actions required to advance to the next maturity level have been completed, providing concrete evidence of progress.
5. Revisiting maturity improvement: recognize that BYOD security management is an ongoing process requiring regular cycles of improvement. Establish a reasonable time frame to revisit and enhance controls and processes to counter evolving and complex BYOD security threats.

### Limitations

The utility of the proposed hospital BYOD security maturity model has not been extensively tested across diverse hospital environments. Although significant effort was invested to enhance the model’s accuracy by mapping maturity levels to recommendations derived from empirical studies and aligning them with industry standards, the model’s practical effectiveness remains to be fully validated. Feedback from study participants was incorporated to refine the model further; however, additional validation and evaluation studies are required in various hospital contexts to improve its accuracy and efficacy. In addition, the rapidly changing nature of cybersecurity threats and technologies means that the model may require continuous updates to remain relevant. New types of security risks, emerging technologies, and changes in legal requirements could necessitate revisions to the model’s domains and maturity levels. Another consideration is the approach to maturity assessment; while averaging assessors’ ratings provides an overview, it can obscure key issues in the maturity assessment process. A structured consensus-building method, such as the Delphi approach used in the development of the Essentials of Cybersecurity in Healthcare Organizations framework [62], could enhance assessment reliability by integrating expert opinions systematically. Future research should focus on implementing such consensus-driven assessment methods alongside extensive validation studies in diverse hospital settings to refine the model, enhance its generalizability, and ensure it remains a practical tool for improving BYOD security in the ever-evolving landscape of health care cybersecurity. In addition, future validation studies may provide further insights into refining behavioral assessment methodologies, potentially incorporating more direct behavioral metrics where needed to better evaluate how security behaviors are enacted in practice.

Equal representation of both clinical and technical stakeholders was initially planned; however, due to limited availability, only 3 clinical representatives were able to participate in the workshop. Nonetheless, these clinicians had consulted with their broader teams beforehand and conveyed shared concerns and perspectives during the assessment. This imbalance in representation may have introduced a bias toward technical perspectives in the model’s pilot results. Future research should aim for a more balanced stakeholder composition and a larger, more diverse sample to ensure broader applicability and validation.

### Conclusions

This study developed a sociotechnical BYOD security maturity model tailored for hospitals, addressing gaps in existing cybersecurity models. By integrating technical controls, policies, and human factors, the model provides a holistic approach to managing BYOD security risks. The pilot implementation demonstrated how hospitals can use the model to create a structured, modular road map for enhancing security while balancing the unique sociotechnical complexities of health care.

## Appendix

Multimedia Appendix 1 Hospital bring-your-own-device security framework recommendations and evidence.

Multimedia Appendix 2 Final hospital bring-your-own-device security maturity model description.

Multimedia Appendix 3 Maturity Assessment Survey.

Multimedia Appendix 4 Maturity model revisions.

Multimedia Appendix 5 Workshop structure and brainstorming notes.

## Abbreviations

BYOD: bring your own device
EMR: electronic medical record
hBYOD: hospital bring your own device
IAM: identity and access management
MAM: mobile application management
MDM: mobile device management
MMAR: mixed methods action research
PPT: people, process, and technology
SSO: single sign-on
