# Supplementary material for: A Sociotechnical Approach to Bring-Your-Own-Device Security in Hospitals: Development and Pilot Testing of a Maturity Model Using Mixed Methods Action Research
Source: JMIR Hum Factors. 2025 Aug 13;12:e71912. doi: 10.2196/71912 (PMC12391842; doi:10.2196/71912)
Supplement: Multimedia Appendix 1 [file humanfactors_v12i1e71912_app1.docx]

## Multimedia Appendix 1: hBYOD SECURITY FRAMEWORK: RECOMMENDATIONS AND EVIDENCE

**STAGE 1: Plan**

| **RECOMMENDATION** | **EVIDENCE/EXAMPLES** |
| --- | --- |
| **R1.01: A dedicated, clear, and comprehensive BYOD policy addressing all clinical and non-clinical areas relevant to BYOD security should be developed** | **Study 1A:** Hospitals which had a BYOD policy had a higher average BYOD security maturity rating (3.37 out of 5) as compared to those who didn’t (1.86) as well as the overall average (2.94). |
|  | **Study 1B:** Table on policy components  *“for me, it's very much about getting the (BYOD) policies and procedures right, up front...* *you need to be refreshed, retrained, keep up to date with your policies”* P2 |
|  | **Study 2A:** Higher mean knowledge scores across all BYOD security domains asked in the survey for participants supplied with a policy  Mean knowledge score for participants supplied with a policy higher (3.11) as compared to those for whom policy was not supplied (2.11). |
|  | **Study 2B:** *“…Well, they're (policies) reasonably consistent across the hospitals. I think by agreeing to do something, you certainly become aware that you have to abide by those. And I'm sure there's a bit more of an uptake or there's more compliance by having to ask people to agree on the terms. And obviously then it also acts as a sort of binding thing…”* Doctor5  *“I think that (policy) really helps in as it’s a form of recognition of your responsibilities.”* AHP2  *“..So if they (policies) were custom made to a particular area or particular speciality, then they would have better impact…”* Doctor1 |
| **R1.02: Policy should be in alignment with hospital’s objectives, vision, needs, and resources available** | **Study 1A:** Generally, resource constrained hospitals such as smaller or regional hospitals were found to have lesser number of socio-technical security controls for BYOD security.  A lower mean maturity rating for regional hospitals was also seen (2.77) as compared to the overall average (2.94). |
|  | **Study 1B:**  *“our BYOD policy is quite immature. I'd say 100% of the organisation wouldn't even know what it is. They'd probably even struggle to find it. So it's a fairly haphazard policy. That's not due to lack of commitment. That's just due to lack of priority, I suppose, within the business.”* P7  *“if we did have funding in that area, we would probably enforce more mandatory controls, given that my staff are happy to provide their own devices. Some products have limitations in terms of user experience, so they (staff) would prefer an enhanced user experience, but with our current technology budget, we really can't provide them that. Hence, the reason our BYOD policy hasn't really progressed much in the last five years..”* P7 |
|  | **Study 2A:** No relevant evidence found |
|  | **Study 2B: “***My department is very much focused on funding and all that monetary sort of side. That would be a huge barrier to see whether just simply integrating something into our personal devices would yield a benefit in the long term or not. Not even the long term, but short term as well, financially…”* AHP1  *“Health services have fixed revenue streams for them to come in, they can only spend a certain amount. Otherwise, they go into the red. So unless money is somehow attached to these things, there's no driver for them happening unless the CEO understands that and sees it as something worthwhile for the health service, and prioritizes that over something else.”* P3 |
| **R1.03: Policy should recognise the need to maintain clinical productivity and balance between security and usability must be maintained** | **Study 1A:** Improvement in clinical workflow and productivity was ranked as the topmost reason for allowing BYOD.  Most participants (90%) also believed that clinical workflow integration is either a ‘very important’ or ‘important’ step in BYOD security management. |
|  | **Study 1B:** *“you can implement as many technical controls as you like, but humans being humans, in any system there's ways to get around the system, and if we don't provide a reliable service in terms of providing an instant messaging platform to meet the needs of the clinicians, then they'll go and do it by other means and at the simplest thing just go and ring each other up, which is probably more secure than sharing clinical information on an insecure platform, like WhatsApp or so forth.”* P7  *“For BYOD, I think you've got to do those conversations about the balance of personal rights and organisational rights and private patient rights. That takes a little bit longer to put in place and I think we will push into that and I think we will formalise it and put more rigor round it.”* P2  *“The clinician demands that I should be able to do whatever I want on my mobile device and you should deliver it to me for ease of use in whatever way I want. At a very simplistic level that makes perfect sense, but from a technology level, and from a security level, and from a patient privacy and a privacy act level, that just is so full of challenge. The clinician is not interested in that, but rather to actually deliver something that is useful that makes sense at the end of the day.”* P5 |
|  | **Study 2A:** 37% surveyed clinicians believed that the hospital BYOD security processes hampered their clinical productivity in some way, 28% gave a neutral rating, 15% rated “minimal” and 20 % rated “no impact at all”. Higher impact ratings were seen for doctors. |
|  | **Study 2B:** *“..It (policy) really needs to fit in with the workflow. Of what I've seen in my department, any minor annoyances will really put off the clinician!..”* AHP1  ***“****I think the policy should for a start acknowledge that use of your own device is appropriate...Good policy that understands the (clinical) context would be excellent..”* Doctor4  **“***I don't think everybody actually obeys the rules, per se. I think a lot of the senior nurses think they've been there for much longer, so they take shortcuts and actually do things that they're probably not supposed to do… Just the heavy load, the business, making sure that things are done on time is very important (to them)…”* Nurse1  *“..they (clinicians) have a focus that their minds on how to produce the best outcomes for their patients. And so privacy might not necessarily be the first thing that they would consider, their primary objective is treatment or how can I make things easier for my patient..”* AHP3 |
| **R1.04: Policy should be well-structured policy, brief, and easy to understand** | **Study 1A:** No relevant evidence found |
|  | **Study 1B:** *“policy has been a little bit clunky and hit-and-miss…. there's not a lot of people who are bringing out the policy and shoving it under people's noses. It's sitting there in the background”* P3 |
|  | **Study 2A:** No relevant evidence found |
|  | **Study 2B:** *“..It's (Policy) quite long…Maybe having smaller sections that you can click on, like divide them into subsections. And if you need that one, that's what you're going to click on instead of having one 10-page rule.”* Nurse4  *“Putting it (policy) in plain, simple English and having someone possibly to talk it through with them and reassuring them…”* AHP1  *“I think it's good for people to understand the process…the layman rationale is lacking (in policy”* AHP3 |
| **R1.05: A formal governance structure should be established with a clear ownership of the BYOD program and roles and responsibilities of all stakeholders defined** | **Study 1A:** Hospitals where a dedicated cybersecurity officer was responsible for BYOD security management had a higher maturity rating (3.065) as compared to hospitals which didn’t (2.815) and the overall average (2.94). |
|  | **Study 1B: “***From a management point of view, it is (important) having the right governance around BYOD security management, It’s still challenging. So you need the right governance…You need to be ensuring that you don't have any data leakage, that's probably our biggest issue. Putting in the necessary processes and tools around that...”* P2  *“Lots of people are involved in that (policy/strategy development). So there would be a business owner for the particular function, so we have a data governance and security director and if there was a new policy rollout they would start it in that data area. She would initiate the changes and they would go round to relevant stakeholders for people to comment and input. Then they take it to the governance and security committee, for instance. And from there once that's ratified, we publish them.”* P2  *“it's (strategy/policy) very much the responsibility of our IT department, which we call digital technology services… it's been something that's really just been IT and executive...”* P1 |
|  | **Study 2A:** No relevant evidence found |
|  | **Study 2B: *“****A policy needs to be… someone needs to own it.”* AHP4  *“I think that (policy) really helps in as its a form of recognition of your responsibilities.”* AHP2  *“there's still some things on there that I never knew that I could access, or there's still some grey areas of what we can and can't do, or can and can't search when we're on there (in hospital). Wish it wasn’t complicated..”* Nurse4 |
| **R1.06: Clear and transparent reporting and communication lines between different departments, domains, or stakeholders should be established** | **Study 1A:** No relevant evidence found |
|  | **Study 1B:** *“I think my impression is that generally there's very little clinical engagement. There's just not necessarily an appreciation of the impact that it has on clinicians. And I think the IT people just sort of see it as an IT problem, so why would I bother type of thing? There is a bit of a lack of dialogue.”* P3  “*There hasn't been clinical engagement where there should be, as the people that are going to be using it should give their perspective.”* P2 |
|  | **Study 2A:** No relevant evidence found |
|  | **Study 2B:** *“So clinicians are not necessarily sort of IT or tech savvy. They come and they want to treat patients. And when the IT processes get in the way, they get frustrated quickly. And when you are trying to contact IT and IT isn't understanding what you're asking them, because they don't speak the language that you speak. Like the problem is there, yes and IT can fix it, but the way that you're presenting it to them is not in a way that they understand it. There's some tension there…”* AHP4 |
| **R1.07: All designed technical processes, controls or architecture related to BYOD security should be secure, minimally invasive as well as user friendly** | **Study 1A:** Most participants (90%) believed that clinical workflow integration is either a ‘very important’ or ‘important’ step in BYOD security management. |
|  | **Study 1B:** *“what you're aiming for generally is, that you just have one platform and a single sign on. At the moment most of us would use at least three different systems just for patient stuff. We're still in a bit of a hybrid thing. It's a bad look when you put in a username and password and then the next window that comes up is another username and password. From a user perspective, they think, "Well, these people don't know what they're doing," it's bad plus it's time consuming as well (for them).”* P3  *I think the challenge is if you've got a user who wants to bring a device into the work environment then you have got to make sure that they are willing to provide you with the ability to control that device should it go missing or fall into the wrong hands, which doesn’t happen.”* P5 |
|  | **Study 2A:** Only 2% participants were willing to provide full administrative privileges to hospital authorities for BYOD security purposes, as compared to 31% (selective control only over hospital data or services), 14% (neutral), 17% (minimally willing) and 36% (not at all willing). |
|  | **Study 2B:** *“I think that's the major factor because I think that the way that it's (security processes) designed is going to affect the way that it's used. I think that there will be people who can always find an unsafe way or unorthodox way of using these systems. And you can't stop that entirely, but you can make it very hard for people to do that. And that then means that the person who doesn't really care is trying to use the easiest way of doing things. If the easiest way of doing things the correct way, they will use the correct way..."* Doctor5  *“...Never, I wouldn't trust them (hospital management) within a million miles. They should never be allowed to have any sort of input or control over my device...So no, they are not getting anywhere near my device...If they wanted to build an app that could interface with their system in an efficient manner, then I'm comfortable with it. But to have anything that would have any ability to control or influence my device's function, never...”* Doctor4  *“…because at the end of the day, we are doing it (BYOD use) on our own behalf, and we're not protected from hospitals. It sort of puts the responsibility on the clinician when they're saving things on the device and if it goes wrong, they feel they are responsible and we don't get any special malwares or anti-virus from the hospital, so we had to push it and we had to keep our system updated. So there's a little bit of additional cost. If your device is old or has been outdated, it does become harder to use these kinds of facilities. You need to keep your system updated. And some people, I know they don't like to update their phone, but if you want to use your personal device, you are supposed to. I have been in occasions where my remote access was blocked because I hadn't updated my phone yet. That's a big challenge. If you do not want to do that, then you sort of lose access. …"* Doctor1 |
| **R1.08: Compliance to all relevant laws, regulations, and standards associated with BYOD security should be maintained and clinicians should be advised on how to achieve it in a BYOD environment** | **Study 1A:** Compliance with healthcare data privacy laws was rated as the topmost challenge related to BYOD security |
|  | **Study 1B:** *“the problem is that we're also not compliant. That, I think, is the work that needs to be done over the next couple of years, through providing alternatives, and then becoming a little bit more strategic and consistent with messaging, and auditing, and sanctions that are placed, which I think is the obligation.”* P3  *“The procedures and the technology have lagged and still are. I see that we'll have to do some rapid catching up. In terms of the controls, the 72 controls.. the audit feels like an overhead… it probably hasn't been great and it would be better if that was a bit more straightforward. 72 controls, it's a lot. I kind of like the “eight must do’s” than what the government's got. When you bring it down to five or six and then you can actually step down from that into a more granular level, it's a little easier to explain to people. I think the 72 controls could be done a little bit better so they're a bit more manageable.”* P2  **“***. We are much more decentralised, and everyone can follow their own what they think is best practice which sometimes makes it harder to manage if you're working across multiple health services or working with other health services. So yeah. I guess some standard practices would help and that government's in a position to influence that.”* P2 |
|  | **Study 2A:** Low mean knowledge was found among participants about maintaining compliance to relevant laws or legislations in a BYOD environment |
|  | **Study 2B: *“****I'm not fully up to date with the current laws and regulations… That's the area, which again, I say is not properly covered by the hospital IT. They sort of tell you that these are the policies, but no one really enforces them or checks. And there's no compliance checks…And if that can be done a bit more rigorously, I think people may understand laws and regulations better.”* Doctor1  *“..So there's a few concerns. One is for patient information, obviously ensuring that its firstly complying with patient privacy principles, which I'm not certain that I am at the moment completely…”* Doctor5 |
| **R1.09: All relevant clinical and non-clinical stakeholders should be involved in BYOD policy/ strategy development and decision-making** | **Study 1A:** No relevant evidence found |
|  | **Study 1B:** *“Where IT comes in and pushes a solution on the organisation and there's no stakeholder engagement, it's never going to be successful. If the clinicians came to us and said, "This is what we want," or "This is the problem we have. What are the options and what's your recommendation?" And there's buy-in from the clinical part of the business' stakeholders, with that clinical sponsorship, then it's more likely to be a success.”* P7  *“I think my impression is that generally there's very little clinical engagement in these things. There's just not necessarily an appreciation of the impact that it has on clinicians. And I think the IT people just sort of see it as an IT problem, so why would I bother type of thing? There is a bit of a lack of dialogue.”* P3 |
|  | **Study 2A:** No relevant evidence found |
|  | **Study 2B:** *“..Respect the actions of the clinicians. Don't demean us because we are doing stuff that they don't necessarily approve of. Actually, respect the fact that we are doing that for good and valid reasons, and work with clinicians to improve it, actually have a partnership culture...*” Doctor4 |
| **R1.10: Hybrid roles such as clinical informatics specialists should be used in BYOD strategy or decision-making** | **Study 1A:** No relevant evidence found |
|  | **Study 1B:** *“I think the whole concept of the position of chief medical informatics officer, chief nursing, or chief clinical informatics officer, is that the clinical person gets involved and provides meaningful input into policy, which would have probably been a bit difficult before. I think in the past, before we develop these hybrid mixed roles, the clinical people just did not understand yet the other side of it. There hasn't been clinical engagement where there should be, as the people that are going to be using it should give their perspective.”* P3 |
|  | **Study 2A:** No relevant evidence found |
|  | **Study 2B:** *“..there needs to be a conduit between clinicians and IT or administrators… this is another reason why they (hospitals) need more of health Informatics”* AHP3 |
| **R1.11: Cybersecurity should be made a hospital-wide priority and therefore full management support towards all cybersecurity initiatives should be provided** | **Study 1A:** No relevant evidence found |
|  | **Study 1B:** *“you've got the clinicians on one side and you've got the IT people on the other. So unless there's somebody who sits above everybody, who says exactly what you've just said and just tells them to make it (support to BYOD security initiatives) happen, then it does happen. Because we are very much on the same level as the IT people, like the clinicians on the shop floor, we're not the IT people's boss in any way. So it depends on that vision and understanding from overarching management, and really the only people who oversee both groups, it's really the CEO.”* P3  *“if we did have funding in that area, we would probably enforce more mandatory controls, given that my staff are happy to provide their own devices. Some products have limitations in terms of user experience, so they (staff) would prefer an enhanced user experience, but with our current technology budget, we really can't provide them that. Hence, the reason our BYOD policy hasn't really progressed much in the last five years. In public health, the funding is never what it needs to be. And people's expectations of technology are largely informed by their experiences with banks and so forth, where the budgets are four times ours in terms of ICT spend. So, we can never meet the expectations of the doctors, especially the younger doctors, and they're frustrated by that and we are frustrated by it as well.”* P7  *“Challenge I think is always to try to get funding to implement all these technologies. They are not cheap and everything's in the half a million-dollar, million-dollar mark… that's one of our main issues.”* P6 |
|  | **Study 2A:** No relevant evidence found |
|  | **Study 2B:** *“..A cultural barrier, especially in my department, is whether implementing or getting a vendor to implement an application into our personal devices yields a net benefit or not. My department is very much focused on funding and all that monetary sort of side. That would be a huge barrier to see whether just simply integrating something into our personal devices would yield a benefit in the long term or not. Not even the long term, but short term as well, financially…”* AHP1  *“..you also need to be able to support staff, support clinicians, but we know that in healthcare funding is always a big issue…”* AHP3 |
| **R1.12: A pro-active security culture, where both management and staff show deep commitment towards cybersecurity constantly should be established** | **Study 1A:** No relevant evidence found |
|  | **Study 1B:** *“The culture at an organisation is everything…”* P7  *“I think cyber issues tend to be handled more retrospectively then proactively at the moment because I think we're still relatively immature in that space. I think any situations where we've had to deal with potential breaches of patient privacy, they've come as a result of an investigation, as opposed to putting into place framework or restrictions ahead or proactively”* P5  *“It's up to the executives at each organisation to mandate training. As yet, we've been unsuccessful in getting that. So how we convince them, my obligation as a information security professional is just to identify what the risks are and how to address those risks or reduce those risks, and then it's a business decision whether there's an appetite to proceed… If the cybersecurity risk is taken seriously and promoted by the executive of the organisation, then the organisation takes it seriously.”* P6 |
|  | **Study 2A:** No relevant evidence found |
|  | **Study 2B: *“..****the biggest challenge I think is always certainly cultural.”* Doctor5  ***“****So I guess unfortunately hospital systems, change our behaviors by the outcomes…If I would know that someone's information was accidentally leaked out from their phone then I would be more careful or think twice before doing it.(Example of reactive actions)”* Doctor6  *“Some people are more keen and more proactive. Some people have better IT skills for them it's easy, but there is a lot of heterogeneity among the clinicians and if they're not provided adequate support, I think they are the ones who are at risk.”* Doctor1 |
| **R1.13: A comprehensive BYOD security awareness and education program for staff should be developed** | **Study 1A:** No relevant evidence found |
|  | **Study 1B:** *“It (training) is actually good because it keeps all the employees informed and up to date and it sets expectations and understanding of what is right. I actually think that regular training, appropriate to your role, with a refresh and a sign off each year that you've done it and you are up to date actually works.”*  P2  *“I think the challenge is to keep the workforce not just engaged, but well informed of what is an acceptable way of operating and what's not. And user education, I believe, or constant user education, I believe is a challenge, because we learn something, and then if we don't use it, we forget.”* P1 |
|  | **Study 2A:** Participants provided with BYOD security training by their hospitals had higher mean knowledge scores across all BYOD security domains or areas as compared to hospitals which didn’t.  Overall mean knowledge score for participants provided with a BYOD security training was higher (3.33) as compared to those to whom training was not provided (2.63). |
|  | **Study 2B:** *“I think it's a very important part before giving people access to these kinds of apps, people should know what they are encountering and how to make full use of it, and also be safe. And at the moment I feel this is the area which needs to be worked on more. I think lack of training is an important one of the challenges, because we don't get trained about challenges.”* Doctor1  *“Yeah. It (Training) made things easier, because we were using a very different system.”* Nurse3  *“The first time that I received training, it helped me to understand my obligations, learn, know and a bit more about the acts and legislation”* Doctor5  *“..having a clear training when you are using your own devices to ensure that everybody's got the tools on their device and knows how to use them before they need is important, because if the first time you're having to sort this out is when you actually need to use that tool, then it's not going to work for most people.”* Doctor4  *“...It (training) was more around some principles and some practical information. But not very comprehensive all the time. I was going to say a lot of it's been historically around the use of social media and things. So I do recall sometimes there being specific training on what's appropriate use of social media and sharing a patient interaction. Not so much around no use for personal devices for legitimate work purposes essentially...”* Doctor5 |
| **R1.14: Strategic, practical training tailor-made to clinical specialty should be provided** | **Study 1A:** No relevant evidence found |
|  | **Study 1B:** *“We do have a lot of training that people have to do already, and it's quite a burden. The more you have then the more diluted it gets. I think we would need to make a decision whether specific training, as such, is required and training means that there's an assessment. So you've got to have some way of actually checking that the person is actually understood and got the message.”* P3 |
|  | **Study 2A:** No relevant evidence found |
|  | **Study 2B:** *“It (training) is not tailor made to each individual, it’s quite generic for everyone. People often lose interest. So, if they were made for a particular area or a particular speciality, then they would have better impact..."*  Doctor1  *“if people are going to use their own device at work, it would be reasonable to require some web based training specifically about what's acceptable use of that and how to protect patient information...having a clear training when you are using your own devices to ensure that everybody's got the tools on their device and knows how to use them before they need is important, because if the first time you're having to sort this out is when you actually need to use that tool, then it's not going to work for most people.”* Doctor4  *“Training, it's quite long. And I know that it needs to be long because they need to include absolutely everything. But who has the time to sit there and actually read all?”* Nurse4 |
| **R1.15: Training should be mandated and incentivised** | **Study 1A:** No relevant evidence found |
|  | **Study 1B:** *“We will almost certainly get to the point in the not-too-distant future where things like cybersecurity awareness and BYOD use will almost become part of those mandatory competencies that people go through every 12 months as a result to maintain their credentialing and their ability to do their job.”* P5  *“the board has accepted the fact that certain parts of the security training that must be mandated. So it's not an optional activity anymore. They've already accepted that people who will fail their phishing simulation exercises will have to go through mandatory training.”* P1  *“Who's going to pay for ... Basically, the time ... If you said, "You need to do two hours of cybersecurity training," those 50,000 staff across the sector are going to say, "I want to be paid for that two hour of training."* P6  *“It's a real challenge. How do you fit education, policy, technology changes into that clinical environment when the workload doesn't actually a slot in their day for cybersecurity or BYOD education. It's not part of the job description that incorporates that.”* P5 |
|  | **Study 2A:** No relevant evidence found |
|  | **Study 2B:** *“we've got training that we need to complete. I refuse to do it off the clock. If it's mandatory, you should be paid to do it and it should be done at work...."* Nurse2  *"..We are supposed to know on our own and supposed to use the online tools, in our own personal time. We don't get any particular protected time to do those trainings…So there could be protected time to do these kinds of activities, maybe also making them more rigorous and mandatory..."* Doctor1 |

**Stage 2: IDENTIFY**

| **RECOMMENDATION** | **EVIDENCE/EXAMPLES** |
| --- | --- |
| **R2.01: Roles, responsibilities, and liabilities with regards to protecting PHI as well as employee personal data should be explained through a user agreement** | **Study 1A:** No relevant evidence found |
|  | **Study 1B:** *“you have new users coming on board, including employees, and the contractors and vendors who are also presented with an AUP. And they're asked to sign off on that before they are accepted as a user within this environment…”* P1  *“People would be made aware of them (security policies) when they joined the organisation, and you would sign up then that you would abide by the policies…”* P2 |
|  | **Study 2A:** Participants who were supplied a BYOD user agreement had better mean knowledge scores across all BYOD security elements asked in the survey  Mean knowledge score for participants supplied with a BYOD user agreement was found to be higher (3.20) as compared to those for whom it was not supplied (2.69). |
|  | **Study 2B: *“****I guess one more question that I will bring to this is where do you draw the line? Are you putting this on the clinicians? Are you putting in this on the hospital? Or how far if you're using personal devices? What are the expectations? Or what are the boundaries?”* AHP3  *“…I had received hospital policy as part of the induction where you have to sign some agreements and one of those is an information policy. And sort of acceptable use and things like that…* *by agreeing to do something, you certainly become aware that you have to abide by those. And I'm sure there's a bit more of an uptake or there's more compliance by having to ask people to agree on the terms. And obviously then it also acts as a sort of binding thing…””* Doctor5  *“…So when you sign up (for BYOD use), you get a personal device usage policy that you sign to say that you will abide by and then updates are sent out by IT…”* AHP4  *“I think more transparency around how your personal devices information is used or stored would be helpful for some people. There are lots of people that have no idea what that is, and that's just confronting for them to know...Some senior members of staff are a little bit more suspicious of technology than so our junior members of staff that who have sort of grown up with technology being the, be all and end all, and they understand why they password protect things and they understand secondary notifications because that's the life that they've lived. Whereas our more senior members of staff are less, I guess, natural, adaptive to that…”* AHP4  *“There just needs to be a transparent way of communicating to clinicians who inherently aren't that technologically advanced. Gaining their sort of trust and making sure the implication in itself is informed the decision in itself. Same way goes for me. If I'm examining a patient and stuff, I need to gain their informed consent and tell them all the risks and benefits of doing X, Y, Z, per se. I expect the same for IT telling me if I wanted to do something on my personal device.”* AHP1 |
| **R2.02: Clinicians not wanting to use BYOD should be provided with alternative options such as corporate devices** | **Study 1A:** No relevant evidence found |
|  | **Study 1B:**  *“Nurses don't want to carry their devices around. They just want to come to work and use a device that isn't their responsibility, that they don't have anything to do with afterwards. They just want to go back to their mobile. They don't want to have all this other stuff. They have to do the real work, in terms of they need a screen that's big enough to actually type and do stuff with, whereas a doctor, they're more reviewing the data, so they could get away with a tablet, they could potentially get away with a phone…they're going to multiple hospitals, so they just want to be able to log into the next hospital and even just see their patients for the day, see their X-rays or whatever, whereas a nurse, it's more, "When I go to work, I'll deal with that problem there. I don't want to bring my work home with me," whereas clinicians are probably more looking, "I want to check my patients at home," or, "I want to check my patients before I actually get to work." It's just a different focus.*“ P4 |
|  | **Study 2A:** Difference in the preferences, nature and extent of BYOD use among different clinical groups such as doctors, nurses, and allied health professionals was found |
|  | **Study 2B:** *“I think it should be an opt-in approach that you are more inclined to use it. You don't have to. It's not mandatory. I'd be more inclined to do that… Having the option to opt out as well if you decide it's (BYOD) not for you and you want to just get rid of it and not be a part of this experience anymore.”* AHP1  *“If we say no (to BYOD) then we won't have any access (to hospital services). There's no option. It isn't a choice. So you either opt in or you're out.”* Doctor1  *“..it’s just a full no-no (using BYOD), I don't think a personal device has any place in professional workplace. The other thing is also that you want to maintain your own personal space and your own personal emails and communications that may be related to your family or to your own health and well-being or legal matters or whatever, separate from your work…* *I think it is a bizarre expectation that you would be using your own phone plan to facilitate that professional work..””* Nurse2  *“..I think it's more also for work life balance, you don't really want your phone coming out with a lot of patient problems and messages from patients when you're trying to enjoy your time away from work. I think it's a big cause of clinician fatigue…”* AHP3  *“And well, to be honest, we haven't been offered any hospital devices. I know that if we are in senior positions, we might be offered that. But other than a desktop which is shared with other people, really my own devices is my only option for using that.”* Doctor5 |
| **R2.03: All devices intended to be used for BYOD purposes should be registered and access to hospital services should be provisioned** | **Study 1A:** No relevant evidence found |
|  | **Study 1B:** “*Doctors that want HS5 email or Wi-Fi connectivity on their phone, it's fairly simple. They just call and are provided with the URL. They go to the URL and there’s just a couple of step process where they put in their active directory credentials and once they log in, the profile is pushed through to the end device. It's quite quick.”* P6  *“We can provision access and we can authenticate a user accessing a service via the phone, email for example”* P6  “*if someone wanted to bring their own device, they would contact the help desk and say, "Can I have email on my phone, can you set that up for me?"* P2 |
|  | **Study 2A:** No relevant evidence found |
|  | **Study 2B:** *“…when we ask for access to the hospital records on our devices, we had to register our device. First time we have to give them the IMEI numbers. Then you'll get a call back from IT department confirming it's the right person. So it goes through sort of a formal process. You can get your hospital email added to your phone without too much paperwork. But anything more advanced where you would like to log into the hospital VPN, that's a bit more rigorous. And that will involve much more paperwork….”*Doctor1  “*To use the hospital Wi-Fi or the EMR, I had to specifically get a sort of token that's unique for that and that allows me to use just one device. So, they have a sense of how many devices and what devices I'm actually using with the EMR. I think at one hospital, but this is not a current hospital, I have had to actually provide some additional information. I can't remember if it was like the Mac ID or something like that, about the actual device before they would give me access to the network.”* Doctor5 |
| **R2.04: A device or OS permit list should be maintained to make sure only secure and tested devices are allowed to be used for BYOD purposes** | **Study 1A:** Prominent device types (smartphone:100%; tablet:70%; laptop:70%) and operating system types (apple:100%; android:96%; windows:74%) were reported to be mostly allowed for BYOD purposes, as compared to device and OS types not prominent (Wearables: 17%; Blackberry: 26%; Symbian: 13%). |
|  | **Study 1B:** *“The challenges essentially stem from the fact that there are compatibility challenges between, say, the wireless LAN controller, and a certain version of, say, iOS or Android that may be incompatible. Because as you can imagine, the iOS will be released and updated on the phone a lot faster. And that incompatibility can cause issues when it comes to connecting the devices to the network. This in turn increases the burden on the service desk when the calls start to come in that people can't connect to the wireless.”* P1  *“…when we were rolling out our telehealth capability at HS3, all our consulting rooms weren't video enabled, in any shape or form. And so it was a natural thought, a quick and easy way of doing that is iPads. But there's is a whole other issue of updating infrastructure and operating systems and (checking) compatibility with all the different systems. It's actually quite a nightmare!”* P3  *“..we use Cisco ISE…basically It gives us the ability to prevent anyone from connecting a device onto our network. We can put policies in place, for example unless the devices meet certain criteria e.g. it has this level of anti-virus installed...only that device is allowed on the network.”* P5 |
|  | **Study 2A:** Several device types were reported to be used by surveyed clinicians for BYOD purposes (95% smartphone; 62% laptop; 25% tablet). |
|  | **Study 2B:** *If your device is old or has been outdated, it does become harder to use these kinds of facilities. You need to keep your system updated. And some people, I know they don't like to update their phone, but if you want to use your personal device, you are supposed to. I have been in occasions where my remote access was blocked because I hadn't updated my phone yet. That's a big challenge. If you do not want to do that, then you sort of lose access. …"* Doctor1 |
| **R2.05: Role based access control (RBAC) and principle of least privileges should be used while granting access to hospital services or data** | **Study 1A:** No relevant evidence found |
|  | **Study 1B:** *“So we've got user access management and obviously different people have different role types have access to different applications, so that's controlled yes.……so depending on your role you'd be given access to… the EMR or the finance system or the HR system. Whichever applications are needed to do your job…”* P2  *“If we had access to an integrated electronic medical record then if that person was accessing via a BYOD mobile device then their access to information would be controlled by their profile on the EMR and they would only have access to certain things that they could see as a result of the definition of their role and their permissions. At the moment, given the fact that they really only have access to email from a BYOD device, we're still stuck in that situation whereby we can't control what they do on the device, we can only control what they have access to.”* P5 |
|  | **Study 2A:** Different nature, scope, and level of BYOD use was found among participants depending on the clinical role |
|  | **Study 2B:** No relevant evidence found |
| **R2.06: Several training modes should be used as per requirement and situation including induction training, emails, posters and flyers, online cybersecurity modules, class-based trainings and presentations, phishing campaigns, as well as targeted training and communication to critical staff** | **Study 1A:** No relevant evidence found |
|  | **Study 1B:** “*I think there's education there, it doesn't have to be formal sit down, hey this is cyber-security 101. But very much... Sending out the spam email that's actually educational, to say, oh you clicked on that link and you really should not have. Did you not hear what we said about don't click on dodgy links. So phishing campaigns, things like that. Just awareness through the regular things that they would look at, whether its hospital sent communications. But even I think if we could get to them through their colleges and the formal education and the formal journals that they read. The occasional piece in there about patient privacy or cyber security and hey, yes it can affect you. If you can attack it from a number of angles, that would be great. And keeping it related to what they do rather than compliance type focused.”* P2  Table on training modes |
|  | **Study 2A:** No relevant evidence found |
|  | **Study 2B:** *“…the more senior people in our department and even some juniors don't fully recognize the potential risk of communicating through unencrypted apps end to end. They assume that Messenger from Facebook is a perfectly private chat per se between clinician and clinician.. Not many people do (understand the risks) in that sense in our world right now..."* AHP1  *“So do the reorientation program and any new doctor starts in the hospital, they get a reorientation. And one of the parts of the orientation covers is the confidentiality and safety and online access. And I think the third part is mainly people who are more keen, they go and seek help if they don't understand things, copy, they will go and ask PACS administration in IT and pretty much it…we have an online learning tool, everyone has a profile, portfolio and on their dashboard they will have training modules there, which will tell them your module has expired and you need to renew it and you need to go back and read it. So those things are existing there, but I still feel not too much stress is not put on this. Some people may be lagging behind...* *“I think it's a very important part before giving people access to these kind of apps (BYOD apps), people should know what they are encountering and how to make full use of it, and also be safe.”* Doctor1  *"..At this stage, I think majority of them (clinicians) don't really know what the rules are, what the safety practices are. Most people do it on the base of what they feel is right…"* Doctor1  *“I think the growth at which we're expected to use our personal devices is not matched by the education around this, how to use them safely. Like, I think the shift to being able to do all these things in our personal devices, certainly has been much quicker than learning all the safety aspects of it. So, I think concomitant education around that and awareness of the potential latest risks and strategies to avoid, exposing both our patients and our personal data, would be helpful.”* AHP4 |

**STAGE 3: Protect**

| **RECOMMENDATION** | **EVIDENCE/EXAMPLES** |
| --- | --- |
| **R3.01: Identity and Access Management (IAM) solutions should be used to manage authentication of BYOD users** | **Study 1A:** Majority of surveyed hospitals (61%) reported use of IAM solutions for BYOD security purposes  Role based access control (RBAC) was reported to be used by 30% of surveyed hospitals for BYOD access management |
|  | **Study 1B:** *“…doctors that want HS4 email or Wi-Fi connectivity on their phone, it's fairly simple. They just call and are provided with the URL. They go to the URL and just a couple of steps for the (registration) process where they put in their active directory credentials and once they log in, the profile is pushed through to the end device…”* P5  **:** *“So we've got user access management and obviously different people have different role types have access to different applications, so that's controlled yes…”* P2  *“..we use Cisco ISE…basically It gives us the ability to prevent anyone from connecting a device onto our network. We can put policies in place, for example unless the devices meet certain criteria e.g. it has this level of anti-virus installed...only that device is allowed on the network.”* P5 |
|  | **Study 2A:** No relevant evidence found |
|  | **Study 2B: *“****we have access to the hospital records directly from the home that has been safely provided to us by the hospital. We have got to credential ourselves with the login password and they send you a phone text with a password login so it becomes secure and once you logged in and then you can access any patient records.”* Doctor2 |
| **R3.02: Strong password practices such as use of unique, complex, alphanumeric, and long passwords should be enforced for hospital service available through BYOD devices, especially when sensitive patient data is to be accessed** | **Study 1A:** No relevant evidence found |
|  | **Study 1B:**  “*The password rules were also quite light in terms of what you needed until recently. Now as part of the 72 (compliance) controls, it actually specifies how often you should change the password and how complex it should be. So we've made some changes to align better with the controls…”* P2  *“what you're trying to do is you try to draw a balance, which is if we have 10 characters or eight characters as a bare minimum requirement with complexity in place, and if we want to extend the length of the password, then it's like the government taxing you more in one space and giving you relief in another to keep that balance. So what we then do is we basically increase the length of the password, but we understand that if we increased the length, it will be harder to remember, and people will then write it down somewhere, which is not good either. So in return, what we then do is we basically say, "Your password, it's long because it's harder to crack, but will never expire if you adhere to the new policy."* P1  *“…we've got 13-character passwords currently…”* P4 |
|  | **Study 2A:** Strong password practices such as use of a device locking mechanism (92%), no sharing of passwords (68%) and use of strong alphanumeric passwords (63%) were found among surveyed participants |
|  | **Study 2B:** *“…In the last sort of 18 months, it's really changed with having to have longer passwords that are changed more frequently. Like up until two years ago, I had the same password I had when I started. That's not exactly great for security. But the frequency that we change our passwords now has changed. Also, we can't just add an extra number on the end of it. So it has to be a completely new password. So some of the password strategies are certainly more rigorous than it has historically been…”* AHP4 |
| **R3.03: Single sign-on should be used to minimise logins required to access hospital services through BYOD devices** | **Study 1A:** Improvement in clinical workflow and productivity was ranked as the topmost reason for allowing BYOD.  Most participants (90%) also believed that clinical workflow integration is either a ‘very important’ or ‘important’ step in BYOD security management. |
|  | **Study 1B:** *and we've got methods to do single sign on... It's probably one of the more streamlined sides of our technology and we try and make it as efficient as possible.”* P2  *“..we use enterprise reduced sign-on. We don't have a single federated identity management system across the hospital, but we have made it as simple as possible. And sometimes there's some legacy single sign-on pieces around the organisation, which just improve the user experience a bit…”* P7 |
|  | **Study 2A:** 37% surveyed clinicians believed that the hospital BYOD security processes hampered their clinical productivity in some way, 28% gave a neutral rating, 15% rated “minimal” and 20 % rated “no impact at all”. Higher impact ratings were seen for doctors. |
|  | **Study 2B: *“****So the hospital just recently implemented single sign on, which means that simplified it, but single sign on, there's still a couple of extra bits to that. So I can access most of the hospital information on single sign on, except I still have to authenticate for ordering and radiology…before that, I was a little bit complex because I do both anaesthetics and emergency, and the way the world worked I actually ended up with two user names.”*Doctor4  *“I think that's the main challenge is to sometimes you feel it's too cumbersome to log in and get asked to put in your code three, four times in one single session because you're switching from one application to another.”* Doctor1  *“One of the barriers to just instantly communicate to the target clinician/s, let's say EMR at my hospital, per se, you have to log in every five minutes. Everyone just basically hates that sort of aspect, that barrier of just, "Why can't I just see what I need to?" And having to log in every five minutes or so. I know it must be there for security purposes, but it's just putting a barrier to our satisfaction, one, and the uptake, two, probably. It does really slow down our workflow.."* AHP1 |
| **R3.04: Where possible, centralised identity management systems should be used based on credentials supplied by government agencies** | **Study 1A:** No relevant evidence found |
|  | **Study 1B:** No relevant evidence found |
|  | **Study 2A:** No relevant evidence found |
|  | **Study 2B:** *“…And also the clinical records, you have to have a Cerner login, which is issued by the department of health. So they know that you are registered to have access to those things. So I think it has improved it in the sense that you can't just walk off the street and pick up the record and read it…”* AHP4 |
| **R3.05: Minimally invasive technologies allowing central management of all types of devices within and outside the hospital network should be used to enforce security controls and policies remotely** | **Study 1A:** Several device (smartphone: 100%; Tablet: 70%; Laptop: 70%; Wearable/IoT devices: 17%) and OS types (iOS: 100%; Android: 96%; Windows: 74%; Blackberry: 26%; Symbian: 13%) were reported to be allowed for BYOD purposes  Technologies such as MDM (43%) and UEM (4%) were reported to be used for BYOD device management. |
|  | **Study 1B:** *“…We have a BYOD policy in place, but that only applies to mobile phones. So a user can bring their own mobile and we use AirWatch MDM policies and security around that device in terms of giving them access to HS5 email and also to HS5 Wi-Fi. That's pretty much all we allow from a BYOD perspective.”* P6  *“It’s (BYOD) widely used and the issue here is we have doctors taking photos of incidents or episodes of patient care and then chatting it with other doctors on their personal device… (If) a doctor brings in their own personal device that he or she wants to use and if you then apply all those restrictions on their own machine, I don't they're going to like it… so aspects like locking down the machine and having measures in place to prevent any breach, for example anti-virus, anti-malware, locking down the machine as we don't have administrative privileges isn’t possible.”* P6  *“I think the challenge is if you've got a user who wants to bring a device into the work environment, then you have got to make sure that they are willing to provide you with the ability to control that device should it go missing or fall into the wrong hands. Ideally you don't want to be storing any information on that device. If someone gains access to it and there's content on there that you don't want people to be seeing.”* P5  *“We have had it on our wish list to implement a mobile device management solution, which people, to access HS6 Health services, they would have to enrol in that, which would provide us posture management of the endpoints. That would significantly add to our administrative workload in the hospital, because all of a sudden all those devices come within our responsibility, I suppose. I suppose the main barrier there is just funding for the solution and all of the administration required to manage that. That's why it really hasn't been addressed. But if we were to improve the security of BYOD, we'd have to implement an MDM and probably fund a resource to manage all those devices.”* P7  *“The challenge is to maintain compliance, especially when writing a policy around device models that the user can bring into the organisation because not all devices would be compatible with most of the applications that need to be provisioned onto that device. And technology is constantly evolving on a daily basis. One day you've got Apple-6, the next day you've got Apple-15… So again, having to maintain that level of compatibility with the technology as it's changing is especially challenging in a BYOD environment, especially around developing that policy and the framework around BYOD. And not just the device, but also the operating version of the operating systems because a major upgrade on an IOS can have a major impact on how you access.”* P6  *" I think the only thing that's really acceptable from clinicians is the cloud-based approach, where I'm using this (personal device) as a thin client in terms of my work. It's just internet. Servers are out there I'm just accessing. And I think that's the only way that BYOD strategies and policies can probably work effectively in a health system”* P3 |
|  | **Study 2A:** Several device types were used by participants including smartphone (95%), laptop (62%) and tablet (25%) for BYOD purposes  Only 2% participants were willing to provide full administrative privileges to hospital authorities for BYOD security purposes  Majority of participants (62%) not using BYOD due to personal preferences did so because of fears of personal privacy intrusion |
|  | **Study 2B:** *“...Never, I wouldn't trust them (hospital management) within a million miles. They should never be allowed to have any sort of input or control over my device...So no, they are not getting anywhere near my device...If they wanted to build an app that could interface with their system in an efficient manner, then I'm comfortable with it. But to have anything that would have any ability to control or influence my device's function, never...”* Doctor4  *“I personally wouldn't feel happy or safe to give entire access to my device to someone outside. Yeah. In the ideal world would be where the IT administrators from the hospital would have control over the hospital data only...But what we are having at the moment is you give entire control to them. So if something is suspicious or they are not happy with whatever is happening, we have given them permission. That's the only way you can get access. If we say no then we won't have any access. There's no option. It isn't a choice. So you either opt in or you're out…”* Doctor1 |
| **R3.06: Staff should be provided access to secure enterprise applications which have security controls such as access management features, no print screen or copy data functionality for patient data, minimal cache use, PHI encryption as well as preventing display of notifications with PHI** | **Study 1A:** No relevant evidence found |
|  | **Study 1B:** *“So we've got user access management and obviously different people have different role types have access to different applications, so that's controlled yes.……so depending on your role you'd be given access to… the EMR or the finance system or the HR system. Whichever applications are needed to do your job…”* P2  *“we're looking at mobility apps for our phase two, and it will be interesting to see how much they're really used. I think, probably, all people are going to be doing on those is looking at results, maybe, somebody asks you on the fly, "What's the haemoglobin level on this patient?" And if you can find it quickly on your handheld devices as you're walking around, well, that's great”* P3 |
|  | **Study 2A:** No relevant evidence found |
|  | **Study 2B:** *“..For my public hospital work, I use my smartphone with patient medical records. I can also look at the lab results and I can also look at the radiology images on my phone. And the same thing, I can do it on my laptop and my desktop. And I do it through secure apps which have been installed in our system by our IT Department, they need a pass code and soft token before you log in”* Doctor1  *“So I would have to enter my passcode into the phone and then I would have to enter my hospital network and password into the EMR app and that would give me access to the app.”* Doctor5  *“Yes. It's Microsoft two-factor authentication, so you need a login and a password from the institution or server from the hospital to be able to have access to it. And then on top of that, you need Microsoft authenticator to authenticate you into your devices.”* AHP4  *“…there is a cache memory in my phone and I'm worried that if my phone is compromised, I would be putting a patient's confidentiality at risk.”* Doctor1  *“People (clinicians) use screen capture, so it's really hard to stop them. There's always a work around… A lot of times when you have an app, let's say this app is connected to the hospital network, the person with the device, they follow the person with device local policy. Yeah. But then if you get this app to only follow a particular policy and not everything on the personal device, then the data may be secure, a lot more secure. Like if you can stop screen capture on your local device. Like whenever you open this app, you cannot screen capture, then the security might work.”* AHP2 |
| **R3.07: An enterprise integrated application system should be used, where both clinical and non-clinical apps are provided through a common platform such as internal regulated app store and have a unified access mechanism, with single sign-on enabled** | **Study 1A:** No relevant evidence found |
|  | **Study 1B:** *“We're going from on-site email to cloud email. It's the whole Microsoft move to cloud and cloud storage, and also providing a full bubble of online stuff that you can get to at home so that you get the same experience at work as you would at home.”* P4  *“I think the challenge you have in healthcare, which I think is quite unique is that you can end up having quite a large number of vendors providing you solutions to meet the varied needs of different areas. E.g. we have specialist areas in oncology, obstetrics, emergency, medical, surgical, in-patient, out-patient, mental health, allied health and community care. There are no vendors that provide a single solution to meet all of those needs so therefore our teams end up having to manage a very large suite of applications to support all of those… Trying to meet the requirements of a health service that employs over 2,000 people with probably two or three dozen major applications and as many different scenarios, we're not necessarily geared up to cater to and to support that level or complexity...* *Ideally strategically moving forward, consolidating a lot of these applications into something like a holistic fully integrated clinical information system or EMR that is hopefully designed with WebFirst or MobileFirst in mind. It's hosted and it has a number of interfaces to it that you would access the application securely and integrate it simply with our active directory and whatever security policies we have.”* P5  *“Definitely, less applications, one unified interface obviously would make it much easier to manage and maintain as opposed to the plethora of applications we have at the moment.”* P6  *“At the moment we use at least three different systems just for patient stuff…so people do not want to have different passwords for every application… it's just bad, it's time consuming… I think, the way of doing it is to potentially integrate all these different applications as well. So it feels more like a seamless experience.”* P3 |
|  | **Study 2A:** Clinicians were reported to use several clinical and non-clinical application on personal devices for BYOD purposes such as productivity apps like email or calendar (84%), clinical communication (77%), drug reference (58%), medical apps (56%), EMR apps (51%) and clinical photography (40%), with an internal hospital app store reported to be used by only 34% participants. |
|  | **Study 2B: *“****They (Hospital) have different apps for different things. So they have one app for your payslips, one app for your shift times. I believe that they should all just be put together. And they have one app for patient care and then they have a different section for documenting what you did for that patient. I also believe that should all be together because it just makes your work feel overwhelming. You have to constantly click out of one app to click into another, if that makes sense.”* Nurse4  *“..For my public hospital work, I use my smartphone with patient medical records. I can also look at the lab results and I can also look at the radiology images on my phone. And the same thing, I can do it on my laptop and my desktop. And I do it through secure apps which have been installed in our system by our IT Department, they need a pass code and soft token before you log in…”* Doctor1 |
| **R3.08: Critical and non-critical infrastructure should be separated by using measures such as network segmentation, guest networks, or dedicated staff networks** | **Study 1A:** Majority of surveyed hospitals either had a separate guest network for BYOD devices with limited access to hospital services (48%) or didn’t provide access to hospital network at all (13%), with only 39% allowing full access to the hospital core network. |
|  | **Study 1B:** *“all BYOD devices, they cannot connect to the internal network… And if they (users) for example, bring their own Mac, they can connect to the wireless, but then the wireless only gives them limited access…* *all my communication to the internet as a user go through a proxy, which protects me from, say, malicious downloads or viruses...”* P1  *“it's just like internet at the airports. You go you get access to public Wi-Fi, but that is a totally separate internet connection to our HS5 administration internet with security policies in place to prevent them from any access into our network…* *So if you were to come to HS5 with your own personal machine and find a spare port on the wall and try to plug in your device, your machine would not be allowed on the network and would be blocked…* *these are the sort of tools that we use to prevent users from bringing BYOD devices and plugging into our network and unknowingly infiltrating malware onto our enterprise network.”* P6  *“The other thing that has been happening is that staff have learned how to access the official network on their own devices… so there is potential risk there in terms of people connecting infected computers to our network…And so that's why I've been really pushing hard to try and get out IT people to have this dedicated staff Wi-Fi band, so that people could start using their own devices and get the best of both worlds, we're following the rules and we're also enabling optimal patient care.”* P3  *“...there's many different wireless SSIDs available within the hospital. For HS6 owned assets, there's a secure network, and, as I said, there's also the public guest Wi-Fi access network. They're segmented to provide isolation…”* P7 |
|  | **Study 2A:** No relevant evidence found |
|  | **Study 2B:** No relevant evidence found |
| **R3.09: Hospital data should be encrypted both in rest and in motion, which includes both stored data on BYOD devices as well as data in transmission originating from or destined to such devices** | **Study 1A:** No relevant evidence found |
|  | **Study 1B:** *“…We're running our own malware protection (tools), we're running Cylance and other types of security products, and also, we use Bitlocker, so the hard drives are actually encrypted on our service.”* P4 |
|  | **Study 2A:** No relevant evidence found |
|  | **Study 2B: “***Well, my device, the whole device is encrypted. So that's protected in that way.”* Doctor5  *“I keep a set of photos of interesting cases for teaching purposes, but they are on my device. They are on my home computer on an encrypted database and so on and so forth. It's much higher-level security on my home computer that I'm pointing to over there of course because it's there.”* Doctor4 |
| **R3.10: Remote access through BYOD devices should be enabled with secure technologies such as VPN and Secure Web Gateways** | **Study 1A:** 65% participants reported use of VPN, whereas 13% reported use of API/Secure Web Gateways by their hospital for BYOD security purposes |
|  | **Study 1B:** *“So the health service has had a BYOD platform, they have got quite a good VPN set up, so access from home using your own device is mature. And initially, there, it was quite constricted, and there was fees and everything to be able to get onto that, but that's been freed up, with COVID-19 particularly and clinicians working at home. So they've made access to that a lot less bureaucratic, so people can, just through logging on remotely, even if they're in the hospital, they could potentially use that for their own device.”* P3  *“We do offer remote Citrix to users so they're able to access applications externally, but that's HS5's remote Citrix application.”* P6 |
|  | **Study 2A:** No relevant evidence found |
|  | **Study 2B:** *"…So the hospital provides me with a VPN access through what's called Citrix receiver, because I use Citrix in their system. So, I have to open a web browser, log in, then open the Citrix receiver, and that then opens the VPN for me to access the hospital information…”* Doctor4  *“So, for the private hospital practice, they have set up a home computer for me and they have a sort of a VPN established. I can sort of do any work which I usually do at the office, from my home. But it means a dedicated line, we can establish them at home and establish a dedicated workplace.”* Doctor1  *“I'd VPN into the hospital if I'm not onsite… there is a VPN set up to be able to work directly on the network. There are some tasks that required that.”* Doctor5 |
| **R3.11: Staff should be provided a dedicated enterprise storage platform for hospital data storage** | **Study 1A:** 17% participants reported use of a private cloud by their hospital for secure enterprise storage purposes |
|  | **Study 1B:** *“…we still allow them (users) to use Dropbox, because we don't have an alternative. We've just bought Office 365 licenses, and we do eventually want to get them all onto an enterprise OneDrive, and then we'll lock down everything else inside the hospital that they wouldn't be allowed to get to…”* P4  *" I think the only thing that's really acceptable from clinicians is the cloud-based approach, where I'm using this (personal device) as a thin client in terms of my work. It's just internet. Servers are out there I'm just accessing. And I think that's the only way that BYOD strategies and policies can probably work effectively in a health system”* P3 |
|  | **Study 2A:** No relevant evidence found |
|  | **Study 2B:** *“..I mean, it's very convenient to store data on your personal device. But what if you're uploading the data... What if your phone is automatically backing up your data to iCloud, your personal iCloud*?..” AHP3  *“..And something like Google Drive that's hosted by the hospital would be helpful…”* Doctor5 |
| **R3.12: Technologies such as virtualisation and containerisation should be used to logically separate personal and hospital data on BYOD devices** | **Study 1A:** As per participants, virtualisation and containerization were reported to be used in 39% of the surveyed hospitals for BYOD security purposes |
|  | **Study 1B:** *“…We’ve got Citrix (virtualisation technology) ... You can't actually copy files into Citrix or out of Citrix, so that's fairly tight from a data leakage perspective, and I think it's fairly secure. It's quite a contained bubble…”* P4  *“…we decouple everything by Citrix. We don't manage the cybersecurity posture of the endpoints at all, we just decouple everything through Citrix and leave the device up to the owner's management… Largely, we take a position of, it's decoupled from our network, it doesn't put us at risk. You manage your own security; we'll just provide you access through Citrix.”* P7 |
|  | **Study 2A:** 76% participants who stored patient media on personal devices did so along with personal data in the same folder or location. |
|  | **Study 2B:** *“So the only way to log onto our system to get the patient information is through Citrix. That's the only way. So that is up to Citrix. And once we log onto the system, it's the intranet. So the intranet is looked after by the IT department and they have quite a few firewalls, there's more than one firewall in place. And then there's virus scan and all that. So because of that, the system is actually quite slow because we have like anti-virus scan. Yeah, so all the data is actually within the intranet (local network).”* AHP2  *“Yeah, that (allowing partial administrative privileges for only hospital data through containerisation) would be good. That'll work, yeah.”* AHP2 |
| **R3.13: Clinical staff should be provided a dedicated clinical communications/collaborations platform on BYOD devices which is encrypted and is integrated with all hospital systems. The platform should have features such as photo sharing and electronic consent taken from patients** | **Study 1A:** Secure clinical communication, messaging, or file sharing applications were reported to be used by 30% of the surveyed hospitals for BYOD security purposes  56% participants reported ban on personal file sharing or communication apps for clinical communication purposes as a policy measure |
|  | **Study 1B:** *“We are looking into some of those communication tools at the moment. One of them is a product called My Beeper that allows for the secure communications and integration if required into our patient administration system. It allows for the collection of digital photos and gaining consent for the collection of those photos and provides an audit trail over those communications as well."* P5  *“we've just recently introduced a sanctioned form of doing that (clinical communication) called, myBeepr. And with that, now that that's available, we will be trying to close down all this other activity..,that's, the easiest way to think about that is that it is a substitute for WhatsApp, works similarly, and you have to be on our active directory to be able to access it. So, you have to be an employee, etc., and it complies with cyber security and encryption etc.”* P3 |
|  | **Study 2A:** User friendly modes of communication such as SMS (58 %) and WhatsApp (39%) were reported to be used for clinical communication, whereas only 32% surveyed participants used secure clinical communication platforms  Clinical photography through BYOD devices was also reported, with a high use among doctors (67%) |
|  | **Study 2B:** *“I'm pretty sure there are people in my department who use WhatsApp and stuff to communicate to other clinicians. I personally don't do that because I know WhatsApp is very leaky. I try and keep it professional as I can be.* *..If they're (clinicians) comfortable using WhatsApp to communicate to their friends, family, and stuff, they don't see an issue using it with clinicians as well. I suppose… it's convenience, it's habitual, it's their go-to messaging app…”* AHP1  *“it might just be a text message or a WhatsApp, where staff members may inadvertently or advertently might be intentionally passing patient information in a less secure manner.”* AHP4  *“And I know some hospitals have established communications systems internally. So Workplace for example, which I've used at other hospitals, which at least allow you to communicate directly with staff, but the take up has been poor in general. So I don't find that's a reliable way of communicating. I'm not certain that other people will receive or see the message...* *Because, there's not a good alternative tool (to WhatsApp). I mean, as I said, some places use things like Workplace, which is probably like Facebook version for work sites and is more secure. But , if you're in the middle of an emergency and you're wanting a quick answer from another clinician trying to teach them to install Workplace on their phone and make sure it's set up so that you can then send them a photo. It's just not feasible unless they already have that there. So I think that's why people just have not continued using it.”* Doctor4 |
| **R3.14: Staff should be provided with all necessary tools, services, or processes to be able to work on their devices securely and productively, following which policies could be enforced** | **Study 1A:** No relevant evidence found |
|  | **Study 1B:** *Also, never give an order that you know that people will not follow. It's never good for anybody... let's develop a policy which people can follow, when we have the enablers and the tools, and all the rest of it. I think we're really waiting to hit that sweet spot”* P3  *“From a clinical communication perspective at the moment, we don't have a particular tool or set of tools in place. Obviously what you don't authorize or provide then clinicians will find another way to do what they want to do. So what's that, it's probably one of those ones that is not authorized, it's not approved, but clinicians will use something to get around what they feel they need to do.”* P5 |
|  | **Study 2A:** No relevant evidence found |
|  | **Study 2B:** *“I think that's the major factor because I think that the way that it's (security processes) designed is going to affect the way that it's used. I think that there will be people who can always find an unsafe way or unorthodox way of using these systems. And you can't stop that entirely, but you can make it very hard for people to do that. And that then means that the person who doesn't really care is trying to use the easiest way of doing things. If the easiest way of doing things the correct way, they will use the correct way..."* Doctor5 |
| **R3.15: Staff should be advised to take self-protection measures such as minimising storage of patient data on device or deleting it when not required, and turning Bluetooth and Wi-Fi off when not using it** | **Study 1A:** No relevant evidence found |
|  | **Study 1B:** *“A lot of the time, they're (clinicians’) downloading the instructions ... just how to hook up the email, that's all they're really interested in. Probably, I think it's at the front, but that's really the only two sentences you need to do. It would be better to actually put ... Rather than having a full policy, it would be better just to have those two sentences on the instructions to make the educational email work, "Keep your iOS updated, put a password on your phone," that's it, and then the rest of the instructions. That's the closest thing you're getting to getting them to read the policy.”* P4 |
|  | **Study 2A:** As a security measure, 64% participants who backup their device data didn’t store/backup patient data on the device or their personal cloud or storage platform  40% participants connected their devices containing patient data to unsecure networks such as public hotspots, increasing chances of breaches if Wi-Fi or Bluetooth are kept on. |
|  | **Study 2B:** “ *I try to minimize saving anything on my phone, because of the confidential, information of patients. I don't download images; I view them but I don't save them.”* Doctor1  *“I try and keep patient information deidentified... Or trying to delete the patient information from the device if it's no longer required on there…”* Doctor5  *“We also don't store patient data on our phones. There are instances when we'll use our phones to take a photo of a wound or something like that, but the hospital policy is that once you've uploaded it to the medical record, you delete it from your personal device. So there's not a lot of patient data in itself that is stored on the phone, but it's certainly stored within the cloud devices that we use.”* AHP4  *“..Because if you put Bluetooth on or Wi-Fi on, someone can get your IP address and hack into it. So you must know about this. So better not put too much information on your phone.*” AHP2 |
| **R3.16: Patient consent should be sought and their data should be anonymised before actions such as clinical photography or storing and sending patient data to fellow clinicians through BYOD devices** | **Study 1A:** Majority of surveyed hospitals (77%) mandated seeking of patient consent for patient photography intended to be used for clinical purposes as a privacy measure |
|  | **Study 1B:** *"There are data policies in terms of personally identifiable information and things like that, you're not allowed to use that on the public side."* P2  *" there are also certain procedures in place, and some policies also exist that restricts users from exchanging classified information, whether it's patient or private, through emails, which means if their emails are compromised, there's no private or patient information that should go out."* |
|  | **Study 2A:** No relevant evidence found |
|  | **Study 2B:** *“We often take photographs of complex lesions or skin, which we need to refer on but of course that is after patient permission and also removing the identifying patients details on a specific picture that we take, which might include a chest x-ray or an imaging which does not have patient’s hospital UI number, name or address”* Doctor3  *“I always ask a patient that I have to take a photo to send it another clinician or when you refer people inter-department. And then of course, if they say, no, you won't. But if they say yes, of course you would send it and then you'll try and delete it…and in my little experience, patients have never said to me, "I'm concerned." And I think it also comes from the trust to that you know your doctor is using this information with your permission for a good outcome for you.”* Doctor3  *“first of all, I've got to think about whether or not the data is identifiable. And a lot of what I do isn't directly identifiable. Let's say for example, if they've got a rash. I don't text message the dermatologist saying, "Mrs Smith, aged 46, of 37 Sunrise Drive, Albuquerque." I just go, "46-year-old woman with a rash." This is the rash and I give him details about the rash. So it's not identifiable if it's intercepted.”* Doctor5  *“So ideally when they send me images, if I see a patient's name on top of it, I try to delete them ASAP and ask them not to send me the patient's name because it is Facebook. But I know I'understand that you have to delete it.”* AHP2 |
| **R3.17: Staff should be advised to use self-protection tools/techniques such as device locking, password protection for patient data, device encryption, anti-virus/anti-malware/anti-spyware/anti-theft and device tracking applications** | **Study 1A:** No relevant evidence found |
|  | **Study 1B:** *“I suppose through the policy just ensuring that staff do keep up with their anti-virus software, and that they've updated to the latest operating systems, which have inbuilt, now, ability to ward off hackers and what have you”* P3  “*Generally, we basically tell people that they need to patch OS to the latest version…we expect them to patch their OS, make sure they're using anti-malware, and that's about it.”* P4 |
|  | **Study 2A: S**elf-protection tools including anti-virus/anti-malware (49%) and device tracking/anti theft applications (10%) were reported to be used by participants |
|  | **Study 2B:** *“So I've got anti virus, which is regularly updated. So is also using malware checks next to it. There's nothing on my phone it can compromise. I don't transmit data one device to another without using a secure channel. So if I had to send something from a phone to my computer, I would use the hospital email at that time because usually encrypted and it's much more secure than using my personal email.”* Doctor1  *“Well, my device, the whole device is encrypted. So that's protected in that way. I try and keep patient data in a separate, encrypted secure mounts on the drive. And then, remove extra identifying information later or try and separate that out. I know there are some spreadsheets and things that have patient details on it that are not encrypted but I guess, I keep them password protected, being the actual Excel spreadsheets.”* Doctor5  *“so all of my devices are encrypted on the device, and I use six-digit passcodes.”* Doctor4  *“* *I've been pretty good in ensuring that my personal devices which is my laptop and my phone, I don't share it with anyone else, like with any members of the family or my friends, which is my laptop. And like I said it has an exclusive password.”* Doctor3  *“..I have got an anti-virus. I don't know how much does it cover, but it gives you that sense of security that I have got some sort of cybersecurity software on my device, which will probably help protect my privacy and the privacy of the patient’s records that I have on my devices at some stage…”* Doctor2  *“Yeah. I have a separate, like a private folder on the phone (for patient data)”* Nurse4 |

**STAGE 4: Detect**

| **RECOMMENDATION** | **EVIDENCE/EXAMPLES** |
| --- | --- |
| **R4.01: Malicious activities or potential vulnerabilities originating from BYOD devices should be detected using monitoring services such as SIEM, IPS/IDS, vulnerability scanning and virus scanning applications** | **Study 1A:** 70% surveyed hospitals reported use of anti-virus/anti-malware scanning, whereas 48% reported use of IPS/IDS for BYOD security purposes |
|  | **Study 1B:** *“…I may bring the (BYOD) device home and visit a website which plants a malware in my phone, and I then take it back to HS1 to connect, there are monitoring technologies in place such as IPS and SIEM services that will essentially not just look, but also warn the IT team of malicious activity on a certain phone…”* P1  *“We've got a new network, we've got Cylance (threat protection and intelligence software), Tenable (vulnerability scanning software) and Forescout (network monitoring and assessment software)… From a cybersecurity perspective, we feel that we know what's going on the network.”*P4 |
|  | **Study 2A:** No relevant evidence found |
|  | **Study 2B:** *“ We have more than one firewall in place. And then there's remote virus scan..So all the data is actually within the hospital intranet…”* AHP2  *“There's a risk of my own data being accessed from a malicious agent whilst I'm on our hospital network or an outside network and there's the risk of me introducing malware to the hospital or vice versa”* Doctor5 |
| **R4.02: Anomalous or abnormal behaviour within the hospital network should be detected through network visualisation and threat intelligence software** | **Study 1A:** No relevant evidence found |
|  | **Study 1B:**  *“We've got a new network, we've got Cylance (threat protection and intelligence software with ability to predict and detect anomalous behaviour), Tenable (vulnerability scanning software) and Forescout (network monitoring and assessment software)…”* P4 |
|  | **Study 2A:** No relevant evidence found |
|  | **Study 2B:** No relevant evidence found |
| **R4.03: Hospital BYOD users should be trained to detect security incidents or vulnerabilities such as phishing emails or messages** | **Study 1A:** No relevant evidence found |
|  | **Study 1B: *“****The (training) piece was on how to identify a suspicious email in terms of phishing, so how to assess it or how to respond to it. It was identified as one of the biggest risks, so we just addressed those. We wrote our own custom content to address the risks.”* P7  *“we run phishing campaigns…* *we monitor, say, click counts, so how many times a certain user has clicked on a URL that's coming through an email…* *And those phishing campaigns then translate into video training (for users to detect phishing emails).”* P1  *“Sending out the spam email that's actually educational, to say, oh you clicked on that link and you really should not have. Did you not hear what we said about don't click on dodgy links. So phishing campaigns, things like that. Just awareness through the regular things that they would look at, whether it's hospital sent communications.”* P2 |
|  | **Study 2A:** No relevant evidence found |
|  | **Study 2B:** *“..So it (training) was basically a phishing email, and if you clicked on it, went through to them and they were just pointing out that was the phishing email, you failed and then some video training…”* Doctor4 |

**STAGE 5: Respond**

| **RECOMMENDATION** | **EVIDENCE/EXAMPLES** |
| --- | --- |
| **R5.01: Unsecure devices and applications should be blacklisted and not allowed to be used for BYOD purposes** | **Study 1A:** Majority of surveyed hospitals (98%) didn’t allow unsecure devices such as jailbroken or rooted devices for BYOD purposes |
|  | **Study 1B:** *“…quite often those devices come in with malware on them. We've got that segmented from our own network, but occasionally it will come to our attention, and we've got to ensure that the device will be blacklisted until such time as they resolve the malware issue. That doesn't put us at risk, but it's not a good look when people are seeing malware coming from HS6 IP ranges...* *We’d blacklist the device using its MAC address through network access control measures. We would prevent it having access to our network... We certainly blacklist devices on a fairly regular basis,”* P7 |
|  | **Study 2A:** No relevant evidence found |
|  | **Study 2B:** No relevant evidence found |
| **R5.02: Any detected vulnerabilities originating from BYOD devices should be swiftly removed or remediated through vulnerability management software such as anti-virus, anti-malware, anti-phishing and email filtering** | **Study 1A:** Vulnerability management software such as anti-virus/anti-malware as well as email platforms with filtering and anti-spam capabilities were reported to be used for BYOD security management |
|  | **Study 1B:**  *“We're running our own anti-malware…we've definitely got some risk with phishing and cyber-attacks, no doubt, but we're using Mimecast for email filtering, which has reduced our phishing load significantly… Go back two or three years and we had thousands of phishing emails coming through, we had email accounts spewing out thousands of emails because they'd got credential phishing. We’ve tightened it up significantly than where we were two or three years ago…”* P4 |
|  | **Study 2A:** 49% respondents reported use of anti-virus/anti-malware to protect their BYOD devices |
|  | **Study 2B: “***So the local intranet is looked after by the IT department and they have quite a few firewalls. And then there's virus scan and all that.”* AHP2  *“..I have got an anti-virus. I don't know how much does it cover, but it gives you that sense of security that I have got some sort of cybersecurity software on my device, which will probably help protect my privacy and the privacy of the patient’s records that I have on my devices at some stage…”* Doctor2 |
| **R5.03: Staff should be advised to update their devices, applications, or operating system on a regular basis** | **Study 1A:** No relevant evidence found |
|  | **Study 1B: “***we expect them to patch their OS, make sure they're using anti-malware… the BYOD policy says, "If you want to use our email, which is Outlook ... you want to use the Outlook app, you must patch your phone, and if malware protection is available for your phone, you should have it on there.”* P4  *“…we ensure that staff do keep up with their anti-virus software, and that they've updated to the latest operating systems, which have inbuilt ability to ward off hackers and what have you…”* P3 |
|  | **Study 2A:** No relevant evidence found |
|  | **Study 2B: “***So I've got anti-virus, which is regularly updated. So is also doing malware checks... There's nothing on my phone that can be compromised...* *we have to keep our (operating) system updated.”* Doctor1 |
| **R5.04: Incident response procedure should be defined and communicated to all staff, who should report any detected security incidents such as loss or breach of device immediately to hospital IT or security department, who can deal with security incidents in a time bound manner** | **Study 1A:** 20% participants stated their hospital defined and communicated incident response procedure to staff and they are trained to report cyber incidents |
|  | **Study 1B:** *“If there is a malicious intent, then we have the necessary controls as part of the incident management protocol.”* P1  *“I think there's something in our policy that says if the user loses the device (as a security incident) they have to notify service desk or what have you and if you have provisioned any email or what have you that phone will then initiate deletion of that profile, but not the user data.”* P6  *“If you were discovered photographing patient information like that, there would absolutely be sanctions against you, if you were seen doing that, absolutely. There's HR departments here, and managers would report that, and you would be disciplined or given warnings, etc., absolutely.”* P4  **“***we've got a security operations center operating 24 by 7 (to deal with security incidents).”* P4 |
|  | **Study 2A: L**ow knowledge levels among clinicians about incident response procedure as mean incident response knowledge scores was less than overall average |
|  | **Study 2B: “***So first thing is I'll contact the hospital IT department straight away (on detecting security incident), and I will follow whatever advice they will suggest.”* Doctor1 |
| **R5.05: Government agencies should be notified immediately once a data breach is confirmed** | **Study 1A:** All participants reported that their hospital has to notify government or similar agencies of security breaches as part of compliance requirements such as relevant state health/digital health department (100%), Australian cyber security centre (43%), Office of the Australian information commissioner (29%), Australian digital health agency (14%). |
|  | **Study 1B:** No relevant evidence found |
|  | **Study 2A:** No relevant evidence found |
|  | **Study 2B:** No relevant evidence found |
| **R5.06: A thorough investigation of security incidents should be carried out and all learnings should be documented and communicated to relevant parties** | **Study 1A:** No relevant evidence found |
|  | **Study 1B:** No relevant evidence found |
|  | **Study 2A:** No relevant evidence found |
|  | **Study 2B:** *“I'm trying to imagine if there's any security breaches, and if the hospital requests for the device to be investigated, you have to show a to bring your own personal device for investigation...”* AHP3 |
| **R5.07: In case of loss or theft of BYOD devices, a selective data wipe or lock should be carried out, where the personal data is kept intact and access to hospital data or services is locked and data deleted** | **Study 1A:** For the lost or stolen BYOD devices containing hospital data, majority participants (48%) stated that their hospital remotely wipes the hospital data only while keeping the personal data intact, 43% took no action, whereas 10% perform a full device wipe. |
|  | **Study 1B:** *“I think the challenge is if you've got a user who wants to bring a device into the work environment, then you have got to make sure that they are willing to provide you with the ability to control that device should it go missing or fall into the wrong hands. Ideally you don't want to be storing any information on that device. If someone gains access to it and there's content on there that you don't want people to be seeing…If they call up and say the mobile is stolen, we can push through and initiate an enterprise lock on the device....so the policy in place says if the user loses the device, they have to notify service desk and if you have provisioned any email or what have you, that phone will then initiate deletion of that profile, but not the user data…”* P5  *“So we would not want people downloading patient information and images and things like that to personal devices except for work purposes. Then we wouldn't want it to stay on a device, we would want, if they no longer worked with us, that it was no longer accessible. So as a tool, typically through an MDM, you'd wipe the device when someone left but if it's a personal device you can't wipe the personal stuff. It's just a challenge how you manage that.”* P2  *“So we do not wipe any data on personal devices. That is definitely not done, because I don't believe the technology offers unlimited data wipe options, because if we trigger a data wipe, it will basically go in and delete even personal photos and what have you, that people have. And for that reason, we decided we won't venture in that space.”*P1 |
|  | **Study 2A:** No relevant evidence found |
|  | **Study 2B:** *“...Obviously, there's (the risk of) patient confidentiality, who knows what can happen if the device is hacked or lost in terms of identifying, stealing people's data or identity, selling them on the market...”* Nurse2  *“...it (BYOD) can sometimes be posed or taken as a threat to privacy for example, if the device is lost or is hacked.”* Doctor6 |

**STAGE 6: Recover**

| **RECOMMENDATION** | **EVIDENCE/EXAMPLES** |
| --- | --- |
| **R6.01: Staff should be provided with a secure backup option for hospital data such as private cloud or enterprise data backup solutions through which any lost data can be recovered quickly** | **Study 1A:** 17% participants reported use of a private cloud by their hospital for secure storage and backup purposes |
|  | **Study 1B:** *“…we still allow them (users) to use Dropbox, because we don't have an alternative. We've just bought Office 365 licenses, and we do eventually want to get them all onto an enterprise OneDrive, and then we'll lock down everything else inside the hospital that they wouldn't be allowed to get to…”* P4  *" I think the only thing that's really acceptable from clinicians is the cloud-based approach, where I'm using this (personal device) as a thin client in terms of my work. It's just internet. Servers are out there I'm just accessing. And I think that's the only way that BYOD strategies and policies can probably work effectively in a health system”* P3 |
|  | **Study 2A:** No relevant evidence found |
|  | **Study 2B: “***Hospital could actually set up a virtual device the way I want to with software that I need and then work on that remotely from home and in the hospital, which would at least then keep that data off my device or if the hospital set up some sort of shared drive that I could use from home that works in a way similar to Google Drive or something like that. So that I don't have to put the patient information onto my device other than in that temporary form when I'm using a program that's been cached or something like that…so something like Google Drive that's hosted by the hospital would be helpful.”* Doctor5 |
| **R6.02: Technologies such as virtualisation and containerisation should be used to ensure all data storage and backup takes place within hospital's own infrastructure, rather than the BYOD device itself** | **Study 1A:** Virtualisation and containerization were both reported to be used in 39% of the surveyed hospitals to logically separate personal and hospital data |
|  | **Study 1B:** *“…We’ve got Citrix (virtualisation technology) ... You can't actually copy files into Citrix or out of Citrix, so that's fairly tight from a data leakage perspective, and I think it's fairly secure. It's quite a contained bubble…”* P4  *“…we decouple everything by Citrix. We don't manage the cybersecurity posture of the endpoints at all, we just decouple everything through Citrix and leave the device up to the owner's management… Largely, we take a position of, it's decoupled from our network, it doesn't put us at risk. You manage your own security; we'll just provide you access through Citrix.”* P7 |
|  | **Study 2A:** No relevant evidence found |
|  | **Study 2B: *“****So the only way to log onto our system to get the patient information is through Citrix (virtualisation). That's the only way. And once we log onto the system, it's the intranet (local network).”* AHP2 |
| **R6.03: Staff should be advised to avoid storage or backup of hospital data on personal online storage or cloud platforms** | **Study 1A:** No relevant evidence found |
|  | **Study 1B:** *“We've got enterprise documents sitting in Dropbox and all sorts of other on-cloud storage platforms that we have no control over. We've definitely got data leakage that we need to sort out. ... we need to get OneDrive in, and we need to lock it down ... People don't have an alternative (at the moment), so they're using Dropbox and stuff to be able to get to papers and meeting papers on their iPad and on their laptop externally. That's, I think, probably our biggest leakage at the moment that really is pretty uncontrolled. From my perspective, that's where our risk is.”* P4  *“Our health service is also very unkeen on platforms like Dropbox…I suppose, being connected to some external gateway where large memory files are potentially being transmitted, because I suppose that's also how a virus is going to get in.”* P3 |
|  | **Study 2A:** 43% participants who backup patient data residing on their device, did so along with the personal data on a common personal cloud or data backup platform |
|  | **Study 2B***:” I do think generally, they should be aware of the risks also because for every healthcare professional…So if they, for example, have poor digital health literacy, and they think uploading things at cloud is secure, no one else can get into it." Then it's around education, as well. They might be aware that things should be kept private, but they might not be aware of what goes on in the backend that keeps it private or what steps do they need to implement in the front end to make sure that secure on the back end.”* AHP3 |
| **R6.04: Staff should be advised to disable automatic device backup to ensure hospital data residing on the device is not stored or backed up with personal data** | **Study 1A:** No relevant evidence found |
|  | **Study 1B:** No relevant evidence found |
|  | **Study 2A:** No relevant evidence found |
|  | **Study 2B:** *“..I mean, it's very convenient to store data on your personal device. But what if you're uploading the data... What if your phone is automatically backing up your data to iCloud, your personal iCloud*?..” AHP3 |

**STAGE 7: Assess and Monitor**

| **RECOMMENDATION** | **EVIDENCE/EXAMPLES** |
| --- | --- |
| **R7.01: Change management processes should be established which cater to changes in areas such as threat environment, legislative requirements, user requirements and business strategy or goals** | **Study 1A:** 55% participants reported changes to BYOD related policies or processes are communicated to staff as part of change management process |
|  | **Study 1B:** *“Technology advances so quickly and cybersecurity issues develop almost daily that you're having to deal and one small weakness can then actually have an impact on your whole network… It's a really interesting industry and environment, one that's changing really quickly…“there's part of a piece of work we're doing over the next six months. We will be revisiting our current state, future state, doing some gap analysis around that and coming up with a digital health strategy for our hospital. Part of that is the end user device piece and understanding what the end user device requirements or BYOD requirements will be.”* P5  ***“****I think that the main challenge is essentially around the constantly evolving technology and threats.”* P1  *“Now there are cyber criminals out there who want to profit and technology's so pervasive, I think we're just exposed to more risk now and we haven't kept up with that level of risk.”* P2  *“One thing that sort of came into my mind is having to maintain compliance especially when writing a policy around device models that the user can bring into the organisation because not all devices would be compatible with most of the applications that need to be provisioned onto that device. And technology is constantly evolving on a daily basis. One day you've got Apple-6, the next day you've got Apple-15. And those applications used that used to work on an Apple-8 or Apple-7 for example would not work on Apple-15. So again having to maintain that level of compatibility with the technology as it's changing is especially challenging in a BYOD environment, especially around developing that policy and the framework around BYOD.”* P6 |
|  | **Study 2A:** No relevant evidence found |
|  | **Study 2B:** *“Our IT team, isn't always well equipped to explain the rationale or doesn't do it in a way that resonates with clinicians and as such, sometimes you can get some tension there. So it's just trying to find a way to communicate the need for these changes and updates and security measures to the clinicians in a way that resonates and is meaningful to them so that they are likely to adopt them and not get angry.”* Doctor1 |
| **R7.02: Staff should be provided regular periodic training to keep them up-to-date with the latest security threats as mitigation methods** | **Study 1A:** 20% participants reported periodic BYOD security training is provided to staff at their hospital |
|  | **Study 1B:** *“I think the challenge is to keep the workforce not just engaged, but well informed of what is an acceptable way of operating and what's not. And user education, I believe, or constant user education, I believe is a challenge, because we learn something, and then if we don't use it, we forget.”* P1  *“I found that, say financial services or an engineering company that has a really strong health and safety culture, we have to redo your training every year and you have to refer and check the policies and say that you've read them. There's a real rigor around that in some of those strongly controlled industries and there's quite a reluctance I think in health to make people do training that's not clinically related. That seems to be an imposition on their time whereas in other industries I found there was a real, you know, from a compliance point of view you need to be refreshed, retrained, keep up to date with your policies but I don't see that as much in health. I actually think that regular training, appropriate to your role and a refresh and a sign off each year that you've done it and you are up to date actually works*” P2  *“Yes, it would be, because the training should be iterative, the training must evolve. It needs to be completed annually. We are going to refresh the course every year, just to introduce a new idea, and we were going to prioritize the risks.. We'd have to probably introduce another item the subsequent year and every subsequent year, just because, as you said, the nature of the risks is evolving and it requires constant education.”* P7 |
|  | **Study 2A:** No relevant evidence found |
|  | **Study 2B: *“****I think the growth at which we're expected to use our personal devices is not matched by the education around this, how to use them safely. Like, I think the shift to being able to do all of these things in our personal devices, certainly has been much quicker than learning all the safety aspects of it. So I think some like concomitant education around that and awareness of the potential risks and strategies to avoid, exposing both our patients and our personal data, would be helpful.”* AHP4  *“So we had to update our training every year…we had to do online modules, which update us about the IT security and other issues related to internet. we have an online learning tool, everyone has a profile, portfolio and on their dashboard they will have training modules there, which will tell them your module has expired and you need to renew it and you need to go back and read it.”* Doctor1 |
| **R7.03: Periodic feedback should be taken from staff, especially clinical BYOD users to inform future strategy by understanding their practices and future requirements** | **Study 1A:** No relevant evidence found |
|  | **Study 1B:** *“Where IT comes in and pushes a solution on the organisation and there's no stakeholder engagement, it's never going to be successful. If the clinicians came to us and said, "This is what we want," or "This is the problem we have. What are the options and what's your recommendation?" And there's buy-in from the clinical part of the business' stakeholders, with that clinical sponsorship, then it's more likely to be a success.”* P7  *“I think the whole concept of the position of chief medical informatics officer, chief nursing, or chief clinical informatics officer, is that the clinical person gets involved and provides meaningful input into policy, which would have probably been a bit difficult before. I think in the past, before we develop these hybrid mixed roles, the clinical people just did not understand yet the other side of it. There hasn't been clinical engagement where there should be, as the people that are going to be using it should give their perspective.”* P3 |
|  | **Study 2A:** No relevant evidence found |
|  | **Study 2B:** No relevant evidence found |
| **R7.04: Skill improvement programs should be offered to both technical and clinical staff to upskill them regarding technology and cater to the change in cybersecurity environment** | **Study 1A:** No relevant evidence found |
|  | **Study 1B:** *“I think it's actually going to become more and more part of their role as digital clinical providers is that they are going to have to factor in some component of their role that allows them to up skill, to train, to maintain that knowledge and skill set around the use of digital technology in the provision of healthcare. Because you can't just expect them to pick up the technology and use it…There's so many considerations that I do that clinicians as a whole will need to have to start to factor in, that technology piece into their roles and whether that means it starts coming into what they're educated at, at a university level, at a hospital level, somewhere it will have to be introduced soon I think.”* P5  *“the appetite to train clinical staff on cybersecurity is quite low, and really cybersecurity needs to have the same sort of profile as OHNS in an organisation. We just need to operationalize cybersecurity education into those sort of OHNS things.”* P7 |
|  | **Study 2A:** No relevant evidence found |
|  | **Study 2B: *“****I think if you make people aware that, there's a problem out there, they'll all try and do the right thing… we implemented (cybersecurity) education into the classroom, so that it becomes part of the early good practice going forward, the hospitals do reinforce the cybersecurity and they do remind people (clinicians) to do that.”* Nurse2 |
| **R7.05: Clinical change champions should be engaged in promoting cybersecurity initiatives among staff, as well as to improve understanding between clinical and IT departments** | **Study 1A:** No relevant evidence found |
|  | **Study 1B:** *“ I would say the hospital itself and quite a few of the medical staff are supportive of doing the right thing. We've got good relationships between the technology and the clinical work groups and we would explain in the future why we have concerns and they would jump on board and we'd get some champions out there who'd speak to it.”* P2  *“We know what works best is the local champion type of thing. So, I'm trying to set up a network of champions for all things, technical, digital, innovative informatics clause, so that there are special interest groups all over the place. So that when you've got any type of thing that you've got to roll out, you can just disperse it down the tree or the roots of the tree, if you like.”* P3 |
|  | **Study 2A:** No relevant evidence found |
|  | **Study 2B:** *“I guess the difference to that is that perhaps junior staff are much more tech savvy than some of the most senior staff, sometimes there's an upward education as well, like where it's the junior staff that actually educate the senior staff in how to do things properly, but then you've got to deal with and address the power balances and fix them and empower the junior staff to be able to do that. And that really requires the senior staff inviting that sort of education.”* Doctor5 |
