# Supplementary material for: A Sociotechnical Approach to Bring-Your-Own-Device Security in Hospitals: Development and Pilot Testing of a Maturity Model Using Mixed Methods Action Research
Source: JMIR Hum Factors. 2025 Aug 13;12:e71912. doi: 10.2196/71912 (PMC12391842; doi:10.2196/71912)
Supplement: Multimedia Appendix 2 [file humanfactors_v12i1e71912_app2.docx]

## Multimedia Appendix 2: Final hBYOD Security Maturity Model Description (Bolded measurable controls/criteria)

| **DIMENSION: TECHNOLOGY** | | |  |
| --- | --- | --- | --- |
| **Domain Name** | **Level** | **Definition** | **Mapped hBYOD**  **framework**  **recommendation** |
| **1. Identity, access and authentication management** | **5** | **Enterprise wide IAM solutions** where multiple, advanced authentication practices such as **adaptive authentication** and **step-up authentication** (for highly sensitive data such as PHI) are used. **Artificial Intelligence/Machine Learning** may be used to observe authentication patterns. User friendly authentication with a **completely federated, automated and unified single sign on** **across all hospital services/applications/systems**, with **continually improving and agile practices**. | R1.07, R2.03, R2.05, R3.01, R3.02, R3.03, R3.04 |
|  | **4** | **Advanced IAM solutions** used with access levels properly defined based on staff roles via **role-based access control**. **True multi factor authentication** is used accommodating multiple factors. This includes something you know, such as password or pin; something you have or something you are, such as **biometric authentication**, which includes facial recognition and fingerprint authentication. **Single sign on used**, though single sign on may be **applicable to only some of the services/applications**, which may even include non-web applications. |  |
|  | **3** | User identity and authentication managed **through Identity and Access Management (IAM) solutions, with a high-level access control definition.** **Simple form of dual factor authentication** maybe implemented. For example, something you know, like a password and something you have, such as a phone or token. **Simple version of single sign on may be used for a few web based applications.** |  |
|  | **2** | **Single authentication factors used**, though with best practices such as use of **unique passwords with high complexity** such as alphanumeric characters with high phrase length. This maybe too burdensome for users – e.g., long passwords, repeated logins, multiple passwords, regular change of passwords. |  |
|  | **1** | Very basic identity/access/authentication management controls implemented such as through single authentication factors such as **simple passwords which are not unique to each individual. No access/application logs maintained** by hospital for services accessed through personal devices. |  |
| **2. Storage and backup** | **5** | **Private cloud used for secure, automatic hospital data backup from BYOD devices**. **Continually improving storage and backup practices** with **no hospital data residing on BYOD device** as **complete application virtualisation or isolation** is achieved across **all hospital services/applications** therefore nullifying chances of data leaks, especially PHI due to BYOD devices. | R1.07, R1.08, R3.11, R3.12, R3.15, R6.01, R6.02, R6.03, R6.04 |
|  | **4** | **Complete isolation of hospital data from personal data and all hospital data** through **containerisation/sandboxing or application-level virtualisation.** **Cloud based services or applications** are extensively used**. Hospital sanctioned storage/backup platforms such as Office 365 or SharePoint may be used as well.** |  |
|  | **3** | All approved **hospital applications to be accessed on BYOD devices through virtualisation**, therefore minimising the risk of data leakage by limiting the need to store hospital data on BYOD devices. Backup leakages may occur via **backups through personal cloud-based storage services like Dropbox or personal cloud like iCloud or Google Drive,** which may result in storage of sensitive hospital information on such services and can therefore lead to data leakages such as unauthorised access. |  |
|  | **2** | Most types of **hospital data stored on staff devices are to be personally managed by staff themselves**. Minimal control over hospital data residing on employee device. **Some degree of logical separation of hospital and personal data** on the device through **device virtualisation**, which allows storage and processing of data within hospital’s own infrastructure rather than the BYOD device itself. Some hospital applications may be accessed through virtualisation and/or use of **Virtual Private Network (VPN),** also providing secure remote access. |  |
|  | **1** | All types of hospital data stored on staff devices are to be personally managed by staff themselves. **No control over hospital data residing on staff device.** **No mechanism to logically separate hospital and personal data on the device.** |  |
| **3. Device security** | **5** | **Complete endpoint protection** and visibility/transparency of hospital data residing on BYOD devices via **advanced Unified Endpoint Management (UEM)/Mobile Device Management (MDM) /Endpoint Detection and Response (EDR) platforms** with capabilities to remotely monitor, detect and respond to complex endpoint/**BYOD device threats in real time**, both in online and offline mode, for all types of devices accessing any type of hospital services/applications or networks. Managing security requirements of all versions of devices or operating systems is easily achievable. **continually improving and agile practices.** | R2.04, R3.05, R3.15, R3.17, R4.01, R5.01, R5.02, R5.03 |
|  | **4** | **Endpoint protection platforms** used to actively monitor, detect, and respond to endpoint threats, but **usage may be limited to certain types of devices or with certain operating system types** and only while connected to the hospital network. Managing security requirements of all versions of devices or operating systems may be achievable, but difficult. |  |
|  | **3** | Moderate ability to manage device security with the ability to formulate **blacklists and whitelists to allow/restrict devices based on certain security policy rules through technologies such as Mobile Device Management (MDM).** |  |
|  | **2** | Hospital has **minimal control to restrict type of device used for BYOD**. **High risk devices such as jailbroken/rooted devices could be restricted**. Other risk prone devices such as those with no passcodes or old devices can be used to access hospital services, applications or networks. Hospital predominantly relies **on manual rules which state what kind of devices can be used for BYOD purposes** and **permissions maybe required before accessing hospital network or services through BYOD devices**. |  |
|  | **1** | Any type of device can be used for BYOD with **no mechanism or technical control to restrict type of device used for BYOD**, meaning devices like jailbroken/rooted, old device or virus/malware prone devices can be used to access hospital services, applications or networks. |  |
| **4. Network security** | **5** | Network security implemented as part of **enterprise cyber-defence system with capability to monitor and scan BYOD devices** **both onsite and remotely in real time for latest threats**. **Software defined networking used. Continually improving and agile practices based on advanced/real time threat intelligence** | R3.08, R3.09, R3.10, R3.12, R3.15, R4.01, R4.02, R5.01 |
|  | **4** | **Detection of abnormal behaviour of data** transmitted to or from the hospital network through **advanced security systems or visualisation software. Secure web gateways** are used to ensure that unsecured traffic, which may be initiated from BYOD devices such as malicious traffic from the web, viruses, or malware, does not enter the internal network of an organisation. |  |
|  | **3** | **Strong firewalls** used with **advanced intrusion prevention/detection systems** which can prevent malware intrusion arising through personal devices, in addition to **network segmentation.** Minimal chance for malicious traffic to enter internal network. Use of **network hardening** best practices to secure hospital network. |  |
|  | **2** | Use of a **separate guest network** or **network segmentation** for personal devices which can only be used for internet access, with restrictions to join core hospital network. **VPN** is used in case of remote access to for connecting personal devices remotely. |  |
|  | **1** | Very basic or primitive vulnerability detection or prevention mechanisms such as **basic firewalls** used which can allow simple malware from personal devices to penetrate hospital networks. All devices, including vulnerable or risk prone devices can connect to the core hospital network. **No mechanism to restrict staff from accessing hospital information (particularly patient information) on unsecure networks or access hospital information remotely in a secure manner.** |  |
| **5. Application security** | **5** | **Complete application virtualisation** with complete separation/isolation between hospital data and personal data. Advanced application security features such as **minimal cache for apps, limit copying of patient data or prevent screenshot functionality especially for sensitive patient data**. **Automated action on unsecure apps or bugs** based on **threat intelligence**. | R1.07, R1.08, R3.06, R3.07, R3.12, R5.01, R5.03 |
|  | **4** | The hospital maintains and updates **application whitelists and blacklists** to ensure no unsecure apps are installed on personal devices involving hospital work. Hospital provides **secure web apps** and a **dedicated secure app store** to download hospital apps. **Advanced application firewalls** used for hospital-based applications which prevent application access on unsafe public networks such as hotspots and therefore protect PHI. |  |
|  | **3** | Hospital may make several **applications or services available through personal devices, but they are ‘in-silos’** make it difficult to manage security requirements. This may make hospital data vulnerable to breaches as vulnerabilities within the apps may go unnoticed. |  |
|  | **2** | Hospital only sanctions **use of basic applications such as email on BYOD devices.** As such, there is still a high risk of using unsecure personal apps as workarounds to carry out hospital work which can put its data, particularly patient data at risk of breaches. |  |
|  | **1** | All **hospital related work can be carried out by employees via personal apps**, including unsecure, risk prone apps which may even involve working with or storage of sensitive patient data as the **hospital has no approved or sanctioned method or applications** for carrying out professional hospital work through personal devices. |  |
| **6. BYOD management automation and control** | **5** | Very high degree of automation and control achieved through use of services such as **Unified Endpoint Management** which provides a **single unified interface for managing all types of devices** existing within the enterprise, such as PCs, laptops, smartphones, tablets, IoT devices, and wearables, which include both BYOD and company-owned devices. It also allows better methods of managing hospital apps/data, confining them to a secure workspace and separating the personal data of caregivers. | R2.02, R3.05, R3.07, R3.11, R3.12, R5.01, R6.01, R6.02 |
|  | **4** | **Application-level control** achieved through technologies such as **Mobile Application Management, containerisation or sandboxing** which automate implementation of BYOD security controls to a **high level by provisioning or controlling access** **to applications/apps** and **automating application data security without requiring device level access.** |  |
|  | **3** | **Device level control** achieved through technologies such **as Mobile Device Management (MDM)** which automate implementation of BYOD security controls to a moderate level through features such as enabling **screen lock or log-off functionalities; encrypting hospital data; securing remote connections through virtual private networks (VPNs); tracking device location; wiping, locking, and securing devices remotely; and whitelisting and blacklisting apps and devices such as jailbroken/rooted devices.** The varied types of operating systems/device types make it difficult for the IT team to manage BYOD. |  |
|  | **2** | **BYOD security controls applied on an ad hoc based on the vulnerabilities or risks** that may occur which limits automation of BYOD security and therefore requires high levels of manual effort |  |
|  | **1** | **BYOD security management based on complete manual efforts** with **no visibility or control over staff activities** on their personal devices when accessing hospital services/information, neither any mechanism for automated enforcement of security policies. |  |
| **7. Clinical communication, photography and file sharing** | **5** | **Dedicated secure clinical communications/collaborations** platform specifically **designed for hospital with integration to all hospital systems** with **very high levels of usability** and **continuous updates and improvements**. | R1.04, R1.07, R1.08, R3.06, R3.13, R3.14, R3.16, R6.01 |
|  | **4** | **Dedicated secure clinical communications/collaborations platform** with **integration to basic health services/platforms** such as electronic medical record or patient management systems with very high levels of usage across all clinician groups. **Patient consent for photography obtained through the platform/application.** |  |
|  | **3** | **Dedicated secure clinical communications/collaborations platform** but with **very limited usage across clinician group** and **only available for certain types of device** (such as smartphones) or OS types (such as iOS). **Clinical communication, photography, and file-sharing is well defined by the relevant policies,** which provides guidance on aspects such as what type of device/s can be used for it, who can use it, where and how patient data can be stored or transferred as well as other relevant staff best practices. **Patient consent for photography mandated** but **obtained via handwritten or complex electronic forms.** |  |
|  | **2** | Certain **secure generic platforms such a Teams** etc. **may be recommended** and generally used but **unsanctioned platforms like WhatsApp are not restricted** from usage. **Patient consent mandated** but only obtained verbally. |  |
|  | **1** | **Hospital staff left on their own** **to decide suitable method for clinical communication**. The **process of clinical communication, clinical photography or file sharing through BYOD devices is undefined. Patient consent for clinical photography not mandated**. |  |
| **DIMENSION: POLICY** | | |  |
| **8.BYOD Strategy** | **5** | A complete **well-defined BYOD security program** established **covering all aspects of BYOD security** which is **agile, periodically audited/updated and continually improving according to the hospital needs, vision, and mission.** | R1.01, R1.03, R1.04, R1.07, R7.01 |
|  | **4** | **A comprehensive BYOD strategy is established and implemented via a BYOD security program**, which provides thorough guidance about BYOD management within the hospital. **Key decisions regarding BYOD such as choosing key BYOD management products are measured against evaluation criteria and evaluated by committees put in place. Regular monitoring and auditing of BYOD security management product vendors against SLA’s.** |  |
|  | **3** | A **dedicated BYOD strategy is established and implemented covering important technical, policy/process or people related aspects of BYOD at a high level.** |  |
|  | **2** | **BYOD strategy defined as part of the general IT security strategy with minimal references and only high-level risks identified pertaining to BYOD.** |  |
|  | **1** | **BYOD security strategy undefined,** which makes it unclear whether it is allowed for staff or not. BYOD not identified as a separate risk. |  |
| **9. BYOD Policy Components** | **5** | **Comprehensive BYOD policy with regular updation,** Change to threat, technology and business landscapes regularly reflected in policy and processes. In addition to **comprehensive communication to all staff groups, due processes established for staff to provide feedback on policy with their active involvement.** | R1.01, R1.02, R2.01, R7.01 |
|  | **4** | Comprehensive **BYOD policy covering all relevant components, but rarely updated**. **Organisational alignment, Legal risks defined**. **Policy covers** **privacy of both hospital/patient and personal data** and provides transparency to users. **Policy communicated with all staff levels.** |  |
|  | **3** | **Dedicated and formal BYOD policy, with basic components** such as acceptable use, incident response, legal risks/responsibilities, disciplinary measures and some clinical related aspects defined. **Policy only covers patient/hospital data privacy without referring to personal data privacy.** The **policy states how the hospital will protect the personal data of staff, if they sign up to use BYOD.** In addition to the intranet, **policy communicated to senior management and clinical representative groups at times of establishment or changes.** |  |
|  | **2** | **IT security policy but with poorly defined BYOD aspects, High level guidance of secure technology use but with clinical or hospital related aspects such as clinical communication, collaboration, storage of patient data undefined. Policy available on the intranet, though not specifically communicated to staff.** |  |
|  | **1** | **No formal BYOD policy in place**, with **no mention of BYOD in the general IT/Security/Acceptable use policy either.** |  |
| **10. Compliance with healthcare regulatory laws and standards** | **5** | **Agile, automated and continually improving process of legal compliance** with change to regulatory landscape regularly reflected in BYOD policy and processes, also keeping in view the future changes. | R1.08, R2.01, R5.04, R5.05, R7.01 |
|  | **4** | **Legal responsibilities associated with BYOD use explained to all relevant parties**, especially users in a comprehensible language. **Monitoring of legal compliance, albeit not very actively**. **Compliance maintained beyond the legislative requirements, at an industry best-practice level.** |  |
|  | **3** | **BYOD associated legal risks properly defined as part of the BYOD policy** but may only be understood by certain sections such as legal team/department, with minimum efforts to make it comprehensible to all parties, especially users. **All relevant departments notified of security events such as breaches.** |  |
|  | **2** | **Reference to associated legislations in policies, but legal risks may not be properly defined or monitored**. **Only major security events maybe referred to certain government departments to fulfill legal obligations.** |  |
|  | **1** | **No legal risks concerned with BYOD use identified. Associated state/federal legislations particularly associated with health data protection not known. No set process for breach notification.** |  |
| **11.Policy enforcement** | **5** | **Complete/comprehensive and automated BYOD policy enforcement** through **advanced technologies with real time measurement system to evaluate security compliance and performance against all BYOD policy aspects, which is readily adaptable and highly agile**. | R2.03, R3.05, R3.14, R4.01, R4.02 |
|  | **4** | A **balanced measurement system to evaluate security compliance and performance** against all policy aspects, but not very adaptive or with minimum agility. |  |
|  | **3** | **A basic/high level evaluation system to report security compliance on BYOD security aspects,** with good chances of breaches of BYOD policy or **associated user behaviour being overlooked.** |  |
|  | **2** | **Minimal verification process for BYOD security compliance/security behaviour through access logs and SIEM services, but only at times of security events such as breaches.** |  |
|  | **1** | **No measurement or verification process to check levels of compliance with BYOD policy** to determine levels of enforcement. **Compliance entirely left to staff goodwill.** |  |
| **12.Incident response/lost device policy** | **5** | **Real time, active, advanced, automated and agile BYOD related security incident** **response** processes based on **advanced threat intelligence and with availability of security team 24x7**. **Capability of resolving most incidents in real time. Incident response plan and relevant processes are regularly audited and updated.** | R4.01, R4.02, R4.03, R5.04, R5.05, R5.06, R5.07, R6.01, R7.01 |
|  | **4** | **Well-defined, documented, and formalised BYOD security incident response plan** with **a highly available security team**. |  |
|  | **3** | **Well defined and documented security incident response plan**, specifically for BYOD related incidents with **end users properly trained to notify of security incidents and a security team to resolve such incidents, though response time may be towards the higher side.** |  |
|  | **2** | **Incident response process may be defined for general security events, but not specifically for BYOD related security events**. **Reliance on external third-party teams to resolve security incidents.** |  |
|  | **1** | **Poor and slow BYOD security incident management as incident response procedure not defined at all. Incidents are responded to in an ad hoc, reactive manner.** |  |
| **13.Lost Device Policy** | **5** | **Selectively wipe off hospital data/apps or sensitive PHI remotely for all device types** and with high degree of automation through technologies such as UEM. Due to proactive security controls and **advanced technical capabilities such as anti-theft or tracking, theft cases are minimised. All lost/theft device incidents are resolved in real-time.** | R3.05, R3.11, R3.12, R3.15, R3.17, R4.03, R5.04, R5.07, R6.01, R6.02, R6.03, R6.04 |
|  | **4** | **Selectively wipe off hospital data/apps or sensitive PHI remotely** (while keeping personal data intact) in case of loss or theft, through **technologies such as containerisation. which do not require complete device access.** **Taking this action may take time or may not be possible for certain device types. Consent is obtained from the owners before taking such an action.** This is in addition to the staff training as specified in level 2. |  |
|  | **3** | **Completely wipe off data (including personal data)** or lock the device remotely in case of loss or theft, though consent is obtained from the owners before doing so **through technologies such as MDM**. This is in addition to the staff training as specified in level 2. **Risk prone devices such as those with no passcode are prevented from accessing hospital services.** |  |
|  | **2** | The **hospital deliberately takes no action on loss/theft of BYOD devices due to staff privacy concerns**, though **staff are informed/trained about its risks and provided a set of recommended steps** such as screen lock, device encryption, biometric/complex authentication, device tracking, setting up screen time outs, as well as notifying IT/security team to prevent hospital data breach. |  |
|  | **1** | **Action to be taken on loss/theft of BYOD devices not defined. Staff also not made aware of risks of PHI breaches due to loss or theft of device.** |  |
| **14.Accountability and governance** | **5** | **Complete accountability and governance established** as part of the BYOD security program with the **roles and responsibilities well defined and communicated to all relevant parties, with continually improving and agile change management governance as part of the program.** | R1.05, R1.06, R1.09, R1.10, R1.12, R2.01, R2.03, R7.01, R7.03 |
|  | **4** | A **formal BYOD security program** established with roles and responsibilities of all groups specified, including formulation of committees such as **steering committee. Inclusion of clinical stakeholders in the decision making process. Complete alignment with the legal, statuary or regulatory requirements.** |  |
|  | **3** | **BYOD security governance established as part of general IT/IT security governance**, with BYOD related aspects covered under the set IT/security governance programs. **Agreement obtained electronically and requires provisioning/configuration of device for BYOD use at time of joining employment and de-provisioning when leaving employment.** |  |
|  | **2** | **Informal governance structure for BYOD management, leadership defined but individual roles not established.** **A generic agreement obtained for safe use of technology e.g; at times of joining employment as part of the sign-up process with no or minimal reference to BYOD.** |  |
|  | **1** | Both staff and management are not aware of their roles and responsibilities concerning BYOD which result in uncoordinated activities. **Governance, roles and responsibilities regarding BYOD management undefined. No formal enrolment or registration process for BYOD use, and as such, no set user agreement in place for using BYOD.** |  |
| **DIMENSION: PEOPLE** | | |  |
| **15. BYOD awareness and training** | **5** | A **comprehensive, regular, and easy to understand BYOD security training is provided to all staff which is tailor made to their clinical role/workflow** and which **accounts for latest BYOD security threats and best practices.** **All modes of training- including online modules, guidelines and reminders via email/flyers, specialised workshops tailor made to staff clinical groups, induction training and face to face meetings used to accommodate trainings at various levels.** | R1.13, R1.14, R2.06, R3.15, R3.17, R5.03, R6.03, R6.04, R7.02 |
|  | **4** | **A comprehensive BYOD security training** is provided which provides **practical/case-based training on how to use BYOD securely in day-to-day clinical practice, but it may not be regularly updated, or tailor made to staff roles**. **Specialised workshops providing practical case-based training** held to explain safe use of personal devices to all staff groups, in addition to other modes. |  |
|  | **3** | A **dedicated BYOD security training is provided covering important BYOD related aspects** such as device requirements for BYOD, clinical communication, clinical photography, patient data storage and backup on personal devices, acceptable use, personal data privacy and relevant laws/legislations. **An online, module-based BYOD security training provided via intranet which requires completion of assessment such as quiz to check knowledge levels.** |  |
|  | **2** | **Generic IT security training provided with some references to safe BYOD security practices** which aids in understanding obligations at a high level. **Guidelines or reminders supplied to staff via email or posters/flyers to explain basic do’s and don’ts in terms of BYOD use. Induction or orientation training may also have references to cybersecurity.** |  |
|  | **1** | BYOD security awareness across the hospital is completely dependent on the staff’s awareness at the individual level, **with no IT security training designed for staff**. Staff rely on their individual awareness from sources such as news, security blogs, social media, academic research etc. No training material is disseminated by hospital management. |  |
| **16. Training importance** | **5** | **BYOD security training is regular, mandatory and paid for all staff,** with a dedicated paid time allotted for completing training to maintain staff interest. Advanced training could be kept optional and incentivised. | R1.15 |
|  | **4** | **BYOD security training is mandatory, but unpaid** which may lead to staff resistance especially from clinical staff who may be overloaded with work. |  |
|  | **3** | **A dedicated BYOD security training is highly recommended** with regular engagement with staff explaining its importance with certain staff overlooking it due to work overload or other reasons. |  |
|  | **2** | **An optional/generic IT security training is provided which is accessible via intranet** with moderate levels of engagement from management to complete the training. |  |
|  | **1** | **Management completely relies on cybersecurity awareness at the individual level** with no stress upon improving cyber knowledge among staff. |  |
| **17. Management support** | **5** | **Very high level of ongoing management support with no budget or other constraints** to advance BYOD security management through technology, policy as well as people management improvements leading to an **agile and continuously improving security posture.** | R1.11, R1.12, R1.15 |
|  | **4** | High levels of management support with a **complete recognition and prioritisation from senior management in the role of BYOD management in improving cybersecurity posture of the hospital**, with **high budget set for such initiatives leading to technology, policy and people management improvements to meet industry best practices** but **may not be agile or continually improving at a greater pace**. Users are also supported by providing them with tools and/or costs required to protect patient data residing on their devices. |  |
|  | **3** | Moderate management support with **cybersecurity set on high priority from senior management but inadequate budget to achieve the required best practices,** particularly with regards to BYOD security management. |  |
|  | **2** | Limited management support for BYOD security management, with a **recognition of the need to improve cybersecurity posture, though BYOD security may not be understood or recognised by senior management**. |  |
|  | **1** | Lack of support from management for cybersecurity initiatives, with **no set budget for IT security or BYOD management.** |  |
| **18. Stakeholder involvement** | **5** | **Regular and periodic staff feedback taken through established processes for BYOD strategy improvements** **with all stakeholder groups** such as IT department, clinical leadership, risk advisory or legal team, clinical staff, telecom department, and other relevant staff who closely collaborate and participate in the process. | R1.05, R1.06, R1.09, R1.10, R7.03 |
|  | **4** | **Clinical staff feedback taken into consideration for BYOD strategy improvements**, in addition to the IT team and clinical leadership, though it **may not be regular.** |  |
|  | **3** | BYOD strategy may include **some level of involvement from clinical leadership** in developing BYOD strategy, but staff not consulted. |  |
|  | **2** | **BYOD strategy entirely handled by IT department** **and/or the executive group** as part of the general IT strategy with no involvement of any user groups/clinical stakeholders. |  |
|  | **1** | **BYOD security matters all handled individually as they come**, with no set process of stakeholder involvement. |  |
| **19. Security culture** | **5** | **A safe and continually improving security culture** across the whole hospital with **agile change management practices**, where all levels of management and staff are **adaptive to proactively accommodate any changes in threat, business, or regulatory landscape**. | R1.05, R1.06, R1.09, R1.10, R1.11, R1.12, R2.01, R3.14, R7.01, R7.02, R7.03, R7.04, R7.05 |
|  | **4** | **A proactive security culture** where staff groups are well aware and with **high levels of trust and engagement between staff groups,** particularly IT and clinical user groups. Deep commitment towards improvement in cyber hygiene across the respective departments by staff groups. **Flexibility to accommodate all types of users**, such as with no, minimal or high BYOD use. |  |
|  | **3** | **A general realisation and commitment from staff towards patient data privacy**. However, **high degree of departmental influence** due to top-down influence leading to resistance among sections of clinical staff groups to changes in security processes or controls. |  |
|  | **2** | **A reactive approach taken by hospital with an effort to respond to present and past BYOD security threats**. Though patient data privacy is viewed as important, but workarounds **are common to satisfy important needs of users, particularly related to patient care due to lack of usable processes/methods**. |  |
|  | **1** | **Patient data privacy not viewed as important by staff,** with unsecure practices common among them when using personal devices and overlooked by management. |  |
| **20. Usability and clinical productivity** | **5** | User-friendly security processes with high levels of usability with use of technologies such as **unified single sign on across the whole organisation** and for all types of devices, removing need for practices such as repeated logins or change of passwords and therefore completely streamlines clinical workflow, leading to massive productivity improvements. A **secure, yet unrestrictive environment where the hospital allows all services/applications through BYOD devices of any type, both remote and onsite.** | R1.04, R1.07, R1.09, R3.03, R3.04, R3.05, R3.07, R3.13, R3.14 |
|  | **4** | **Single sign on used for most applications/services** and may include even non web applications leading to productivity improvements and better patient care. **Enterprise productivity apps** are used which may include a range of patient care related or other productivity apps made available through a hospital-based app store. |  |
|  | **3** | Use of technologies such as **single sign on** which improves usability and clinical workflow, though it may be **limited to only a few applications. Moderately restrictive environment** where a few important services/applications such as EMR, alerts system and drug reference may also be allowed through BYOD devices, in addition to basic services like email, though some restrictions may apply in terms of device or operating system types. |  |
|  | **2** | Reduced complexity by providing staff options to achieve security-usability balance – such as increasing password complexity and removing need to change passwords repeatedly or vice versa. **Each application may be required to authenticate separately,** though the same password may be used due to the use of reduced sign on. **Highly restrictive environment only allowing staff to carry out basic work through BYOD devices through services such as email.** |  |
|  | **1** | **Very complex/cumbersome security processes such as logins setup which are unusable by staff which hamper the productivity, especially of clinical staff and may delay patient care. Staff may use workarounds** instead to avoid complexity. **Rigorously restrictive environment** only allowing staff to use internet services such as hospital Wi-Fi through BYOD devices, with no access to hospital services. |  |
| **21. Expertise and skills improvement** | **5** | A complete/advanced skillset within the hospital to **autonomously handle all BYOD security affairs**, with a **continuous and regular skills improvement process** to handle latest or evolving threats. | R1.11, R7.01, R7.03, R7.04, R7.05 |
|  | **4** | High level of expertise across all technical, policy and people management domains to manage BYOD security affairs which includes a **broad cybersecurity technical team, health information management professionals, legal/risk advisory groups, prominent clinician groups etc.** with an extensive knowledge of BYOD management. **Skills improvement programs for employees**, but they are not properly incentivised. |  |
|  | **3** | **Dedicated IT security personnel such as CISO/IT security manager to manage BYOD security management**, but with limited staff. **Skills improvement is driven primarily by the IT staff through certifications etc.,** |  |
|  | **2** | **Cybersecurity expertise may lie with certain individuals within IT department, though there is no separate portfolio such as CISO, cybersecurity officer, IT security manager etc. to manage BYOD security affairs.** |  |
|  | **1** | **No or inadequate cybersecurity expertise/skill within the hospital to manage BYOD security affairs. No set process or mechanism to enhance skills or improve knowledge of IT team regarding BYOD management.** |  |
