# Supplementary material for: A Sociotechnical Approach to Bring-Your-Own-Device Security in Hospitals: Development and Pilot Testing of a Maturity Model Using Mixed Methods Action Research
Source: JMIR Hum Factors. 2025 Aug 13;12:e71912. doi: 10.2196/71912 (PMC12391842; doi:10.2196/71912)
Supplement: Multimedia Appendix 3 [file humanfactors_v12i1e71912_app3.docx]

## Multimedia Appendix 3: Maturity Assessment Survey

**WELCOME**   Welcome and thank you for your interest in participating in this study. 
 
Hospital BYOD or ‘Bring Your Own Device’ refers to staff use of personal devices such as laptops, tablets, or phones for hospital related work. BYOD management is an important aspect of hospital cybersecurity, with implications for patient and staff privacy and security. 
 
The project researchers have developed a novel, first of its kind BYOD security maturity model considering unique healthcare socio-technical factors and therefore exclusively developed for hospitals. 
 
The primary aim of this study is to test the utility of the proposed model to assess the BYOD security maturity of a hospital and refine the model based on your feedback. As such, this survey asks you to rate your hospital’s BYOD security by using a rating scale. The survey also invites your feedback on the ratings scale itself, so that researchers can refine it. 
 
For the participating hospital, this study will not only capture the current BYOD security posture, it will also provide a roadmap towards improvement through a series of prioritised recommendations, based on findings from the study. This will enable the establishment of a well-informed and contextualised BYOD strategy. 
 
The survey will take approximately 15-25 minutes to complete. No personal information will be gathered and your responses to the questions will be kept completely confidential.  
 
PLEASE NOTE THAT ALL QUESTIONS ASKED ARE FOR THE ‘X’ HOSPITAL, AND NOT ANY OTHER HOSPITALS YOU MAY BE WORKING IN.

| Page Break |  |
| --- | --- |

A Plain Language Statement (PLS) has been provided below which provides detailed information about the project and the nature of your participation. Please click to access.            [Plain Language Statement_BYOD Survey](https://drive.google.com/file/d/1Ht6v9GIe3T7tE1XdjChaELdM3jnyhxdi/view?usp=sharing)
 1.  I consent to participate in this project, the details of which have been explained to me, and I have been provided with a written plain language statement to keep. 2. I understand that the purpose of this research is to investigate BYOD security management in hospitals. 3. I understand that my participation in this project is for research purposes only. 4. I acknowledge that the possible effects of participating in this research project have been explained to my satisfaction. 5. I understand that my participation is voluntary and that I am free to withdraw from this project anytime without explanation or prejudice and to withdraw any unprocessed data that I have provided. 6. I understand that the data from this research will be stored at the University of Melbourne and will be destroyed after 5 years. 7. I have been informed that the confidentiality of the information I provide will be safeguarded subject to any legal requirements; my data will be password protected and accessible only by the named researchers. 8. I understand that a record of my consent will be retained by the researcher.   *Please proceed forward if you wish to participate.*

| Page Break |  |
| --- | --- |

   The following questions gather generic information about your current role in the hospital

Please select the most relevant profession group you belong to:

- IT Managerial Role (CIO/CISO/CTO/IT Manager/Cybersecurity Manager etc.) (1)
- Clinical Informatics Role (CMIO/CNIO/Health Information Manager etc.) (2)
- Senior Executive Role (CEO/Director etc.) (3)
- General IT (4)
- General Clinician (5)
- Legal team/Policy development (6)
- Other: Please state (7) __________________________________________________

Q3 1. Your current role/title on which your responses are based (e.g. CIO/CMIO/Consultant etc.)

________________________________________________________________

Q76 The number of years of service in this hospital/hospital group, on which your survey responses are based:

- 0-5 years (1)
- 6-10 years (2)
- 10+ years (3)

| Page Break |  |
| --- | --- |

**PART 1: BYOD SECURITY MATURITY ASSESSMENT & FEEDBACK**


The proposed model has 22 domains or areas, relating to one of three dimensions: People, Policy and Technology (PPT) that represent several technical, social and managerial aspects of BYOD security. You have to rate your health service/hospital with respect to each of the specified 22 domains from maturity level 1 to level 5. A description is provided for each level, for which you have to choose the level that your hospital most conforms with (there is an option to select, if you feel you can’t answer a particular question). Please note that the hospital must conform with all controls or capabilities mentioned in the description to be deemed at that particular level.


Furthermore, feedback is sought for any scope of improvement with respect to the accuracy, applicability, clarity of language and completeness of the model. Please proceed ahead to start the assessment.

| Page Break |  |
| --- | --- |

**EXAMPLE DOMAIN ASSESSMENT (Only 1 included as sample)**

**DIMENSION: BYOD TECHNOLOGY**
**DOMAIN 1: IDENTITY, ACCESS AND AUTHENTICATION MANAGEMENT**

Which level best describes your hospital's BYOD security control/response for the domain 'Identity, Access and Authentication Management'?

- Level 1 - Very basic identity/access/authentication management controls implemented such as through single authentication factors such as simple passwords which are not unique to each individual. No access/application logs maintained by hospital for services accessed through personal devices. (1)
- Level 2 - Single authentication factors used, though with best practices such as use of unique passwords with high complexity such as alphanumeric characters with high phrase length. This maybe too burdensome for users – e.g., long passwords, repeated logins, multiple passwords, regular change of passwords. (2)
- Level 3 - User identity and authentication managed through Identity and Access Management (IAM) solutions, with a high-level access control definition. Simple form of dual factor authentication maybe implemented. For example, something you know, like a password and something you have, such as a phone or token. Simple version of single sign on may be used for a few web based applications. (3)
- Level 4 - Advanced IAM solutions used with access levels properly defined based on staff roles via role-based access control. True multi factor authentication is used accommodating multiple factors. This includes something you know, such as password or pin; something you have or something you are, such as biometric authentication, which includes facial recognition and fingerprint authentication. Single sign on used, though single sign on may be applicable to only some of the services/applications, which may even include non-web applications. (4)
- Level 5 - Enterprise wide IAM solutions where multiple, advanced authentication practices such as adaptive authentication and step-up authentication (for highly sensitive data such as PHI) are used. Artificial Intelligence/Machine Learning may be used to observe authentication patterns. User friendly authentication with a completely federated, automated and unified single sign on across all hospital services/applications/systems, with continually improving and agile practices. (5)
- Don't know/Can't determine (6)

Q48 For this domain, please suggest any feedback for improvement, in terms of the accuracy, applicability or clarity of language of the described levels if applicable?

________________________________________________________________

________________________________________________________________

________________________________________________________________

________________________________________________________________

________________________________________________________________

| Page Break |  |
| --- | --- |

**TABLE 1** ***Hospital BYOD security maturity model: outline of dimensions and domains***  **Technology** : 1.Identity, access and authentication management; 2. Storage and backup;
 3. Device security; 4. Network security; 5. Application security; 6. BYOD management automation and control; 7. Clinical communication, photography and file sharing

**Policy** : 1. BYOD Strategy; 2. BYOD Policy Components; 3. Compliance with healthcare regulatory laws and standards; 4. Policy enforcement 5. Incident response process; 6. Lost device policy; 7. Accountability and governance

**People**: 1.BYOD security awareness and training coverage; 2. Training dissemination; 3. Training importance; 4. Management support; 5. Stakeholder involvement; 6. Security culture; 7. Clinical productivity and usability; 8. Expertise and skills improvement

Do you consider any of the above listed 22 domains of the maturity model as irrelevant or unnecessary in terms of hospital BYOD security? (See Table 1)

- Yes (Please state domain name/s and also provide reason) (1) __________________________________________________
- No (2)

Do you find any important domain/s relevant to hospital BYOD security missing from the maturity model? (See Table 1)

- Yes (Please name all and state reason) (1) __________________________________________________
- No (2)

Do you consider all domains to be correctly placed/mapped with respect to the three dimensions (People, Policy, Technology)? (See Table 1)

- Yes (1)
- No (2)

*Skip To: Q55 If Do you consider all domains to be correctly placed/mapped with respect to the three dimensions (P... = Yes*

In the text box given below, please state which domains can be moved and to which dimension (e.g. from People to Policy, Policy to Technology etc.)?

________________________________________________________________

________________________________________________________________

________________________________________________________________

________________________________________________________________

________________________________________________________________

| Page Break |  |
| --- | --- |

|  |
| --- |

**BYOD SECURITY PRIORITISATION - TECHNOLOGY**
Based on your hospital needs and capability, rank the following BYOD technology domains in order of their importance or priority - top to bottom? (Drag & Drop)

______ Identity, access and authentication management (1)

______ Storage and backup security (2)

______ Device security (4)

______ Network security (5)

______ Application security (6)

______ BYOD management automation and control (7)

______ Clinical communication, photography and file sharing (8)

| Page Break |  |
| --- | --- |

|  |
| --- |

**BYOD SECURITY PRIORITISATION - POLICY** Based on your hospital needs and capability, rank the following BYOD policy domains in order of their importance or priority - top to bottom? (Drag & Drop)

______ BYOD strategy (9)

______ BYOD policy components (10)

______ Compliance with healthcare regulatory laws and standards (11)

______ Policy enforcement (12)

______ Incident response process (13)

______ Lost device policy (14)

______ Accountability and governance (15)

| Page Break |  |
| --- | --- |

|  |
| --- |

**BYOD SECURITY PRIORITISATION – PEOPLE** Based on your hospital needs and capability, rank the following BYOD people domains in order of their importance or priority - top to bottom? (Drag & Drop)

______ BYOD security awareness and training (16)

______ Training dissemination (17)

______ Training importance (18)

______ Management support (19)

______ Stakeholder involvement (20)

______ Security culture (21)

______ Clinical productivity and usability (22)

______ Expertise and skills improvement (23)

| Page Break |  |
| --- | --- |

You have completed the maturity assessment survey. In case you want to go back to any question, please use the navigation buttons at the bottom.

**End of Block: Default Question Block**
