# Supplementary material for: A Sociotechnical Approach to Bring-Your-Own-Device Security in Hospitals: Development and Pilot Testing of a Maturity Model Using Mixed Methods Action Research
Source: JMIR Hum Factors. 2025 Aug 13;12:e71912. doi: 10.2196/71912 (PMC12391842; doi:10.2196/71912)
Supplement: Multimedia Appendix 4 [file humanfactors_v12i1e71912_app4.docx]

## Multimedia Appendix 4: Maturity Model Revisions.

Participants’ feedback on potential improvements with respect to the language/content and structure of the maturity model levels was also captured. This feedback used to further refine the model.

**Content-based feedback and revisions**

Table A provides details of the content-based feedback provided by study participants about the maturity model and the corresponding revisions made.

| **DOMAIN NAME & LEVEL** | **PREVIOUS DEFINITION** | **PARTICIPANT FEEDBACK COMMENTS** | **REVISED DEFINITION** |
| --- | --- | --- | --- |
| **Storage and backup security (Level 3)** | All approved hospital applications to be accessed on BYOD devices through virtualisation, therefore minimising the risk of data leakage by limiting the need to store hospital data on BYOD devices. Backup leakages may occur via backups through personal cloud-based storage services like Dropbox or personal cloud like iCloud or Google Drive. | *“Impact of using Dropbox, Google Drive on BYOD should be explained”* (Digital Health Partner - Clinical) | All approved hospital applications to be accessed on BYOD devices through virtualisation, therefore minimising the risk of data leakage by limiting the need to store hospital data on BYOD devices. Backup leakages may occur via backups through personal cloud-based storage services like Dropbox or personal cloud like iCloud or Google Drive, which may result in storage of sensitive hospital information on such services and can therefore lead to data leakages such as unauthorised access. |
| **Storage and backup security (Level 2)** | Most types of hospital data stored on staff devices are to be personally managed by staff themselves. Minimal control over hospital data residing on employee device. Some degree of logical separation of hospital and personal data on the device through device virtualisation, with some hospital applications accessed through Virtual Private Network (VPN) and/or virtualisation. | “*Virtualisation is a technical term which needs explanation/definition”* (Director Data Governance and Security) | Most types of hospital data stored on staff devices are to be personally managed by staff themselves. Minimal control over hospital data residing on employee device. Some degree of logical separation of hospital and personal data on the device through device virtualisation, which allows storage and processing of data within hospital’s own infrastructure rather than the BYOD device itself. Some hospital applications may be accessed through virtualisation and/or use of Virtual Private Network (VPN), also providing secure remote access. |
| **Device security (Level 2)** | Hospital has minimal control to restrict type of device used for BYOD. High risk devices such as jailbroken/rooted devices could be restricted, however other risk prone devices such as those with no passcodes or old devices can be used to access hospital services, applications or networks. | *“There may be different rules, such as staff needing ‘permission’ to add their device to our network Should that be specified here?”* (Director Data Governance and Security) | Hospital has minimal control to restrict type of device used for BYOD. High risk devices such as jailbroken/rooted devices could be restricted. Other risk prone devices such as those with no passcodes or old devices can be used to access hospital services, applications or networks. Hospital predominantly relies on manual rules which state what kind of devices can be used for BYOD purposes and permissions maybe required before accessing hospital network or services through BYOD devices. |
| **Clinical communication, photography and file sharing (Level 3)** | Dedicated secure clinical communications/collaborations platform but with very limited usage across clinician group. Patient consent for photography mandated but obtained via handwritten or complex electronic forms. | “*Needs to explain how clinical communication may vary between BYOD by staff i.e. phone vs tablet – Also, many photos are taken on phones and then ‘transferred’ to other BYOD device types.”* (Director Data Governance and Security) | Dedicated secure clinical communications/collaborations platform but with very limited usage across clinician group and only available for certain types of devices (such as smartphones) or OS types (such as iOS). Clinical communication, photography, and file-sharing is well defined by the relevant policies, which provides guidance on aspects such as what type of device/s can be used for it, who can use it, where and how patient data can be stored or transferred as well as other relevant staff best practices. Patient consent for photography mandated but obtained via handwritten or complex electronic forms. |

Table A: Maturity model content-based feedback and revisions

**Structural feedback and revisions**

Survey participants were also asked to provide feedback relating to the structure of the hospital BYOD security maturity model. Firstly, participants were asked whether any of the domains have been wrongly mapped to the three PPT dimensions and if yes, what dimension can it be mapped to. Responding to the question, all participants stated that the mentioned dimensions were mapped correctly. Secondly, participants were asked whether any additional domain relevant to BYOD security can be added to the maturity model to which all responded ‘no’. Lastly, participants were also asked whether any of the mentioned domains were found unnecessary. One of the technology managers stated that domain 15 “BYOD security awareness and training coverage” and domain 16 “training dissemination” overlapped and therefore can be combined into one domain. Upon further deliberation and investigation, it was found that these domains are closely related, so they were combined into one domain namely “BYOD security awareness and training”. Table B provides a description of the updated domain levels for the newly created domain.

| **New Domain Name** | **Level** | **New Definition** | **Mapped hBYOD framework recommendation** |
| --- | --- | --- | --- |
| **BYOD security awareness and training** | **5** | A comprehensive, regular, and easy to understand BYOD security training is provided to all staff which is tailor made to their clinical role/workflow and which accounts for latest BYOD security threats and best practices. All modes of training- including online modules, guidelines and reminders via email/flyers, specialised workshops tailor made to staff clinical groups, induction training and face to face meetings used to accommodate trainings at various levels. | **R1.13, R1.14, R2.06, R3.15, R3.17, R5.03, R6.03, R6.04, R7.02** |
|  | **4** | A comprehensive BYOD security training is provided which provides practical/case-based training on how to use BYOD securely in day-to-day clinical practice, but it may not be regularly updated, or tailor made to staff roles. Specialised workshops providing practical case-based training held to explain safe use of personal devices to all staff groups, in addition to other modes. |  |
|  | **3** | A dedicated BYOD security training is provided covering important BYOD related aspects such as device requirements for BYOD, clinical communication, clinical photography, patient data storage and backup on personal devices, acceptable use, personal data privacy and relevant laws/legislations. An online, module-based BYOD security training provided via intranet which requires completion of assessment such as quiz to check knowledge levels. |  |
|  | **2** | Generic IT security training provided with some references to safe BYOD security practices which aids in understanding obligations at a high level. Guidelines or reminders supplied to staff via email or posters/flyers to explain basic do’s and don’ts in terms of BYOD use. Induction or orientation training may also have references to cybersecurity. |  |
|  | **1** | BYOD security awareness across the hospital is completely dependent on the staff’s awareness at the individual level, with no IT security training designed for staff. Staff rely on their individual awareness from sources such as news, security blogs, social media, academic research etc. No training material is disseminated by hospital management. |  |

Table B: Revised domain - BYOD security awareness and training
