# Supplementary material for: A Sociotechnical Approach to Bring-Your-Own-Device Security in Hospitals: Development and Pilot Testing of a Maturity Model Using Mixed Methods Action Research
Source: JMIR Hum Factors. 2025 Aug 13;12:e71912. doi: 10.2196/71912 (PMC12391842; doi:10.2196/71912)
Supplement: Multimedia Appendix 5 [file humanfactors_v12i1e71912_app5.docx]

## Multimedia Appendix 5: Workshop Structure and Brainstorming Notes.

Table: Workshop Structure

| **Time** | **Activity and Description** | **Materials involved/provided** |
| --- | --- | --- |
| **0:00 – 0:05**  **(5 min)** | **Introduction presentation**: Lead researcher presented the background, aim, significance and process of the study. High level overview of current maturity ratings and top 5 priority domains based on pre-workshop assessment were also presented. | PowerPoint presentation shared via Zoom |
| **0:05 – 0:30**  **(25 min)** | **Group brainstorming session:** Participants divided into two groups (clinical and technical) in Zoom breakout rooms. Group brainstorming session was held through MURAL^TM^ to identify the main challenges and recommendations (based on maturity model) in the hospital associated with the priority domains, with each group taking its own view. | Online MURAL^TM^ worksheet, electronic/PDF version of maturity model |
| **0:30 – 1:20**  **(50 min)** | **Group presentations and follow up discussion:** Each group’s spokesperson presented key challenges and recommendations in the Zoom main room, followed by an open discussion where the other group provided feedback, questioned feasibility, and exchanged perspectives on proposed solutions. | MURAL^TM^ worksheets, presentations shared via Zoom |
| **1:20 – 1:30**  **(10 min)** | **Conclusion and vote of thanks:** The project team concluded with a vote of thanks to all participants and described the next steps in the project. |  |

##
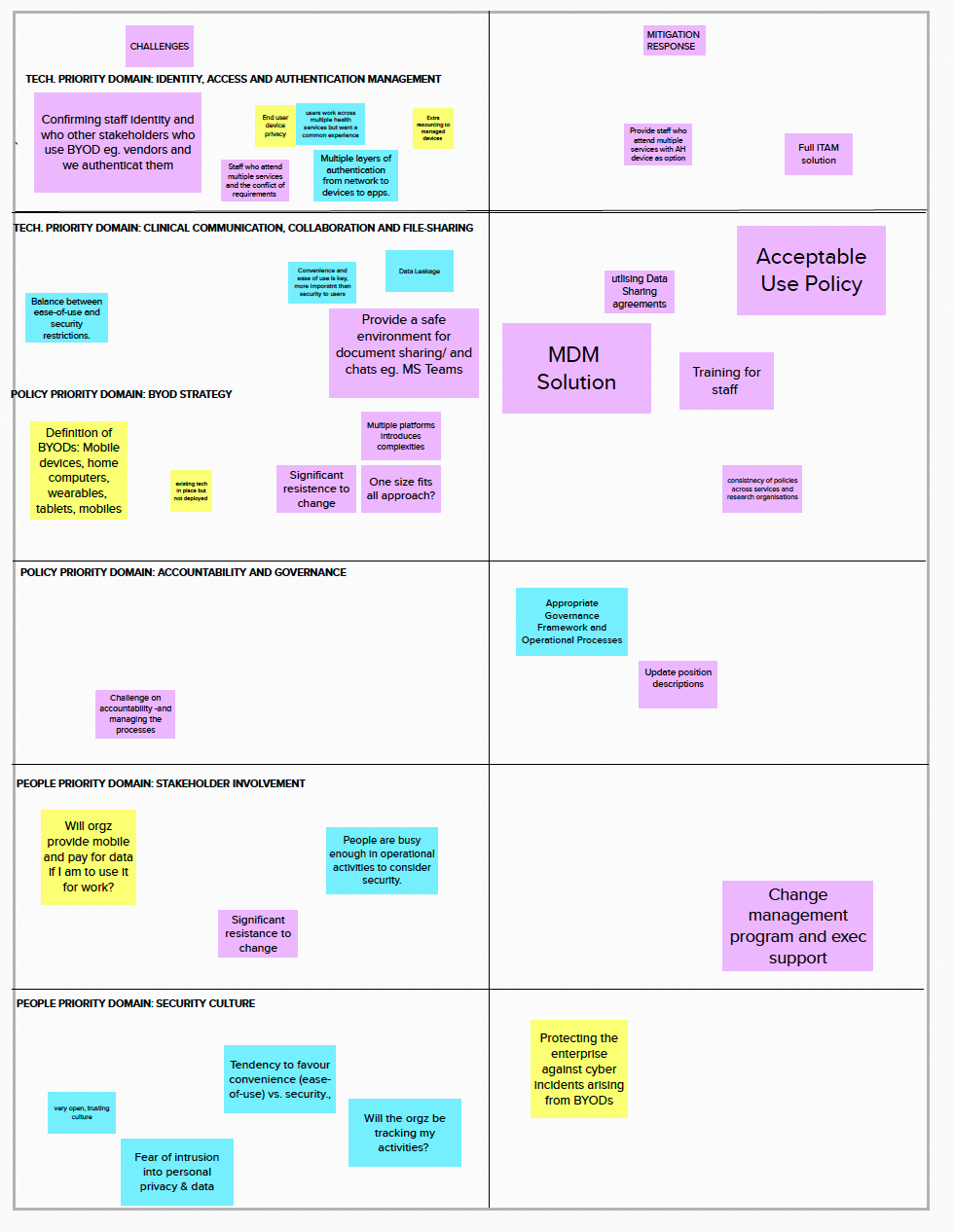


Figure:: IT management group's brainstorming activity notes


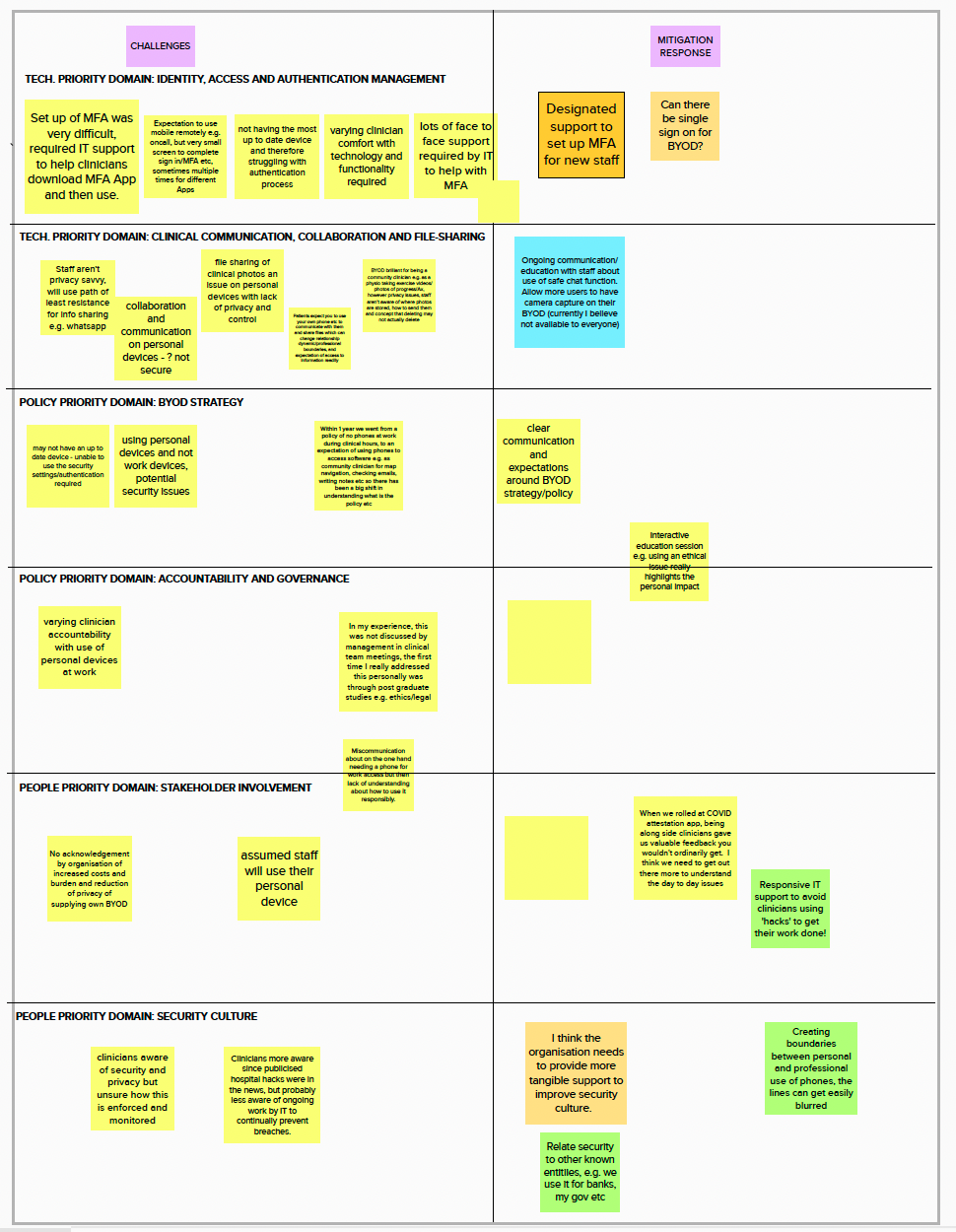


Figure:: Clinical representative user group's brainstorming activity note
